# Seasonal variation in home blood pressure: findings from nationwide web-based monitoring in Japan

**DOI:** 10.1136/bmjopen-2017-017351

**Published:** 2018-01-05

**Authors:** Toshiyuki Iwahori, Katsuyuki Miura, Keiichi Obayashi, Takayoshi Ohkubo, Hiroshi Nakajima, Toshikazu Shiga, Hirotsugu Ueshima

**Affiliations:** 1 Department of Public Health, Shiga University of Medical Science, Otsu, Japan; 2 OMRON Healthcare Co., Ltd, Muko, Japan; 3 Center for Epidemiologic Research in Asia, Shiga University of Medical Science, Otsu, Japan; 4 OMRON Corporation, Kizugawa, Japan; 5 Department of Hygiene and Public Health, Teikyo University School of Medicine, Tokyo, Japan

**Keywords:** public health, hypertension, epidemiology

## Abstract

**Objectives:**

Our aim was to assess seasonal variation in home blood pressure (BP) among free-living nationwide participants using home BP values accumulated from a web-based healthcare platform established in Japan.

**Settings:**

An observational study. OMRON Healthcare Co., Ltd. has been developing web-based personal healthcare record systems in Japan since November 2010; over two million voluntary participants had joined this platform in September 2015. Nationwide home BP measurements made by oscillometric-type electronic sphygmomanometers from over 110 000 voluntary participants have been transmitted to the system from devices.

**Participants:**

Seasonal variation in home BP was evaluated among 64 536 (51 335 men, 13 201 women; mean age 52.9 years) free-living nationwide users for whom data were automatically and simultaneously transmitted to the system from devices.

**Primary outcome measures:**

Mean monthly and weekly home BP.

**Results:**

In multiple regression analysis, the relationship between BP and temperature was a significant inverse association, independent of age, gender and geological locations. Highest and lowest BP was observed in December and July, respectively. Substantial seasonal differences in the mean values of morning and evening home systolic BP between summer and winter were 6.2 mmHg and 5.5 mmHg in men, and 7.3 mmHg and 6.5 mmHg in women. Seasonal variation was a little greater in older (7.3 mmHg in men, 8.7 mmHg in women) than in younger individuals (5.8 mmHg in men, 6.5 mmHg in women). BP from February to July was approximately 1.5 mmHg lower than the value from August to December.

**Conclusions:**

A web-based healthcare platform has enabled easier monitoring of population-wide BP. Tighter BP control is necessary in winter than in summer, and especially in a colder climate toward winter than toward summer. New technologies using web-based self-monitoring systems for health-related indexes are expected to initiate a new phase of cardiovascular disease prevention and public health promotion.

**Strengths and limitations of this study**

- Frequent nationwide home BP monitoring by a web-based platform enabled analysis of seasonal variation in home BP showing monthly and weekly changes in a large sample (>60 000) from a non-medical setting.
- Nationwide home BP data were automatically collected and simultaneously transmitted electronically to the web-based home BP monitoring platform, which minimised the selection bias and reflected the casual BP values in the real world.
- The majority of the participants might have been health conscious individuals.
- Timing and frequency of the measurement, and the room temperature during home BP measurement remain unknown.

## Introduction

The management of blood pressure (BP) is one of the most important strategies for the prevention and management of cardiovascular diseases (CVDs) around the world.^1–6^ Although BP management has been generally based on BP values measured in the clinical setting by medical professionals, home BP monitoring by individuals is becoming increasingly important in diagnosis, and in the control of arterial hypertension.^4–9^

Electronic devices for home BP measurement were developed in the 1990s. Companies that manufacture devices for home BP measurement tend to be concentrated in the East Asian region (Japan, South Korea and Taiwan). Japan is unique in its high coverage of home BP measurement; out of approximately 50 million households, nearly 20 million devices were estimated to exist according to the sales records of BP monitors sold by OMRON Healthcare Co., Ltd. (Kyoto, Japan) in 2012.

Under these conditions, in 2010 OMRON Healthcare Co., Ltd., a healthcare device manufacturer, developed a web-based healthcare platform that consumers can join voluntarily after purchasing one of its products. OMRON Healthcare Co., Ltd. recently developed devices for home BP measurement send all measured values electronically to a web-based database via personal computers or mobile phones. From 2010 to 2015, more than two million people in Japan have voluntarily participated in this web-based healthcare platform. Nowadays, these newly established technologies and platforms enable real-world data to be collected simultaneously and show the possibilities of detecting useful population-wide information to help provide new approaches for public health authorities.

Multiple observational studies have examined the seasonal variation in BP in clinical settings^10^; however, there are no reports on seasonal variation using a large nationwide sample of home BP monitoring data from non-medical and uncontrolled settings. Thus, our aims were to assess seasonal variation in home BP among a nationwide sample of free-living participants using real-world data collected by this web-based healthcare platform, and to suggest applications for public health policy making.

## Materials and methods

### Web-based personal healthcare record systems

OMRON Healthcare Co., Ltd. develops web-based personal healthcare record systems. One of its application services is called WellnessLINK, which was deployed on 1 November 2010. The participants can freely register and leave the web-based healthcare platform at any time. WellnessLINK uses many kinds of healthcare devices connected to the internet via personal computers and mobile phones to transmit various types of healthcare data to a database.

The WellnessLINK system records BP values measured at home using oscillometric-type electronic sphygmomanometers, including an upper arm BP monitor (HEM-7510C, HEM-7500F, HEM-7324C, HEM-7320F, HEM-7280C, HEM-7270C, HEM-7250-IT; OMRON Healthcare Co., Ltd.), an upper arm BP monitor with an automatic cuff wrapping system (HEM-1025; OMRON Healthcare Co., Ltd.), and a wrist BP monitor (HEM-6321T, HEM-6320T, HEM-6310F, HEM-6300F; OMRON Healthcare Co., Ltd.). These devices can be shared with family members by differentiating the user with identification numbers set on each device. All devices have been validated and approved by the Ministry of Health, Labour and Welfare, Japan,^11^ and are certified as having been adjusted to the ISO/IEC 81 060–2 standards.^12^ The users receive an instruction manual for BP measurement based on the Japanese Society of Hypertension Guidelines for Self-Monitoring of Blood Pressure at Home.^7^ Users then access the website via their personal computer or mobile phone, and register after approving a privacy policy agreement. For personal computer users, BP data are transmitted to the system via the internet by connecting the sphygmomanometer using a universal serial bus (USB) port, near field communication (NFC), Bluetooth, or WiFi. For mobile phone users, BP data are transmitted to the system via the internet through NFC or Bluetooth. For most devices, an active data update is required to automatically transmit the data from the devices to the system. However, some recent devices with WiFi connections transfer data simultaneously and automatically to the system. Self-reported data, manually inputted by a user, are also accepted. All BP data from users who voluntarily register with this service are sent to the computer server at OMRON Healthcare Co., Ltd.

The WellnessLINK system provides various kinds of health support programmes, including antihypertensive healthcare, metabolic syndrome prevention, dietary improvement, and physical activity. In the antihypertensive healthcare programme, WellnessLINK provides personalised daily, weekly, monthly and yearly graphs of BP, which are useful for individual BP management. In addition, feedback is provided to the users after accumulated data are analysed.

### Blood pressure data analysis

A total of 2 164 321 individuals in Japan registered with the WellnessLINK system as of the end of September 2015. Among these users, 110 454 sent BP data to the system automatically from BP measurement devices, or manually. Self-reported data that were manually inputted by users were excluded from the data analysis in this study. In this report, we analysed 41.7 million BP measurement data from 64 536 registered users (51 335 men, 13 201 women; mean age 52.9 years) who sent their measured BP data automatically. Among these users, 24% were treated with antihypertensive medication.

BP values measured at home between 04:00 and 09:59 were defined as ‘morning BP’, while those measured at home between 19:00 and 01:59 were defined as ‘evening BP’. Individual mean morning BP values were calculated using the first measured BP value in the morning, and those measured during the following 10 minutes. Individual mean evening BP values were calculated using the last measured BP value in the evening, and the values measured during the previous 10 minutes. The monthly mean home BP values were calculated by averaging individuals’ home BP values for all participants.

Descriptive statistical values such as mean monthly systolic BP (SBP) and diastolic BP (DBP) (and standard errors) were calculated by sex and age group for 2 years, from September 2013 to September 2015. Monthly mean BP changes for 2 years are shown in figures along with mean outdoor temperatures. Inter-month differences in BP were assessed using analysis of variance. The t test for unpaired observations was used to determine the significance of the difference, after Bonferroni correction for multiple comparisons. Multiple regression analysis was preformed to assess whether the association between temperature and BP is independent of age (continuous variable), gender (dichotomous; men: 1, women: 0) and geological location (Northern Japan/Eastern Japan/Central Japan/Kansai region/Western Japan). Four dummy variables for five geological locations were assigned and Eastern Japan was defined as the reference area. All P values were two sided, and P values < 0.05 were considered to be statistically significant. Mean outdoor temperatures were calculated using outdoor temperatures at the time of each measurement in the capital city of the prefecture in which each participant lived, obtained from the database of the Japan Meteorological Agency.

The participants of this study had voluntarily filled out the online consent form, agreed to send their data to the server managed by OMRON Healthcare Co., Ltd. using the WellnessLINK system service, and agreed with the condition that the data obtained by the WellnessLINK system would be analysed for medical research purposes after data anonymisation. The ethics committee of the Omron Healthcare Co., Ltd. approved the study protocol.

## Results

### Mean monthly morning and evening home SBP

**Figure 1:**
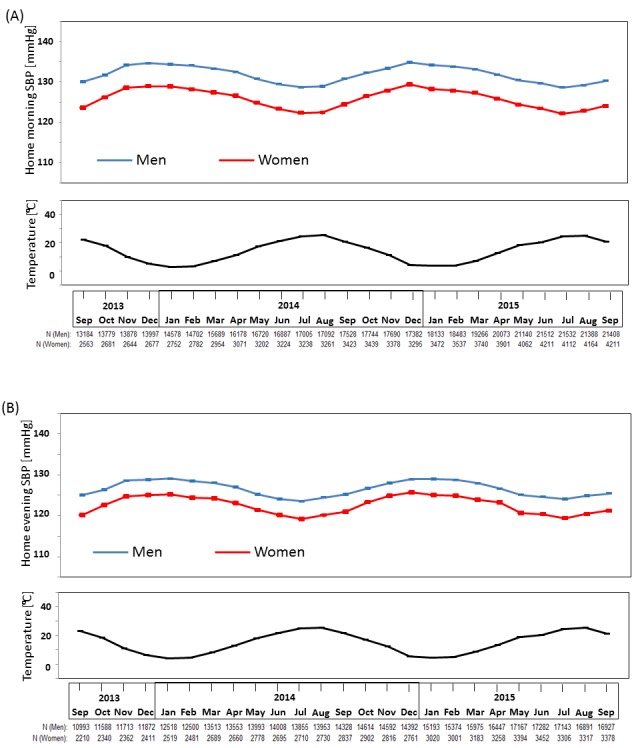
Monthly means (and standard errors) of (A) home morning and (B) home evening systolic blood pressure (SBP) from September 2013 to September 2015. The maximum winter–summer difference was larger for morning SBP (6.2 mmHg in men, 7.3 mmHg in women) than for evening SBP (5.5 mmHg in men, 6.5 mmHg in women).

**Figure 2:**
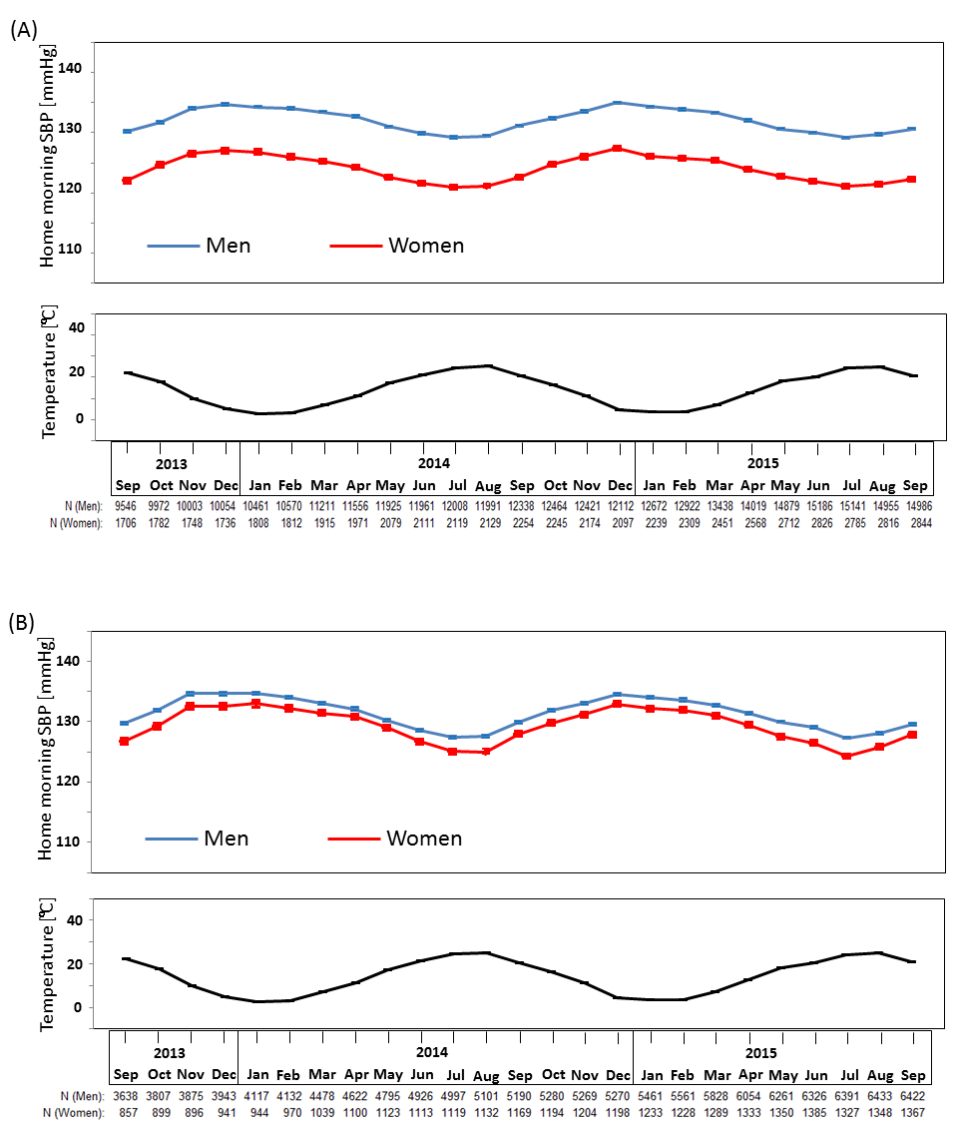
Monthly means (and standard errors) of home morning systolic blood pressure (SBP) according to age subgroups from September 2013 to September 2015. (A) Participants aged less than 60 years; (B) participants aged 60 years or older. The winter–summer difference was greater in older participants (7.3 mmHg in men, 8.7 mmHg in women) compared with younger participants (5.8 mmHg in men, 6.5 mmHg in women).

A total of 64 536 Japanese registered users automatically sent their measured BP data obtained nationwide from September 2013 to September 2015 (online supplementary table 1). Approximately 83% of the study participants were men, their mean age was 54 years old, and the treatment rate among people with hypertension in the present study was approximately 39%; these figures remained relatively stable throughout the study period. The mean monthly morning and evening home SBP, and mean monthly temperatures for a 2-year period are shown in figure 1. Seasonal variation was apparent in both morning and evening home SBP in both sexes; values were higher in the winter and lower in the summer (figure 1, online supplementary tables 2,3, supplementary movie). The relationship between home SBP and temperature showed a significant inverse association which was independent of age, gender and geological location (table 1). Age, gender and geographic location had no significant interaction on the relationship between BP and temperature (all P>0.05). The maximum winter–summer difference was a little greater for morning SBP (6.2 mmHg in men, 7.3 mmHg in women) compared with evening SBP (5.5 mmHg in men, 6.5 mmHg in women). A similar seasonal variation was observed in morning SBP for younger and older individuals (figure 2, online supplementary tables 4,5), with the winter–summer difference being a little greater in older (7.3 mmHg in men, 8.7 mmHg in women) compared with younger individuals (5.8 mmHg in men, 6.5 mmHg in women).

10.1136/bmjopen-2017-017351.supp1 Supplementary file 1

10.1136/bmjopen-2017-017351.supp2 Supplementary file 2

**Table 1:** Regression coefficients for the relationship between home morning systolic blood pressure with age, gender, temperature and geographic locations using multiple regression analysis weighted by numbers of participants in each month

	Regression coefficient	P value
Age (years)	0.274	0.02
Gender (men vs women)	6.05	<0.001
Temperature (°C)	−0.253	<0.001
Geological location*		
Northern Japan*	−0.395	0.054
Central Japan*	0.186	0.37
Kansai region*	−0.382	0.08
Western Japan*	−5.68	<0.001
Adjusted R: 0.9309		

*Versus Eastern Japan (Kanto region).

The amplitude of winter–summer difference was smaller for DBP compared with SBP; however, a similar trend of seasonal variation was observed in DBP (online supplementary tables 2–5).

### Monthly morning home SBP and mean outdoor temperatures

**Figure 3:**
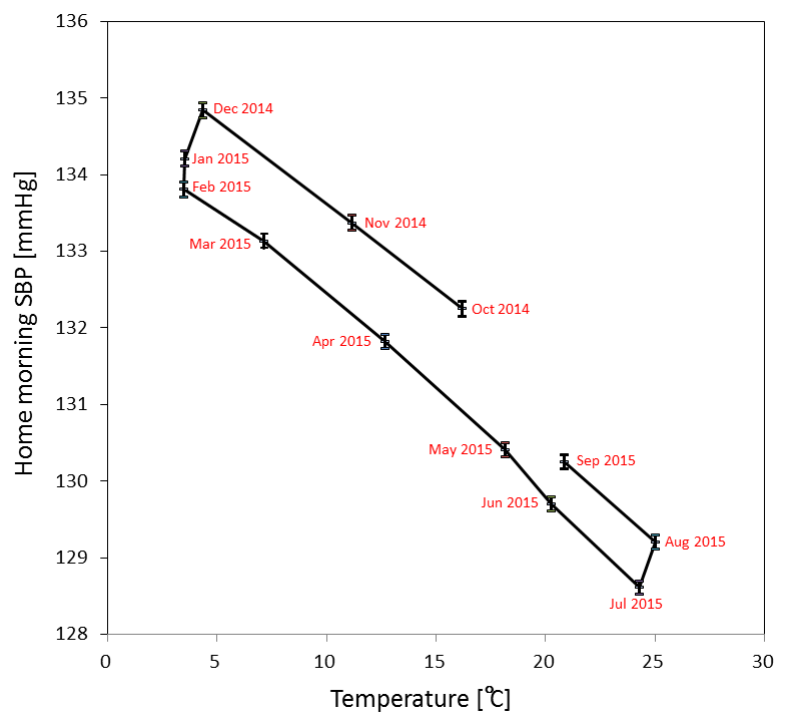
Association between monthly mean home morning systolic blood pressure (SBP) (and standard errors) and mean temperatures in each month from October 2014 to September 2015 in men. Home morning SBP was highest in winter and lowest in summer. Home morning SBP was higher in autumn than in spring, although outdoor temperatures were similar.

The association between mean monthly morning home SBP and mean outdoor temperatures for a 1-year period in men was skewed and showed a hysteresis loop characteristic (figure 3 and online supplementary table 2). Outside temperature was lowest in February (3.5°C) but similar from December (4.4°C) to February; however, morning home SBP lowered towards February (from 134.8 mmHg to 133.8 mmHg) from the highest recorded in December. Furthermore, outside temperature was highest in August (25.1°C) but similar from July (24.3°C) to August; however, morning home SBP was higher in August (129.2 mmHg) than the lowest recorded in July (128.6 mmHg). Morning home SBP from February to July was approximately 1.5 mmHg lower than the value from August to December (eg, morning home SBP was 1.5 mmHg higher in November (11.2°C) than in April (12.7°C)). There were statistical significances observed in BP between summer and winter months; however, there were a few inter-month combinations which did not reach statistical significance (March vs November, and May vs September). Similar findings were observed in evening home SBP and DBP, regardless of gender and age subgroups (online supplementary tables 2–5).

## Discussion

The association between BP and temperature was inverse (winter–summer difference: approximately 7 mmHg) and independent of other factors such as age, gender and geological locations. In multiple regression analysis, SBP fell by 0.27 mmHg for each 1°C increase.

Monitoring population-wide BP is important for public health, especially for CVD prevention.^1–6^ Previous studies have shown approximately 2–10 mmHg, 4–8 mmHg and 3 mmHg of seasonal variability in clinic BP, home BP and 24 hour ambulatory BP (ABP), respectively.^13–22^ Most clinics are temperature controlled, thus likely do not represent daily climate conditions.^15 23^ Seasonal variation in CVD incidence has been reported,^24–29^ and seasonal variation in BP has been suggested to be associated with seasonal variation in the incidence of CVD.^23^ Stamler *et al* estimated a reduction in population BP of 2–5 mmHg would deliver a reduction in mortality from stroke, mortality from coronary heart disease and total mortality of approximately 6–14%, 4–9% and 3–7%, respectively.^30^ However, there was no system available for public health authorities to detect population-wide BP to plan countermeasures, and announce alerts for CVD prevention. Thus, acquiring nationwide ‘real-world’ home BP data is desirable.

The rapid diffusion of home BP monitoring has been aided by technological progress, the wider availability of measurement devices, increased awareness of the importance of regular BP monitoring, and recognition of the usefulness of home BP measurement by international hypertension management guidelines.^4–9^ The National Health and Nutrition Survey of Japan showed that in 2010, 46% of adults and 72% of patients with hypertension had measured their BP at home within the previous year.^31^ Therefore, devices for home BP measurement are widely spread nationwide, and have been used more frequently in Japan than in any other country in the world. However, conventional data collection methods using self-recorded home BP may result in bias, mainly under-reported BP values.^7 32^ The uniqueness of the system in the present study is that nationwide home BP data are automatically collected and simultaneously transmitted electronically to the web-based home BP monitoring platform. This system enables the elimination of manual processes performed by clinical staff with substantial effort, and minimises the possibility of a selection bias in the data collection. Thus, we demonstrated the practical use of a web-based healthcare platform which may reliably reflect the trend of population-wide BP as ‘real-world’ data in weekly and monthly analyses.

Findings from other previous studies suggest that outdoor temperature and home central heating affect the seasonal variability of BP, especially during the winter.^16 22 23^ In the present study, a parallel association was observed between seasonal variation in home BP (about 7 mmHg) and changes in outdoor temperature in these ’real-world’ data. This finding is consistent with a previous report using a smaller sample size, and less frequent, manually collected data.^21^ One interesting finding was that home BP tended to be higher by about 1.5 mmHg during the autumn (summer to winter) than in the spring (winter to summer). To the best of our knowledge, this study is the first to report this hysteresis loop characteristic. Tighter BP control may be required during autumn than in spring.

Ideally, public health authorities would be able to note population-wide changes in BP by monitoring population-wide ‘real-world’ home BP data using this platform, and this would allow them to forecast CVD risk at appropriate times. Furthermore, public health authorities may be able to notify clinicians and practitioners to be cautious of over and under dosing antihypertensive medication by referencing the population-wide ‘real-world’ BP data which can be easily screened for appropriate BP control. Such new approaches may initiate new phases for CVD prevention and public health promotion. In addition to devices for home BP measurement, adding varieties of lifestyle monitoring devices related to BP management (eg, monitors for body weight, physical activity,^33^ sleep,^34^ body temperature, and urinary sodium to potassium ratio^35^) to this platform may help medical and public health experts to identify new realities, assess the concurrent population-wide CVD risk and plan appropriate countermeasures. Therefore, using this platform, appropriate feedback from public health authorities could support individuals to take the right action towards a healthy lifestyle.

To the best of our knowledge, this is the first study to report seasonal variation from a large sample of ‘real-world’ home BP data from non-medical settings. In this study, the participants were registered to use this web-based healthcare platform; the participants might have higher socioeconomic status since they may have higher computer literacy and be more health conscious compared with others in the general population. Therefore, seasonal variation in the ‘real-world’ home BP might have been underestimated due to the higher socioeconomic status of the study participants. Moreover, the participants in this web-based healthcare platform are free to register and leave at any time; this may aggravate the selection bias. However, the large sample size (>60 000), voluntary participation in the healthcare platform, measurement by a diverse range of individuals, various measurement times, including weekends, and non-clinical measurement settings are thought to minimise the possibility of selection bias in this study, and reflect casual BP values in the real world.

This study has some limitations. As the data obtained during the study were ‘real-world’ data, the room temperature during home BP measurements was unknown, some of the BP measurement devices might have been used by multiple users without differentiating the user identification numbers, and the frequency and timing of BP measurements may have differed for each individual.

Based on the findings in this study, combining current public health policies with emerging technologies may help authorities to monitor health-related indexes. To reduce CVD incidents, and for CVD prevention, we need to analyse the situation of individuals, and the entire population. Therefore, establishing a web-based healthcare platform might provide public health authorities with an alternative for active control of public health outcome. Initiation of a new phase for both CVD prevention and public health promotion may contribute to reducing medical expenditure nationwide.

## Supplementary Material

Reviewer comments

Author's manuscript
