# Supplementary material for: Seasonal variation in home blood pressure: findings from nationwide web-based monitoring in Japan
Source: BMJ Open. 2018 Jan 5;8(1):e017351. doi: 10.1136/bmjopen-2017-017351 (PMC5780696; doi:10.1136/bmjopen-2017-017351)
Supplement: Supplementary file 1 [file bmjopen-2017-017351supp001.pdf]

Supplementary Table 1. Characteristics of study participants, free living Japanese volunteers, from September, 2013 to September 2015.

Locations (Prefectures)		Men		Women	
		N	Mean age	N	Mean age
Nationwide		51335	51.4	13201	51.0
Northern Japan (Hokkaido, Tohoku regions)	Hokkaido	1471	50.6	430	50.4
	Aomori	311	48.2	74	50.8
	Iwate	392	51.9	61	53.3
	Miyagi	846	50.3	266	46.4
	Akita	246	50.7	43	54.5
	Yamagata	286	50.2	56	54.8
	Fukushima	566	52.2	182	56.8
Eastern Japan (Kanto region)	Ibaraki	1161	50.9	277	50.2
	Tochigi	753	49.5	156	49.7
	Gunma	613	50.9	143	50.9
	Saitama	3422	51.3	852	49.9
	Chiba	3189	51.2	817	50.7
	Tokyo	9673	50.7	2526	50.2

	Kanagawa	6275	51.5	1430	51.0
Central Japan	Niigata	599	50.5	128	50.3
(Chubu region)	Toyama	302	51.2	72	50.2
	Ishikawa	351	55.6	70	49.9
	Fukui	179	51.5	44	51.8
	Yamanashi	243	53.0	50	56.1
	Nagano	701	51.9	131	51.7
	Gifu	591	51.8	139	51.9
	Shizuoka	1177	51.3	274	51.5
	Aichi	2866	51.5	705	51.2
	Mie	577	52.0	160	49.1
Kansai region	Shiga	561	51.4	118	50.1
(Kinki region)	Kyoto	1274	51.1	346	51.2
	Osaka	3194	51.8	944	50.8
	Hyogo	2066	51.9	581	51.3
	Nara	448	53.4	115	53.6
	Wakayama	217	52.1	68	54.1
Western Japan	Tottori	138	51.6	39	49.6

(Chugoku, Shikoku,	Shimane	155	51.8	47	51.2
Kyushu, Okinawa	Okayama	576	51.7	162	52.4
regions)	Hiroshima	993	52.4	210	51.2
	Yamaguchi	357	52.8	110	51.0
	Tokushima	187	50.4	62	48.5
	Kagawa	312	50.9	87	51.5
	Ehime	367	50.1	111	50.2
	Kouchi	182	54.5	60	54.5
	Fukuoka	1479	51.7	418	50.9
	Saga	175	52.4	44	46.5
	Nagasaki	309	51.2	87	52.7
	Kumamoto	397	50.5	111	49.2
	Oita	260	52.6	68	51.1
	Miyazaki	204	51.6	60	47.1
	Kagoshima	331	49.8	100	49.7
	Okinawa	363	47.0	167	45.7

Supplementary Table 2. Monthly means (and standard errors) of home morning blood pressure from September 2013 to September 2015.

		Men					Women					Temp
		N	Systolic BP [mmHg]		Diastolic BP [mmHg]		N	Systolic BP [mmHg]		Diastolic BP [mmHg]		[C]
			Mean	SE	Mean	SE		Mean	SE	Mean	SE	
2013	Sep	13184	130.1	0.12	82.9	0.09	2563	123.6	0.31	76.4	0.22	22.1
	Oct	13779	131.7	0.12	83.9	0.09	2681	126.2	0.31	78.2	0.22	17.7
	Nov	13878	134.2	0.12	85.3	0.09	2644	128.6	0.31	79.6	0.22	10.0
	Dec	13997	134.6	0.12	85.5	0.09	2677	129.0	0.30	79.8	0.21	5.1
2014	Jan	14578	134.4	0.11	85.2	0.09	2752	128.9	0.31	79.7	0.21	2.7

Feb	14702	134.1	0.11	85.0	0.09	2782	128.2	0.30	79.3	0.21	3.1
Mar	15689	133.3	0.11	84.6	0.09	2954	127.4	0.28	78.7	0.20	7.0
Apr	16178	132.5	0.11	84.2	0.08	3071	126.6	0.28	78.2	0.20	11.4
May	16720	130.8	0.10	83.3	0.08	3202	124.8	0.27	77.2	0.19	17.2
Jun	16887	129.5	0.10	82.4	0.08	3224	123.3	0.26	76.0	0.19	21.2
Jul	17005	128.7	0.10	82.0	0.08	3238	122.3	0.26	75.4	0.20	24.4
Aug	17092	128.9	0.10	82.1	0.08	3261	122.4	0.26	75.5	0.20	25.3
Sep	17528	130.8	0.10	83.5	0.08	3423	124.4	0.26	77.0	0.19	20.7
Oct	17744	132.2	0.10	84.4	0.08	3439	126.5	0.26	78.4	0.19	16.2
Nov	17690	133.4	0.10	85.0	0.08	3378	127.9	0.27	79.3	0.19	11.2
Dec	17382	134.8	0.10	85.7	0.08	3295	129.4	0.28	80.0	0.19	4.4

2015	Jan	18133	134.2	0.10	85.3	0.08	3472	128.2	0.27	79.3	0.18	3.6
	Feb	18483	133.8	0.10	85.1	0.08	3537	127.9	0.26	79.3	0.18	3.5
	Mar	19266	133.1	0.10	84.8	0.08	3740	127.3	0.26	79.2	0.18	7.2
	Apr	20073	131.8	0.09	84.2	0.07	3901	125.9	0.25	78.4	0.17	12.7
	May	21140	130.4	0.09	83.4	0.07	4062	124.4	0.24	77.5	0.17	18.2
	Jun	21512	129.7	0.09	83.1	0.07	4211	123.5	0.23	77.1	0.17	20.3
	Jul	21532	128.6	0.09	82.5	0.07	4112	122.1	0.23	76.2	0.18	24.3
	Aug	21388	129.2	0.09	82.8	0.07	4164	122.8	0.24	76.6	0.17	25.1
	Sep	21408	130.3	0.09	83.7	0.07	4211	124.1	0.24	77.7	0.17	20.9

BP: blood pressure, C: degree Celsius, SE: Standard error, Temp: temperature, Jan: January, Feb: February, Mar: March, Apr: April, Jun: June, Jul: July, Aug: August, Sep: September, Oct: October, Nov: November, Dec: December

Supplementary Table 3. Monthly means (and standard errors) of home evening blood pressure from September 2013 to September 2015.

		Men					Women					Temp
		N	Systolic BP [mmHg]		Diastolic BP [mmHg]		N	Systolic BP [mmHg]		Diastolic BP [mmHg]		[C]
			Mean	SE	Mean	SE		Mean	SE	Mean	SE	
2013	Sep	10993	125.1	0.14	77.2	0.11	2210	120.2	0.34	72.6	0.24	22.8
	Oct	11588	126.4	0.13	78.2	0.11	2340	122.6	0.34	74.5	0.24	18.3
	Nov	11713	128.6	0.14	79.6	0.11	2362	124.7	0.33	76.0	0.24	11.1
	Dec	11872	128.8	0.14	79.6	0.11	2411	125.0	0.33	75.9	0.23	6.3
2014	Jan	12518	129.1	0.13	79.8	0.10	2519	125.2	0.32	76.0	0.23	4.1

Feb	12500	128.5	0.13	79.3	0.10	2481	124.5	0.32	75.6	0.23	4.6
Mar	13513	128.1	0.12	79.1	0.10	2689	124.3	0.31	75.3	0.22	8.4
Apr	13553	127.0	0.12	78.5	0.10	2660	123.1	0.31	74.5	0.22	12.8
May	13993	125.2	0.12	77.3	0.10	2778	121.4	0.30	73.2	0.22	17.7
Jun	14008	124.1	0.12	76.5	0.10	2695	120.2	0.30	72.1	0.21	21.5
Jul	13855	123.6	0.12	76.1	0.10	2710	119.3	0.30	71.7	0.22	24.9
Aug	13953	124.5	0.12	76.7	0.10	2730	120.1	0.30	72.1	0.22	25.4
Sep	14328	125.3	0.12	77.5	0.10	2837	121.0	0.30	73.2	0.22	21.6
Oct	14614	126.7	0.12	78.5	0.09	2902	123.4	0.30	75.0	0.21	17.2
Nov	14592	128.0	0.12	79.4	0.10	2816	125.0	0.32	76.0	0.22	12.4
Dec	14392	128.9	0.12	79.7	0.10	2761	125.8	0.32	76.4	0.23	5.2

2015	Jan	15193	129.0	0.12	79.7	0.09	3020	125.1	0.30	75.9	0.21	4.5
	Feb	15374	128.7	0.12	79.7	0.09	3001	124.9	0.30	76.0	0.21	4.8
	Mar	15975	128.0	0.11	79.3	0.09	3183	123.9	0.29	75.6	0.21	8.6
	Apr	16447	126.7	0.11	78.5	0.09	3258	123.3	0.29	75.2	0.20	13.4
	May	17167	125.1	0.11	77.5	0.09	3394	120.7	0.27	73.5	0.19	18.7
	Jun	17282	124.7	0.11	77.4	0.09	3452	120.4	0.27	73.4	0.20	20.5
	Jul	17143	124.1	0.11	76.9	0.09	3306	119.4	0.27	72.8	0.21	24.6
	Aug	16891	124.9	0.11	77.5	0.09	3317	120.4	0.27	73.3	0.20	25.4
	Sep	16927	125.4	0.11	78.1	0.09	3378	121.3	0.27	74.1	0.20	21.3

BP: blood pressure, C: degree Celsius, SE: Standard error, Temp: temperature, Jan: January, Feb: February, Mar: March, Apr: April, Jun: June, Jul: July, Aug: August, Sep: September, Oct: October, Nov: November, Dec: December

Supplementary Table 4. Monthly means (and standard errors) of home morning blood pressure by age groups (ages less than 60 years) from September 2013 to September 2015.

		Men					Women					Temp
		N	Systolic BP [mmHg]		Diastolic BP [mmHg]		N	Systolic BP [mmHg]		Diastolic BP [mmHg]		[C]
			Mean	SE	Mean	SE		Mean	SE	Mean	SE	
2013	Sep	9546	130.2	0.14	84.7	0.11	1706	122.1	0.39	77.5	0.29	22.1
	Oct	9972	131.7	0.14	85.7	0.11	1782	124.7	0.39	79.4	0.28	17.7
	Nov	10003	134.0	0.14	87.1	0.11	1748	126.5	0.38	80.6	0.28	10.0
	Dec	10054	134.6	0.14	87.3	0.11	1736	127.0	0.38	81.0	0.28	5.1
2014	Jan	10461	134.3	0.14	87.0	0.10	1808	126.8	0.37	80.9	0.28	2.7

Feb	10570	134.1	0.14	86.8	0.10	1812	126.0	0.37	80.4	0.28	3.1
Mar	11211	133.4	0.13	86.5	0.10	1915	125.2	0.35	79.7	0.26	7.0
Apr	11556	132.7	0.13	86.1	0.10	1971	124.2	0.34	79.3	0.26	11.3
May	11925	131.0	0.12	85.2	0.10	2079	122.6	0.33	78.2	0.25	17.2
Jun	11961	129.8	0.13	84.5	0.10	2111	121.6	0.33	77.3	0.25	21.2
Jul	12008	129.2	0.12	84.1	0.10	2119	120.9	0.34	76.7	0.26	24.4
Aug	11991	129.5	0.12	84.3	0.10	2129	121.0	0.34	76.9	0.26	25.3
Sep	12338	131.1	0.13	85.6	0.10	2254	122.6	0.33	78.2	0.25	20.7
Oct	12464	132.4	0.12	86.4	0.09	2245	124.8	0.33	79.7	0.25	16.2
Nov	12421	133.5	0.12	87.0	0.09	2174	126.0	0.34	80.6	0.26	11.2
Dec	12112	135.0	0.13	87.8	0.10	2097	127.4	0.35	81.3	0.26	4.4

2015	Jan	12672	134.3	0.12	87.2	0.09	2239	126.1	0.34	80.4	0.24	3.6
	Feb	12922	133.9	0.12	87.0	0.09	2309	125.7	0.33	80.2	0.24	3.5
	Mar	13438	133.3	0.12	86.8	0.09	2451	125.4	0.32	80.3	0.23	7.2
	Apr	14019	132.0	0.11	86.1	0.09	2568	124.0	0.32	79.5	0.23	12.7
	May	14879	130.6	0.11	85.3	0.08	2712	122.8	0.30	78.8	0.22	18.2
	Jun	15186	130.0	0.11	85.0	0.08	2826	122.0	0.29	78.5	0.22	20.3
	Jul	15141	129.2	0.11	84.5	0.08	2785	121.1	0.30	77.8	0.23	24.3
	Aug	14955	129.7	0.11	84.9	0.08	2816	121.4	0.30	77.9	0.22	25.1
	Sep	14986	130.6	0.11	85.8	0.08	2844	122.3	0.30	78.9	0.21	20.9

BP: blood pressure, C: degree Celsius, SE: Standard error, Temp: temperature, Jan: January, Feb: February, Mar: March, Apr: April, Jun: June, Jul: July, Aug: August, Sep: September, Oct: October, Nov: November, Dec: December

Supplementary Table 5. Monthly means (and standard errors) of home morning blood pressure by age groups (ages 60 years or older) from September 2013 to September 2015.

		Men					Women					Temp
		N	Systolic BP [mmHg]		Diastolic BP [mmHg]		N	Systolic BP [mmHg]		Diastolic BP [mmHg]		[C]
			Mean	SE	Mean	SE		Mean	SE	Mean	SE	
2013	Sep	3638	129.7	0.22	78.0	0.15	857	126.7	0.49	74.2	0.31	22.1
	Oct	3807	131.9	0.22	79.3	0.15	899	129.3	0.48	75.9	0.31	17.7
	Nov	3875	134.6	0.22	80.7	0.15	896	132.6	0.50	77.6	0.32	10.0
	Dec	3943	134.6	0.22	80.8	0.15	941	132.5	0.47	77.6	0.30	5.0
2014	Jan	4117	134.7	0.21	80.8	0.15	944	133.0	0.50	77.5	0.31	2.8

Feb	4132	134.1	0.21	80.4	0.15	970	132.3	0.49	77.4	0.31	3.1
Mar	4478	133.1	0.20	80.0	0.14	1039	131.4	0.43	77.0	0.29	7.1
Apr	4622	132.1	0.19	79.5	0.14	1100	130.8	0.45	76.5	0.28	11.4
May	4795	130.2	0.18	78.5	0.13	1123	129.0	0.43	75.2	0.27	17.1
Jun	4926	128.5	0.18	77.5	0.13	1113	126.7	0.41	73.7	0.26	21.2
Jul	4997	127.4	0.18	76.8	0.13	1119	125.1	0.40	72.9	0.27	24.5
Aug	5101	127.6	0.18	77.0	0.13	1132	125.0	0.41	72.9	0.27	25.3
Sep	5190	129.9	0.18	78.5	0.13	1169	127.9	0.43	74.6	0.27	20.7
Oct	5280	131.9	0.18	79.7	0.13	1194	129.8	0.42	76.0	0.26	16.3
Nov	5269	133.0	0.18	80.3	0.13	1204	131.2	0.42	77.0	0.27	11.2
Dec	5270	134.6	0.18	81.0	0.13	1198	132.9	0.44	77.8	0.27	4.3

2015	Jan	5461	134.1	0.18	80.8	0.12	1233	132.2	0.42	77.3	0.26	3.5
	Feb	5561	133.6	0.17	80.7	0.12	1228	131.9	0.43	77.4	0.27	3.5
	Mar	5828	132.7	0.17	80.2	0.12	1289	131.0	0.41	77.1	0.26	7.2
	Apr	6054	131.4	0.16	79.5	0.12	1333	129.4	0.39	76.2	0.25	12.7
	May	6261	130.0	0.16	78.8	0.12	1350	127.5	0.37	74.9	0.24	18.1
	Jun	6326	129.0	0.16	78.4	0.11	1385	126.5	0.36	74.3	0.24	20.3
	Jul	6391	127.3	0.15	77.5	0.11	1327	124.3	0.37	73.0	0.25	24.3
	Aug	6433	128.0	0.15	77.8	0.11	1348	125.8	0.38	73.8	0.25	25.0
	Sep	6422	129.5	0.15	79.0	0.11	1367	127.9	0.38	75.3	0.25	20.9

BP: blood pressure, C: degree Celsius, SE: Standard error, Temp: temperature, Jan: January, Feb: February, Mar: March, Apr: April, Jun: June, Jul: July, Aug: August, Sep: September, Oct: October, Nov: November, Dec: December
