# Supplementary material for: Seasonal variation in home blood pressure: findings from nationwide web-based monitoring in Japan
Source: BMJ Open. 2018 Jan 5;8(1):e017351. doi: 10.1136/bmjopen-2017-017351 (PMC5780696; doi:10.1136/bmjopen-2017-017351)
Supplement: Author's manuscript [file bmjopen-2017-017351.draft_revisions.pdf]

BMJ Open is committed to open peer review. As part of this commitment we make the peer review history of every article we publish publicly available.

When an article is published we post the peer reviewers' comments and the authors' responses online. We also post the versions of the paper that were used during peer review. These are the versions that the peer review comments apply to.

The versions of the paper that follow are the versions that were submitted during the peer review process. They are not the versions of record or the final published versions. They should not be cited or distributed as the published version of this manuscript.

BMJ Open is an open access journal and the full, final, typeset and author-corrected version of record of the manuscript is available on our site with no access controls, subscription charges or pay-per-view fees (http://bmjopen.bmj.com).

If you have any questions on BMJ Open's open peer review process please email editorial.bmjopen@bmj.com

BMJ Open

Seasonal variation in home blood pressure as measured in a nationwide monitoring system

Journal:	*BMJ Open*
Manuscript ID	bmjopen-2017-017351
Article Type:	Research
Date Submitted by the Author:	19-Apr-2017
Complete List of Authors:	Iwahori, Toshiyuki; OMRON HEALTHCARE Co., Ltd, ; SHIGA UNIVERSITY of MEDICAL SCIENCE, HEALTH SCIENCE<br>Miura, katsuyuki; Shiga University of Medical Science, Health Science<br>Obayashi, Keiichi; Omron Kabushiki Kaisha<br>Ohkubo, Takayoshi<br>Nakajima, Hiroshi; Omron Kabushiki Kaisha<br>Shiga, Toshikazu; OMRON HEALTHCARE Co., Ltd.,<br>Ueshima, Hirotsugu; Shiga University of Medical Science, Health Science
**Primary Subject Heading**:	Public health
Secondary Subject Heading:	Public health, Cardiovascular medicine
Keywords:	PUBLIC HEALTH, Hypertension < CARDIOLOGY, EPIDEMIOLOGY

SCHOLARONE™ Manuscripts

For peer review only

Title:

Seasonal variation in home blood pressure as measured in a nationwide monitoring system

Authors:

Toshiyuki Iwahori, PhD [1,2*], Katsuyuki Miura, MD [1,3], Keiichi Obayashi, MSE [4], Takayoshi Ohkubo, MD [5], Hiroshi Nakajima, PhD [4], Toshikazu Shiga, PhD [2], Hirotsugu Ueshima, MD [1,3]

Affiliations:

[1] Department of Public Health, Shiga University of Medical Science, Otsu, Japan,

[2] OMRON Healthcare Co., Ltd., Muko, Japan,

[3] Center for Epidemiologic Research in Asia, Shiga University of Medical Science, Otsu, Japan,

[4] OMRON Corporation, Kizugawa, Japan,

[5] Department of Hygiene and Public Health, Teikyo University School of Medicine, Tokyo, Japan

For peer review only

Full address:

[1] Seta Tsukinowa-cho, Otsu, Shiga, 520-2192, Japan,

[2] 53, Kunotsubo, Terado-cho, Muko, Kyoto, 617-0002, Japan,

[3] Seta Tsukinowa-cho, Otsu, Shiga, 520-2192, Japan,

[4] 9-1 Kizugawadai, Kizugawa, Kyoto, 619-0283, Japan,

[5] 2-11-1 Kaga, Itabashi-ku, Tokyo, 173-8605, Japan

Corresponding author: Toshiyuki Iwahori, PhD

Department of Public Health

Shiga University of Medical Science

Seta Tsukinowa-cho, Otsu, Shiga 520 2192, Japan

Tel.: +81 77 548 2191

Fax: +81 77 543 9732

E-mail address: iwahori@belle.shiga-med.ac.jp

For peer review only

Abstract

Objectives: Our aim was to assess seasonal variation in home blood pressure (BP) among free-living nationwide users using home BP values accumulated from web-based health care platform established in Japan.

Settings: An observational study. OMRON Healthcare has been developing web-based personal health care record systems in Japan since November 2010; over two million voluntary participants had joined this platform in September 2015. Nationwide home BP measured by oscillometric-type electronic sphygmomanometers from over 110 thousand voluntary participants have been collected to the system from devices.

Participants: Seasonal variation in home BP was evaluated among 64,536 (51,335 men, 13,201 women; mean age, 52.9 years) free-living nationwide users whom automatically and simultaneously transmitted data to the system from devices.

Primary outcome measures: Mean monthly and weekly home BP

Results: Changes in BP were associated with seasonal changes in temperature. Highest and lowest BP was observed in December and July, respectively. Substantial seasonal differences in the mean values of morning and evening home systolic BP between summer and winter were 6.2 mmHg and 5.5 mmHg in men, and 7.3 mmHg and 6.5 mmHg in women. Seasonal variation was a little larger in older (7.3 mmHg in men, 8.7 mmHg in women) than in younger individuals (5.8 mmHg in men, 6.5 mmHg in

For peer review only

women). BP from February to July showed approximately 1.5 mmHg lower than the value from August to December.

Conclusions: Web-based healthcare platform has enabled easier monitoring of population-wide BP. Tighter BP control is necessary in winter than in summer, and especially in colder climate toward winter than toward summer. New technologies using web-based self-monitoring systems for health-related indexes are expected to initiate a new phase of cardiovascular disease prevention, and public health promotion.

(281/300 words)

For peer review only

Strengths and limitations of this study

- Frequent nationwide home BP monitoring by the web-based platform enabled to see seasonal variation of home BP in monthly and weekly changes in large sample (>60,000) from non-medical settings.
- Nationwide home BP data automatically collected and electronically transmitted simultaneously to the web-based home BP monitoring platform minimized the selection bias and reflected the casual BP values in the real world.

- The majority of the participants might have been health conscious individuals.
- Timing and frequency of the measurement, and the room temperature during home BP measurements remain unknown.

For peer review only

Introduction

The management of blood pressure (BP) is one of the most important strategies for the prevention, and management of cardiovascular diseases (CVD) around the world.[1–6] Although BP management has been generally based on BP values measured in the clinical setting by medical professionals, home BP monitoring by individuals is becoming increasingly important in diagnosis, and control of arterial hypertension.[4–8]

Electronic devices for home BP measurement were developed in the 1990s. Companies that manufacture devices for home BP measurement tend to concentrate in the East Asian region (Japan, South Korea, and Taiwan). Japan is unique for its high coverage of home BP measurement; nearly 20 million devices were estimated to exist out of approximately 50 million households by the sales record of BP monitors sold by OMRON Healthcare (Kyoto, Japan) in 2012.

Under these conditions, OMRON Healthcare, a health care device manufacturer, developed a web-based health care platform in 2010 that consumers can join voluntarily after purchasing one of its products. OMRON Healthcare's recently developed devices for home BP measurement send all measured values to a web-based database electronically via personal computers, or mobile phones. From 2010 to 2015, more than two million people in Japan have voluntarily participated in this web-based health care platform. Nowadays, these newly established technologies and platforms show

For peer review only

possibilities of lowering the hurdles to detect useful population-wide information and new approach for public health authorities.

Multiple observational studies have examined the seasonal variation of BP in clinical settings;[9] however, there is no report on seasonal variation using nationwide large sample of home BP monitoring data from non-medical settings. Thus, our aims were to assess seasonal variation in home blood pressure among free-living nationwide users by data collection by this web-based health care platform, and to see the applications for public health policy making.

Materials and Methods

Web-based personal healthcare record systems

OMRON Healthcare has been developing web-based personal health care record systems. One of its application services is named as WellnessLINK, which was deployed on November 1st, 2010. WellnessLINK utilizes many kinds of health care devices connected to the internet via personal computers, and mobile phones to collect various types of healthcare data into a database. The devices currently employed (but still expanding) by the services are as follows: a home BP monitor; a body weight, and body composition monitor; a physical activity monitor (pedometer, and calorie meter); a

For peer review only

sleep monitor; and a body temperature monitor. The services provided are for various health care items including diet, physical activity, sleep, antihypertensive health care, metabolic syndrome prevention, and women's health care.

The WellnessLINK system records BP values measured at home using oscillometric-type electronic sphygmomanometers, including an upper arm BP monitor (HEM-7510C, HEM-7500F, HEM-7324C, HEM-7320F, HEM-7280C, HEM-7270C, HEM-7250-IT; OMRON Healthcare), a spot arm BP monitor (HEM-1025; OMRON Healthcare), and a wrist BP monitor (HEM-6321T, HEM-6320T, HEM-6310F, HEM-6300F; OMRON Healthcare). The users receive an instruction manual for BP measurement based on the Japanese Society of Hypertension Guidelines for Self-Monitoring of Blood Pressure at Home.[7] Users then access the website via their personal computer, or mobile phone, and register after approving a privacy policy agreement. For personal computer users, BP data are transmitted to the system via internet by connecting a sphygmomanometer using either by Universal Serial Bus (USB), Near Field Communication (NFC), Bluetooth, or WiFi. For mobile phone users, BP data are transmitted to the system via the internet through either NFC, or Bluetooth. In most of the devices, an active data update is required to transmit the data automatically from the devices to the system by the individuals. However, some recent

For peer review only

devices with WiFi connections transfer data both simultaneously, and automatically to the system. Self-reported data, manually inputted by a user, is also accepted. All BP data from users who voluntarily register with this service are sent to the computer server at OMRON Healthcare.

The WellnessLINK system provides various kinds of health support programs, including antihypertensive health care, metabolic syndrome prevention, dietary improvement, and physical activity. In the antihypertensive health care program, WellnessLINK provides personalized daily, weekly, monthly, and yearly graphs of BP, which are useful for individual BP management. In addition, feedback is provided to the users after accumulated data is analyzed.

Blood pressure data analysis

A total of 2,164,321 individuals in Japan registered in the WellnessLINK system by the end of September 2015. Among these users, 110,454 sent BP data to the system automatically from BP measurement devices, or manually. Self-reported data that were manually inputted by users were excluded from the data analysis in this study. In this report, we analyzed 41.7 million BP measurement data from 64,536 registered users (51,335 men, 13,201 women; mean age, 52.9 years) who sent their all measured BP data

For peer review only

automatically. Among these users, 24% were treated with anti-hypertensive medication.

BP values measured at home between 4:00 AM, and 9:59 AM were defined as "morning BP," while those measured at home between 7:00 PM, and 1:59 AM were defined as "evening BP." Individual mean morning BP values were calculated using the first measured BP value in the morning, and measured within ten minutes later. Individuals mean evening BP values were calculated using the last measured BP value in the evening, and values measured within ten minutes previously.

Descriptive statistics values such as mean monthly systolic and diastolic BPs (and standard errors) were calculated by sex, and age groups for two years, from September 2013 to September 2015. Monthly mean BP changes for two years were shown in figures along with mean outdoor temperatures. Mean outdoor temperatures were calculated using outdoor temperatures at the time of each measurement in the capital city of the prefecture in which each participant lived, referenced from the database of the Japan Meteorological Agency.

The participants of this study had voluntarily filled out the online consent form, agreed to send their data to the data server managed by OMRON Healthcare to use the WellnessLINK system service, and agreed with the condition that these data obtained by the WellnessLINK system would be analyzed for medical research use after data anonymization. The ethics committee of the Omron Healthcare Company approved the

For peer review only

study protocol.

Results

Mean monthly morning and evening home systolic blood pressure

A total of 64,536 Japanese registered users sent their all measured BP data automatically from September 2013 to September 2015 from nationwide (Supplementary Table 1). Mean monthly morning and evening home systolic BP (SBP), as well as mean monthly temperatures for a two-year period, are shown in Figure 1. Seasonal variation was apparent in both morning, and evening home SBP in both sexes; values were higher in the winter, and lower in the summer (Figure 1, Supplemental Table 2-3, Supplemental Movie). The maximum winter-summer difference was a little larger for morning SBP (6.2 mmHg in men, 7.3 mmHg in women) compared with evening SBP (5.5 mmHg in men, 6.5 mmHg in women). Similar seasonal variation was observed in morning SBP for younger, and older individuals (Figure 2, Supplemental Table 4-5), with the winter-summer difference being a little larger in older (7.3 mmHg in men, 8.7 mmHg in women) than in younger individuals (5.8 mmHg in men, 6.5 mmHg in women).

The amplitude of winter-summer difference was smaller in diastolic BP compared

For peer review only

to SBP; however, similar trend of seasonal variation was observed in diastolic BP (Supplemental Table 2-5).

Monthly morning home systolic blood pressure and mean outdoor temperatures

The association between mean monthly morning home SBP and mean outdoor temperatures for a 1-year period in men was skewed and showed hysteresis loop characteristic (Figure 3 and Supplemental Table 2). Outside temperature was lowest in February (3.5°C) but similar from December (4.4°C) to February; however, morning home SBP lowered toward February (134.8 mmHg to 133.8 mmHg) from the highest recorded in December. Furthermore, outside temperature was highest in August (25.1°C) but similar from July (24.3°C) to August; however, morning home SBP was higher in August (129.2 mmHg) than the lowest recorded in July (128.6 mmHg). Morning home SBP from February to July showed approximately 1.5mmHg lower than the value from August to December (e.g., morning home SBP was 1.5 mmHg higher in November [11.2°C] than in April [12.7°C]). Similar findings were observed in evening home SBP and DBP, regardless of gender and age subgroups (Supplemental Table 2-5).

Discussion

For peer review only

Monitoring population-wide BP is important for public health, especially for CVD prevention.[1-6] Previous studies have shown approximately 2–10 mmHg, 4–8 mmHg, and 3 mmHg of seasonal variability in clinic BP, home BP, and 24-hour ambulatory BP (ABP), respectively.[10-19] Most clinics are temperature controlled; thus, likely do not represent daily climate conditions.[12,20] Seasonal variation in CVD incidence has been reported,[21–26] and seasonal variation in BP has been suggested to be associated with seasonal variation in the incidence of CVD.[20] Stamler et al. estimated the reduction in population BP for 2-5 mmHg deliver reduction in mortality of stroke, mortality of coronary heart disease and total mortality for approximately 6-14%, 4-9% and 3-7%, respectively [27]. However, there was no system available for public health authorities to detect population-wide BP to plan out countermeasures, and announce alert for CVD prevention. Thus, acquiring nationwide home BP data is desirable.

The rapid diffusion of home BP monitoring has been aided by technological progress, the wider availability of measurement devices, increased awareness of the importance of regular BP monitoring, and recognition of the usefulness of home BP measurement by international hypertension management guidelines.[4–8] The National Health and Nutrition Survey of Japan showed that in 2010, 46% of adults, and 72% of hypertensive patients had measured their BP at home within the previous year.[28] Therefore, devices for home BP measurement have been spread nationwide, have been

used more frequently in Japan than in any other country in the world. However, conventional data collection methods using self-recorded home BP may result in a bias, mainly underreported BP values.[7,29] The uniqueness of the system in the present study is that nationwide home BP data are automatically collected, and electronically transmitted simultaneously to the web-based home BP monitoring platform. This system enables the elimination of manual processes performed with substantial effort by clinical staff, and minimizes the possibility of a selection bias in the data collection. Thus, we demonstrated the practical use of web-based healthcare platform which may reliably reflect the trend of population-wide BP here in weekly and monthly analyses.

Findings from other previous studies suggest that outdoor temperature, and home central heating affect the seasonal variability of BP, especially during the winter.[13,19-20] In the present study, a parallel association was observed between seasonal variation in home BP (about 7 mmHg) and changes in outdoor temperature. This finding was consistent with previous report with smaller sample sized, lower frequent and manually collected data.[18] One interesting finding was that home BP tended higher for about 1.5 mmHg during autumn (summer to winter) than in spring (winter to summer). To the best of our knowledge, we are the first to report this hysteresis loop characteristic. Tighter BP control may be required during autumn than in spring.

Ideally, public health authorities would be able to notice population-wide changes

For peer review only

in BP through monitoring population-wide home BP data by using this platform, and this would allow them to forecast CVD risk at appropriate times. Furthermore, public health authorities may also make announcements for clinicians, and practitioners to be cautious of over- and under-dose of anti-hypertensive medication by referencing the population-wide BP for appropriate BP control. Such new approaches may initiate new phases for CVD prevention and public health promotion. In addition to devices for home BP measurement, adding varieties of lifestyle monitoring devices related to BP management (e.g., monitors for body weight, physical activity [30], sleep [31], body temperature, and urinary sodium-to-potassium ratio [32]) to this platform may support medical and public health experts to identify new realities, assess the concurrent population-wide CVD risk and plan out appropriate countermeasures. Therefore, by using this platform, much appropriate feedbacks from public health authorities could be expected to support individuals to take right action toward healthy lifestyle.

To the best of our knowledge, this is the first study to report seasonal variation from a large sample of home BP data from non-medical settings. The large sample size (>60,000), voluntary participation in the healthcare platform, measurement by a diverse range of individuals, various measurement times, including weekends, and non-clinical measurement settings are thought to minimize the possibility of selection bias in this study, and reflect casual BP values in the real world. However, this study has some

For peer review only

limitation. Room temperature during home BP measurements was unknown, some of the BP measurement devices might have been used by multiple users, and the frequency and timing of BP measurements may have differed for each individual. The individuals in the present study were participants who voluntarily joined the web-based healthcare service established by private company; thus the majority of the participants might have been health conscious individuals.

Based on the findings in this study, combining current public health policies with emerging technologies could be expected to help authorities monitor health-related indexes. To reduce CVD incidents, and for CVD prevention, we need to piece out the situation of individuals of entire population. Therefore, establishing web-based healthcare platform by public health authorities might be an alternative for obtaining eyes for active control of public health outcome. Initiation of a new phase for both CVD prevention and public health promotion may contribute to reduce nationwide medical expenditures.

(2226/3000words)

For peer review only

References

1. Lewington S, Clarke R, Qizilbash N, Peto R, Collins R; Prospective Studies Collaboration. Age-specific relevance of usual blood pressure to vascular mortality: a meta-analysis of individual data for one million adults in 61 prospective studies. *Lancet*. 2002 Dec 14;**360**(9349):1903-13.

2. Xie X, Atkins E, Lv J, et al. Effects of intensive blood pressure lowering on cardiovascular and renal outcomes: updated systematic review and meta-analysis. *Lancet*. 2016 Jan 30;**387**(10017):435-43.

3. Jaffe MG, Young JD. The Kaiser Permanente Northern California Story: Improving Hypertension Control From 44% to 90% in 13 Years (2000 to 2013). *J Clin Hypertens (Greenwich)*. 2016 Apr;18(4):260-1.

4. Mancia G, Fagard R, Narkiewicz K, et al. 2013 ESH/ESC guidelines for the management of arterial hypertension: the Task Force for the Management of Arterial Hypertension of the European Society of Hypertension (ESH) and of the European Society of Cardiology (ESC). *Blood Press* 2013; **22**: 193–278.

5. Armstrong C. JNC8 guidelines for the management of hypertension in adults. *Am Fam Physician*. 2014 Oct 1;**90**(7):503-4.

6. Shimamoto K, Ando K, Fujita T, et al. The Japanese Society of Hypertension Guidelines for the Management of Hypertension (JSH 2014). *Hypertens Res.* 2014 Apr;**37**(4):253-390. doi: 10.1038/hr.2014.20.

7. Imai Y, Kario K, Shimada K, et al. The Japanese Society of Hypertension Guidelines for Self-monitoring of Blood Pressure at Home (Second Edition). *Hypertens Res*. 2012 Aug;**35**(8):777-95. doi: 10.1038/hr.2012.56.

8. Parati G, Stergiou GS, Asmar R, et al. European Society of Hypertension guidelines for blood pressure monitoring at home: a summary report of the Second International Consensus Conference on Home Blood Pressure Monitoring. *J Hypertens.* 2008 Aug;**26**(8):1505-26.

9. Hanazawa T, Asayama K, Watabe D, Hosaka M, Satoh M, Yasui D, Obara T, Inoue

For peer review only

R, Metoki H, Kikuya M, Imai Y, Ohkubo T. Seasonal variation in self-measured home blood pressure among patients on antihypertensive medications: HOMED-BP study. *Hypertens Res*. 2016 Sep 29. doi: 10.1038/hr.2016.133.

10. Rose G. Seasonal variation in blood pressure in man. *Nature.* 1961 Jan 21;**189**:235.

11. Brennan PJ, Greenberg G, Miall WE, Thompson SG. Seasonal variation in arterial blood pressure. *Br Med J (Clin Res Ed).* 1982 Oct 2;**285**(6346):919-23.

12. Madsen C, Nafstad P. Associations between environmental exposure and blood pressure among participants in the Oslo Health Study (HUBRO). *Eur J Epidemiol.* 2006;**21**(7):485-91.

13. Lewington S, Li L, Sherliker P, et al. Seasonal variation in blood pressure and its relationship with outdoor temperature in 10 diverse regions of China: the China Kadoorie Biobank. *J Hypertens.* 2012 Jul;**30**(7):1383-91.

14. Modesti PA, Morabito M, Bertolozzi I, et al. Weather-related changes in 24-hour blood pressure profile: effects of age and implications for hypertension management. *Hypertension*. 2006 Feb;**47**(2):155-61.

15. Barnett AG, Sans S, Salomaa V, Kuulasmaa K, Dobson AJ; WHO MONICA Project. The effect of temperature on systolic blood pressure. *Blood Press Monit.* 2007 Jun;**12**(3):195-203.

16. Sega R, Cesana G, Bombelli M, et al. Seasonal variations in home and ambulatory blood pressure in the PAMELA population. Pressione Arteriose Monitorate E Loro Associazioni. *J Hypertens.* 1998 Nov;**16**(11):1585-92.

17. Alpérovitch A, Lacombe JM, Hanon O, et al. Relationship between blood pressure and outdoor temperature in a large sample of elderly individuals: the Three-City study. *Arch Intern Med.* 2009 Jan 12;**169**(1):75-80.

18. Imai Y, Munakata M, Tsuji I, et al. Seasonal variation in blood pressure in normotensive women studied by home measurements. *Clin Sci (Lond).* 1996 Jan;**90**(1):55-60.

For peer review only

19. Murakami S, Otsuka K, Kono T, et al. Impact of outdoor temperature on prewaking morning surge and nocturnal decline in blood pressure in a Japanese population. *Hypertens Res.* 2011 Jan;**34**(1):70-3.

20. Cuspidi C, Ochoa JE, Parati G. Seasonal variations in blood pressure: a complex phenomenon. *J Hypertens.* 2012 Jul;**30**(7):1315-20.

21. Analitis A, Katsouyanni K, Biggeri A, et al. Effects of cold weather on mortality: results from 15 European cities within the PHEWE project. *Am J Epidemiol.* 2008 Dec 15;**168**(12):1397-408.

22. Danet S, Richard F, Montaye M, et al. Unhealthy effects of atmospheric temperature and pressure on the occurrence of myocardial infarction and coronary deaths. A 10-year survey: the Lille-World Health Organization MONICA project (Monitoring trends and determinants in cardiovascular disease). *Circulation.* 1999 Jul 6;**100**(1):E1-7.

23. van Rossum CT, Shipley MJ, Hemingway H, et al. Seasonal variation in cause-specific mortality: are there high-risk groups? 25-year follow-up of civil servants from the first Whitehall study. *Int J Epidemiol.* 2001 Oct;**30**(5):1109-16.

24. Shinkawa A, Ueda K, Hasuo Y, Kiyohara Y, Fujishima M. Seasonal variation in stroke incidence in Hisayama, Japan. *Stroke.* 1990 Sep;**21**(9):1262-7.

25. Sheth T, Nair C, Muller J, Yusuf S. Increased winter mortality from acute myocardial infarction and stroke: the effect of age. *J Am Coll Cardiol.* 1999 Jun;**33**(7):1916-9.

26. Turin TC, Kita Y, Rumana N, et al. Stroke case fatality shows seasonal variation regardless of risk factor status in a Japanese population: 15-year results from the Takashima Stroke Registry. *Neuroepidemiology*. 2009;**32**(1):53-60.

27. Stamler R. Implications of the INTERSALT study. *Hypertension*. 1991 Jan;17(1 Suppl):I16-20.

28. National Institute of Health and Nutrition. The National Health and Nutrition

For peer review only

Survey Japan, 2010. Dai-ichi Shuppan Co. Ltd.: Tokyo, Japan, 2013 (in Japanese).

29. Mengden T, Hernandez Medina RM, Beltran B, et al. Reliability of reporting self-measured blood pressure values by hypertensive patients. *Am J Hypertens.* 1998 Dec;**11**(12):1413-7.

30. Tanaka C, Reilly JJ, Tanaka M, Tanaka S. Seasonal changes in objectively measured sedentary behavior and physical activity in Japanese primary school children. *BMC Public Health*. 2016 Sep 13;16:969.

31. Hashizaki M, Nakajima H, Kume K. Monitoring of Weekly Sleep Pattern Variations at Home with a Contactless Biomotion Sensor. *Sensors (Basel)*. 2015 Aug 3;15(8):18950-64.

32. Iwahori T, Ueshima H, Ohgami N, et al. Effectiveness of a self-monitoring device for urinary sodium/potassium ratio on dietary improvement in free-living adults: a randomized controlled trial. *J Epidemiol*. 2017 (in press).

For peer review only

Supplementary Information

Five supplementary tables and one supplementary movie. Supplementary movie shows the nationwide distribution of weekly mean home morning systolic blood pressure from September 2013 to September 2015.

Acknowledgements

This study was conducted by OMRON Healthcare Co., Ltd., and OMRON Corporation in cooperation with the Department of Public Health, Shiga University of Medical Science.

Funding

The running cost of this observational study was funded by OMRON Healthcare Co. Ltd.

Author contributions

KM, TO, HN, TS, HU contributed to the design of the study; KO, HN participated in data collection and data analysis; KM, KO, TI, TO and HU contributed to manuscript drafting; all authors participated in critical revisions of the manuscript; all authors approved the final version of the manuscript for submission.

For peer review only

Declaration of interests

TI and TS are employee of OMRON Healthcare Co., Ltd. KO and HN are employee of OMRON Corporation. Dr. Hirotsugu Ueshima served as a consultant for this project. Dr. Katsuyuki Miura received a research grant from OMRON Healthcare Co., Ltd.

For peer review only

Figure legends

Figure 1. Monthly means (and standard errors) of (A) home morning and (B) home evening systolic blood pressure (SBP) from September 2013 to September 2015. The maximum winter-summer difference was larger for morning SBP (6.2 mmHg in men, 7.3 mmHg in women) than for evening SBP (5.5 mmHg in men, 6.5 mmHg in women).

Figure 2. Monthly means (and standard errors) of home morning SBP by age groups from September 2013 to September 2015; (A) Participants aged less than 60 years; (B) participants aged 60 years or older. The winter-summer difference was larger in older participants (7.3 mmHg in men, 8.7 mmHg in women) than in younger participants (5.8 mmHg in men, 6.5 mmHg in women).

Figure 3. Association between monthly mean home morning SBP (and standard errors) and mean temperatures in each month from October 2014 to September 2015 in men. Home morning SBP was highest in winter and lowest in summer. Home morning SBP was higher in autumn than in spring, although outdoor temperatures were similar.

For peer review only

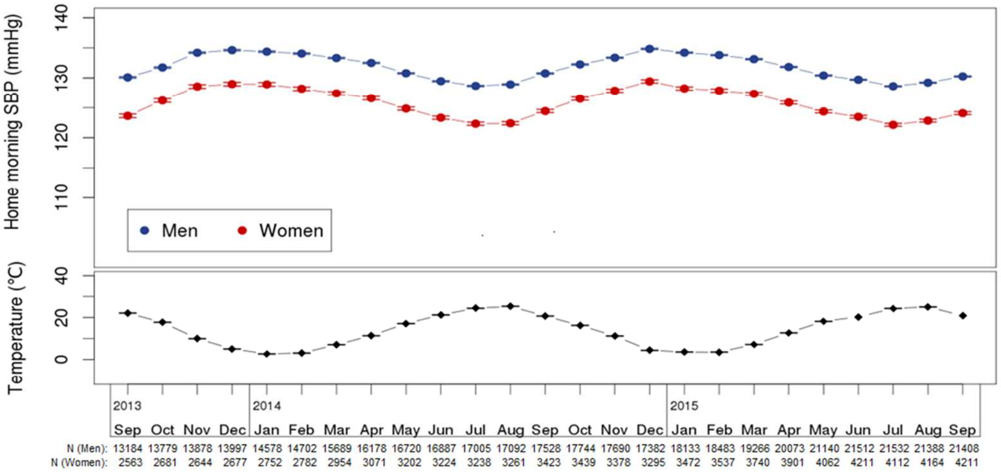

Figure 1A for Figure 1

265x124mm (96 x 96 DPI)

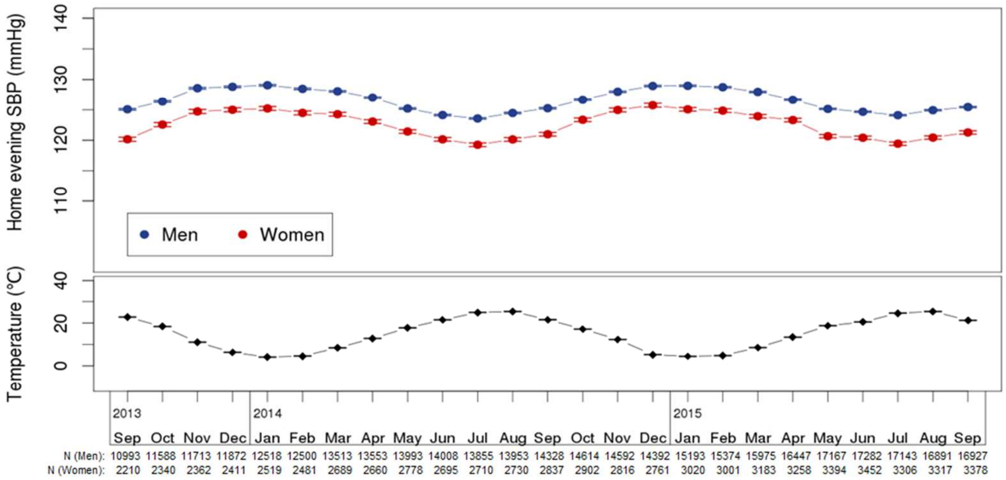

Figure 1B for Figure 1

262x125mm (96 x 96 DPI)

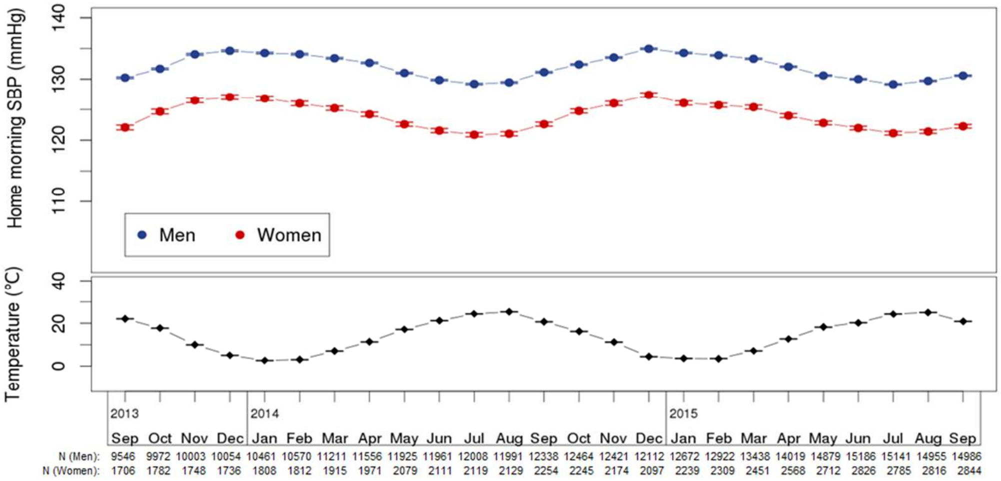

Figure 2A for Figure 2

262x125mm (96 x 96 DPI)

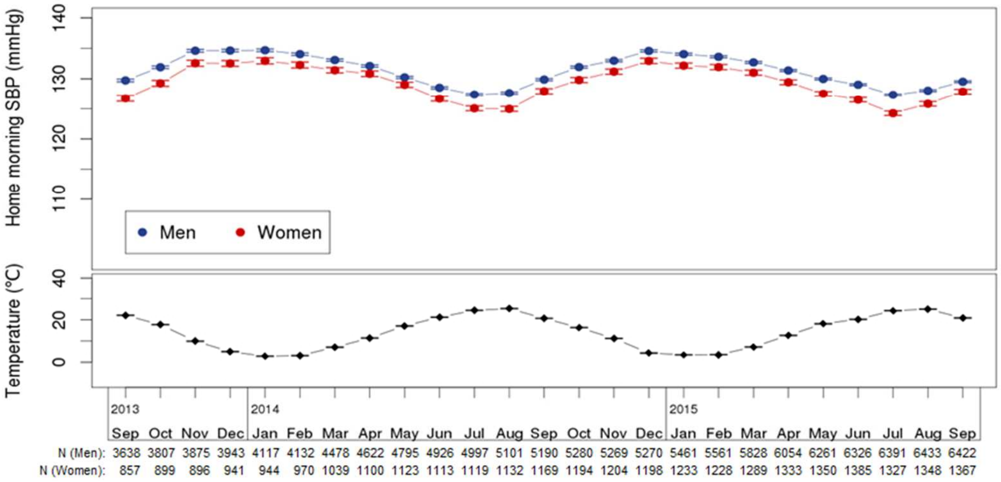

Figure 2B for Figure 2

228x109mm (96 x 96 DPI)

For peer review only

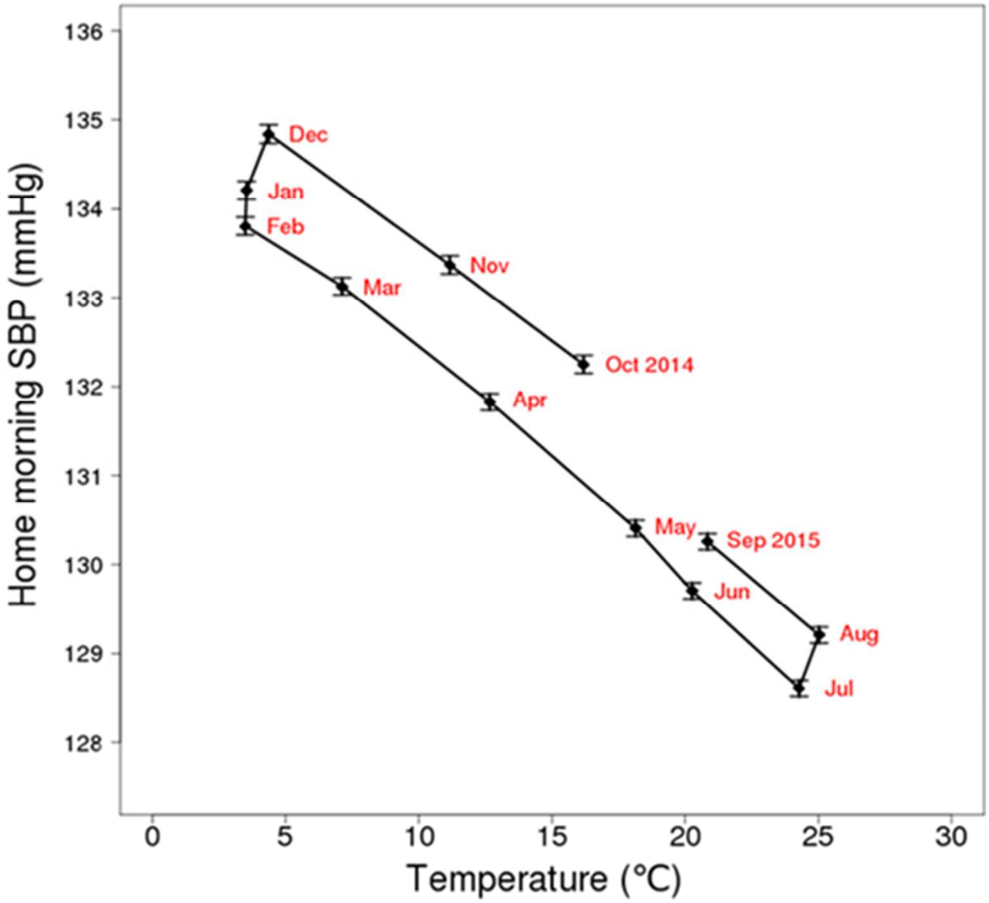

Figure 3

150x137mm (96 x 96 DPI)

only

Supplementary Table 1. Characteristics of study participants, free living Japanese volunteers, from September, 2013 to September 2015.

Locations (Prefectures)		Men		Women	
		N	Mean age	N	Mean age
Nationwide		51335	51.4	13201	51.0
Northern Japan	Hokkaido	1471	50.6	430	50.4
	Aomori	311	48.2	74	50.8
	Iwate	392	51.9	61	53.3
	Miyagi	846	50.3	266	46.4
	Akita	246	50.7	43	54.5
	Yamagata	286	50.2	56	54.8
	Fukushima	566	52.2	182	56.8
Eastern Japan	Ibaraki	1161	50.9	277	50.2
	Tochigi	753	49.5	156	49.7
	Gunma	613	50.9	143	50.9
	Saitama	3422	51.3	852	49.9
	Chiba	3189	51.2	817	50.7
	Tokyo	9673	50.7	2526	50.2

For peer review only

2

	Kanagawa	6275	51.5	1430	51.0
Central Japan	Niigata	599	50.5	128	50.3
	Toyama	302	51.2	72	50.2
	Ishikawa	351	55.6	70	49.9
	Fukui	179	51.5	44	51.8
	Yamanashi	243	53.0	50	56.1
	Nagano	701	51.9	131	51.7
	Gifu	591	51.8	139	51.9
	Shizuoka	1177	51.3	274	51.5
	Aichi	2866	51.5	705	51.2
	Mie	577	52.0	160	49.1
Western Japan	Shiga	561	51.4	118	50.1
	Kyoto	1274	51.1	346	51.2
	Osaka	3194	51.8	944	50.8
	Hyogo	2066	51.9	581	51.3
	Nara	448	53.4	115	53.6
	Wakayama	217	52.1	68	54.1
	Tottori	138	51.6	39	49.6

For peer review only

Shimane	155	51.8	47	51.2
Okayama	576	51.7	162	52.4
Hiroshima	993	52.4	210	51.2
Yamaguchi	357	52.8	110	51.0
Tokushima	187	50.4	62	48.5
Kagawa	312	50.9	87	51.5
Ehime	367	50.1	111	50.2
Kouchi	182	54.5	60	54.5
Fukuoka	1479	51.7	418	50.9
Saga	175	52.4	44	46.5
Nagasaki	309	51.2	87	52.7
Kumamoto	397	50.5	111	49.2
Oita	260	52.6	68	51.1
Miyazaki	204	51.6	60	47.1
Kagoshima	331	49.8	100	49.7
Okinawa	363	47.0	167	45.7

For peer review only

4

Supplementary Table 2. Monthly means (and standard errors) of home morning blood pressure from September 2013 to September 2015.

		Men					Women					Temp
		N	Systolic BP [mmHg]		Diastolic BP [mmHg]		N	Systolic BP [mmHg]		Diastolic BP [mmHg]		[C]
			Mean	SE	Mean	SE		Mean	SE	Mean	SE	
2013	Sep	13184	130.1	0.12	82.9	0.09	2563	123.6	0.31	76.4	0.22	22.1
	Oct	13779	131.7	0.12	83.9	0.09	2681	126.2	0.31	78.2	0.22	17.7
	Nov	13878	134.2	0.12	85.3	0.09	2644	128.6	0.31	79.6	0.22	10.0
	Dec	13997	134.6	0.12	85.5	0.09	2677	129.0	0.30	79.8	0.21	5.1
2014	Jan	14578	134.4	0.11	85.2	0.09	2752	128.9	0.31	79.7	0.21	2.7

For peer review only

5

Feb	14702	134.1	0.11	85.0	0.09	2782	128.2	0.30	79.3	0.21	3.1
Mar	15689	133.3	0.11	84.6	0.09	2954	127.4	0.28	78.7	0.20	7.0
Apr	16178	132.5	0.11	84.2	0.08	3071	126.6	0.28	78.2	0.20	11.4
May	16720	130.8	0.10	83.3	0.08	3202	124.8	0.27	77.2	0.19	17.2
Jun	16887	129.5	0.10	82.4	0.08	3224	123.3	0.26	76.0	0.19	21.2
Jul	17005	128.7	0.10	82.0	0.08	3238	122.3	0.26	75.4	0.20	24.4
Aug	17092	128.9	0.10	82.1	0.08	3261	122.4	0.26	75.5	0.20	25.3
Sep	17528	130.8	0.10	83.5	0.08	3423	124.4	0.26	77.0	0.19	20.7
Oct	17744	132.2	0.10	84.4	0.08	3439	126.5	0.26	78.4	0.19	16.2
Nov	17690	133.4	0.10	85.0	0.08	3378	127.9	0.27	79.3	0.19	11.2
Dec	17382	134.8	0.10	85.7	0.08	3295	129.4	0.28	80.0	0.19	4.4

For peer review only

2015	Jan	18133	134.2	0.10	85.3	0.08	3472	128.2	0.27	79.3	0.18	3.6
	Feb	18483	133.8	0.10	85.1	0.08	3537	127.9	0.26	79.3	0.18	3.5
	Mar	19266	133.1	0.10	84.8	0.08	3740	127.3	0.26	79.2	0.18	7.2
	Apr	20073	131.8	0.09	84.2	0.07	3901	125.9	0.25	78.4	0.17	12.7
	May	21140	130.4	0.09	83.4	0.07	4062	124.4	0.24	77.5	0.17	18.2
	Jun	21512	129.7	0.09	83.1	0.07	4211	123.5	0.23	77.1	0.17	20.3
	Jul	21532	128.6	0.09	82.5	0.07	4112	122.1	0.23	76.2	0.18	24.3
	Aug	21388	129.2	0.09	82.8	0.07	4164	122.8	0.24	76.6	0.17	25.1
	Sep	21408	130.3	0.09	83.7	0.07	4211	124.1	0.24	77.7	0.17	20.9

BP: blood pressure, C: degree Celsius, SE: Standard error, Temp: temperature, Jan: January, Feb: February, Mar: March, Apr: April, Jun: June, Jul: July, Aug: August, Sep: September, Oct: October, Nov: November, Dec: December

For peer review only

Supplementary Table 3. Monthly means (and standard errors) of home evening blood pressure from September 2013 to September 2015.

		Men					Women					Temp
		N	Systolic BP [mmHg]		Diastolic BP [mmHg]		N	Systolic BP [mmHg]		Diastolic BP [mmHg]		[C]
			Mean	SE	Mean	SE		Mean	SE	Mean	SE	
2013	Sep	10993	125.1	0.14	77.2	0.11	2210	120.2	0.34	72.6	0.24	22.8
	Oct	11588	126.4	0.13	78.2	0.11	2340	122.6	0.34	74.5	0.24	18.3
	Nov	11713	128.6	0.14	79.6	0.11	2362	124.7	0.33	76.0	0.24	11.1
	Dec	11872	128.8	0.14	79.6	0.11	2411	125.0	0.33	75.9	0.23	6.3
2014	Jan	12518	129.1	0.13	79.8	0.10	2519	125.2	0.32	76.0	0.23	4.1

For peer review only

For peer review only

Feb	12500	128.5	0.13	79.3	0.10	2481	124.5	0.32	75.6	0.23	4.6
Mar	13513	128.1	0.12	79.1	0.10	2689	124.3	0.31	75.3	0.22	8.4
Apr	13553	127.0	0.12	78.5	0.10	2660	123.1	0.31	74.5	0.22	12.8
May	13993	125.2	0.12	77.3	0.10	2778	121.4	0.30	73.2	0.22	17.7
Jun	14008	124.1	0.12	76.5	0.10	2695	120.2	0.30	72.1	0.21	21.5
Jul	13855	123.6	0.12	76.1	0.10	2710	119.3	0.30	71.7	0.22	24.9
Aug	13953	124.5	0.12	76.7	0.10	2730	120.1	0.30	72.1	0.22	25.4
Sep	14328	125.3	0.12	77.5	0.10	2837	121.0	0.30	73.2	0.22	21.6
Oct	14614	126.7	0.12	78.5	0.09	2902	123.4	0.30	75.0	0.21	17.2
Nov	14592	128.0	0.12	79.4	0.10	2816	125.0	0.32	76.0	0.22	12.4
Dec	14392	128.9	0.12	79.7	0.10	2761	125.8	0.32	76.4	0.23	5.2

2015	Jan	15193	129.0	0.12	79.7	0.09	3020	125.1	0.30	75.9	0.21	4.5
	Feb	15374	128.7	0.12	79.7	0.09	3001	124.9	0.30	76.0	0.21	4.8
	Mar	15975	128.0	0.11	79.3	0.09	3183	123.9	0.29	75.6	0.21	8.6
	Apr	16447	126.7	0.11	78.5	0.09	3258	123.3	0.29	75.2	0.20	13.4
	May	17167	125.1	0.11	77.5	0.09	3394	120.7	0.27	73.5	0.19	18.7
	Jun	17282	124.7	0.11	77.4	0.09	3452	120.4	0.27	73.4	0.20	20.5
	Jul	17143	124.1	0.11	76.9	0.09	3306	119.4	0.27	72.8	0.21	24.6
	Aug	16891	124.9	0.11	77.5	0.09	3317	120.4	0.27	73.3	0.20	25.4
	Sep	16927	125.4	0.11	78.1	0.09	3378	121.3	0.27	74.1	0.20	21.3

BP: blood pressure, C: degree Celsius, SE: Standard error, Temp: temperature, Jan: January, Feb: February, Mar: March, Apr: April, Jun: June, Jul: July, Aug: August, Sep: September, Oct: October, Nov: November, Dec: December

For peer review only

Supplementary Table 4. Monthly means (and standard errors) of home morning blood pressure by age groups (ages less than 60 years) from September 2013 to September 2015.

		Men					Women					Temp
		N	Systolic BP [mmHg]		Diastolic BP [mmHg]		N	Systolic BP [mmHg]		Diastolic BP [mmHg]		[C]
			Mean	SE	Mean	SE		Mean	SE	Mean	SE	
2013	Sep	9546	130.2	0.14	84.7	0.11	1706	122.1	0.39	77.5	0.29	22.1
	Oct	9972	131.7	0.14	85.7	0.11	1782	124.7	0.39	79.4	0.28	17.7
	Nov	10003	134.0	0.14	87.1	0.11	1748	126.5	0.38	80.6	0.28	10.0
	Dec	10054	134.6	0.14	87.3	0.11	1736	127.0	0.38	81.0	0.28	5.1
2014	Jan	10461	134.3	0.14	87.0	0.10	1808	126.8	0.37	80.9	0.28	2.7

For peer review only

Feb	10570	134.1	0.14	86.8	0.10	1812	126.0	0.37	80.4	0.28	3.1
Mar	11211	133.4	0.13	86.5	0.10	1915	125.2	0.35	79.7	0.26	7.0
Apr	11556	132.7	0.13	86.1	0.10	1971	124.2	0.34	79.3	0.26	11.3
May	11925	131.0	0.12	85.2	0.10	2079	122.6	0.33	78.2	0.25	17.2
Jun	11961	129.8	0.13	84.5	0.10	2111	121.6	0.33	77.3	0.25	21.2
Jul	12008	129.2	0.12	84.1	0.10	2119	120.9	0.34	76.7	0.26	24.4
Aug	11991	129.5	0.12	84.3	0.10	2129	121.0	0.34	76.9	0.26	25.3
Sep	12338	131.1	0.13	85.6	0.10	2254	122.6	0.33	78.2	0.25	20.7
Oct	12464	132.4	0.12	86.4	0.09	2245	124.8	0.33	79.7	0.25	16.2
Nov	12421	133.5	0.12	87.0	0.09	2174	126.0	0.34	80.6	0.26	11.2
Dec	12112	135.0	0.13	87.8	0.10	2097	127.4	0.35	81.3	0.26	4.4

For peer review only

2015	Jan	12672	134.3	0.12	87.2	0.09	2239	126.1	0.34	80.4	0.24	3.6
	Feb	12922	133.9	0.12	87.0	0.09	2309	125.7	0.33	80.2	0.24	3.5
	Mar	13438	133.3	0.12	86.8	0.09	2451	125.4	0.32	80.3	0.23	7.2
	Apr	14019	132.0	0.11	86.1	0.09	2568	124.0	0.32	79.5	0.23	12.7
	May	14879	130.6	0.11	85.3	0.08	2712	122.8	0.30	78.8	0.22	18.2
	Jun	15186	130.0	0.11	85.0	0.08	2826	122.0	0.29	78.5	0.22	20.3
	Jul	15141	129.2	0.11	84.5	0.08	2785	121.1	0.30	77.8	0.23	24.3
	Aug	14955	129.7	0.11	84.9	0.08	2816	121.4	0.30	77.9	0.22	25.1
	Sep	14986	130.6	0.11	85.8	0.08	2844	122.3	0.30	78.9	0.21	20.9

BP: blood pressure, C: degree Celsius, SE: Standard error, Temp: temperature, Jan: January, Feb: February, Mar: March, Apr: April, Jun: June, Jul: July, Aug: August, Sep: September, Oct: October, Nov: November, Dec: December

For peer review only

Supplementary Table 5. Monthly means (and standard errors) of home morning blood pressure by age groups (ages 60 years or older) from September 2013 to September 2015.

		Men					Women					Temp
		N	Systolic BP [mmHg]		Diastolic BP [mmHg]		N	Systolic BP [mmHg]		Diastolic BP [mmHg]		[C]
			Mean	SE	Mean	SE		Mean	SE	Mean	SE	
2013	Sep	3638	129.7	0.22	78.0	0.15	857	126.7	0.49	74.2	0.31	22.1
	Oct	3807	131.9	0.22	79.3	0.15	899	129.3	0.48	75.9	0.31	17.7
	Nov	3875	134.6	0.22	80.7	0.15	896	132.6	0.50	77.6	0.32	10.0
	Dec	3943	134.6	0.22	80.8	0.15	941	132.5	0.47	77.6	0.30	5.0
2014	Jan	4117	134.7	0.21	80.8	0.15	944	133.0	0.50	77.5	0.31	2.8

For peer review only

Feb	4132	134.1	0.21	80.4	0.15	970	132.3	0.49	77.4	0.31	3.1
Mar	4478	133.1	0.20	80.0	0.14	1039	131.4	0.43	77.0	0.29	7.1
Apr	4622	132.1	0.19	79.5	0.14	1100	130.8	0.45	76.5	0.28	11.4
May	4795	130.2	0.18	78.5	0.13	1123	129.0	0.43	75.2	0.27	17.1
Jun	4926	128.5	0.18	77.5	0.13	1113	126.7	0.41	73.7	0.26	21.2
Jul	4997	127.4	0.18	76.8	0.13	1119	125.1	0.40	72.9	0.27	24.5
Aug	5101	127.6	0.18	77.0	0.13	1132	125.0	0.41	72.9	0.27	25.3
Sep	5190	129.9	0.18	78.5	0.13	1169	127.9	0.43	74.6	0.27	20.7
Oct	5280	131.9	0.18	79.7	0.13	1194	129.8	0.42	76.0	0.26	16.3
Nov	5269	133.0	0.18	80.3	0.13	1204	131.2	0.42	77.0	0.27	11.2
Dec	5270	134.6	0.18	81.0	0.13	1198	132.9	0.44	77.8	0.27	4.3

For peer review only

2015	Jan	5461	134.1	0.18	80.8	0.12	1233	132.2	0.42	77.3	0.26	3.5
	Feb	5561	133.6	0.17	80.7	0.12	1228	131.9	0.43	77.4	0.27	3.5
	Mar	5828	132.7	0.17	80.2	0.12	1289	131.0	0.41	77.1	0.26	7.2
	Apr	6054	131.4	0.16	79.5	0.12	1333	129.4	0.39	76.2	0.25	12.7
	May	6261	130.0	0.16	78.8	0.12	1350	127.5	0.37	74.9	0.24	18.1
	Jun	6326	129.0	0.16	78.4	0.11	1385	126.5	0.36	74.3	0.24	20.3
	Jul	6391	127.3	0.15	77.5	0.11	1327	124.3	0.37	73.0	0.25	24.3
	Aug	6433	128.0	0.15	77.8	0.11	1348	125.8	0.38	73.8	0.25	25.0
	Sep	6422	129.5	0.15	79.0	0.11	1367	127.9	0.38	75.3	0.25	20.9

BP: blood pressure, C: degree Celsius, SE: Standard error, Temp: temperature, Jan: January, Feb: February, Mar: March, Apr: April, Jun: June, Jul: July, Aug: August, Sep: September, Oct: October, Nov: November, Dec: December

For peer review only

STROBE Statement—checklist of items that should be included in reports of observational studies

	Item No	Recommendation	Page/Line Number
**Title and abstract**	1	(*a*) Indicate the study's design with a commonly used term in the title or the abstract	Page 3 / lines 9-18
		(*b*) Provide in the abstract an informative and balanced summary of what was done and what was found	Page 3 / line 2 – Page 4 / line 12
**Introduction**			
Background/rationale	2	Explain the scientific background and rationale for the investigation being reported	Page 6 / line 2 – Page7 / line 2
Objectives	3	State specific objectives, including any prespecified hypotheses	Page 7 / lines 3 –8
**Methods**			
Study design	4	Present key elements of study design early in the paper	Page 7 / line 9 – Page 10 / line 4
Setting	5	Describe the setting, locations, and relevant dates, including periods of recruitment, exposure, follow-up, and data collection	Page 7 / line 9 – Page 9 / line 13
Participants	6	(*a*) *Cohort study*—Give the eligibility criteria, and the sources and methods of selection of participants. Describe methods of follow-up<br>*Case-control study*—Give the eligibility criteria, and the sources and methods of case ascertainment and control selection. Give the rationale for the choice of cases and controls<br>*Cross-sectional study*—Give the eligibility criteria, and the sources and methods of selection of participants	Page 9 / line 12 – Page 10 / line 1
		(*b*) *Cohort study*—For matched studies, give matching criteria and number of exposed and unexposed<br>*Case-control study*—For matched studies, give matching criteria and the number of controls per case	NA
Variables	7	Clearly define all outcomes, exposures, predictors, potential confounders, and effect modifiers. Give diagnostic criteria, if applicable	Page 10 / lines 2 – 14
Data sources/ measurement	8*	For each variable of interest, give sources of data and details of methods of assessment (measurement). Describe comparability of assessment methods if there is more than one group	Page 7 / line 9 – Page 10 / line 7
Bias	9	Describe any efforts to address potential sources of bias	Page 8 / lines 9 – 13, Page 10 / line 15 – Page 11 / line 1
Study size	10	Explain how the study size was arrived at	Page 9 / line 12 – Page 10 / line 1
Quantitative variables	11	Explain how quantitative variables were handled in the analyses. If applicable, describe which groupings were chosen and why	NA
Statistical methods	12	(*a*) Describe all statistical methods, including those used to control for confounding	Page 10 / lines 8 – 14
		(*b*) Describe any methods used to examine subgroups and interactions	Page 10 / lines 8 – 10
		(*c*) Explain how missing data were addressed	Page 9 / line 12 – Page 10 / line 1
		(*d*) *Cohort study*—If applicable, explain how loss to follow-up was addressed<br>*Case-control study*—If applicable, explain how matching of cases and controls was addressed<br>*Cross-sectional study*—If applicable, describe analytical methods taking account of sampling strategy	Page 9 / line 12 – Page 10 / line 14

For peer review only

		(*e*) Describe any sensitivity analyses	Page 10 / lines 8-10
**Results**			
Participants	13*	(a) Report numbers of individuals at each stage of study—eg numbers potentially eligible, examined for eligibility, confirmed eligible, included in the study, completing follow-up, and analysed	Page 11 / lines 4 – 6
		(b) Give reasons for non-participation at each stage	NA
		(c) Consider use of a flow diagram	NA
Descriptive data	14*	(a) Give characteristics of study participants (eg demographic, clinical, social) and information on exposures and potential confounders	Page 11 / lines 4-6, Supplemental Table 1
		(b) Indicate number of participants with missing data for each variable of interest	NA
		(c) *Cohort study*—Summarise follow-up time (eg, average and total amount)	NA
Outcome data	15*	*Cohort study*—Report numbers of outcome events or summary measures over time	NA
		Case-control study—Report numbers in each exposure category, or summary measures of exposure	NA
		Cross-sectional study—Report numbers of outcome events or summary measures	Supplemental Table 2-5
Main results	16	(*a*) Give unadjusted estimates and, if applicable, confounder-adjusted estimates and their precision (eg, 95% confidence interval). Make clear which confounders were adjusted for and why they were included	Page 11 / lines 8-16, Supplemental Table 1-5
		(*b*) Report category boundaries when continuous variables were categorized	NA
		(*c*) If relevant, consider translating estimates of relative risk into absolute risk for a meaningful time period	NA
Other analyses	17	Report other analyses done—eg analyses of subgroups and interactions, and sensitivity analyses	NA
**Discussion**			
Key results	18	Summarise key results with reference to study objectives	Page 14 / lines 12-18
Limitations	19	Discuss limitations of the study, taking into account sources of potential bias or imprecision. Discuss both direction and magnitude of any potential bias	Page 15 / line 18– Page 16 / line 6
Interpretation	20	Give a cautious overall interpretation of results considering objectives, limitations, multiplicity of analyses, results from similar studies, and other relevant evidence	Page 14 / line 19 – Page 16 / line 14
Generalisability	21	Discuss the generalisability (external validity) of the study results	Page 16 / lines 7–14
**Other information**			
Funding	22	Give the source of funding and the role of the funders for the present study and, if applicable, for the original study on which the present article is based	Page 21 / lines 5-16

*Give information separately for cases and controls in case-control studies and, if applicable, for exposed and unexposed groups in cohort and cross-sectional studies.

Note: An Explanation and Elaboration article discusses each checklist item and gives methodological background and published examples of transparent reporting. The STROBE checklist is best used in conjunction with this article (freely available on the Web sites of PLoS Medicine at http://www.plosmedicine.org/, Annals of Internal Medicine at http://www.annals.org/, and Epidemiology at http://www.epidem.com/). Information on the STROBE Initiative is available at www.strobe-statement.org.

For peer review only

Seasonal variation in home blood pressure: findings from nationwide web-based monitoring in Japan

Journal:	*BMJ Open*
Manuscript ID	bmjopen-2017-017351.R1
Article Type:	Research
Date Submitted by the Author:	03-Aug-2017
Complete List of Authors:	Iwahori, Toshiyuki; OMRON HEALTHCARE Co., Ltd, ; SHIGA UNIVERSITY of MEDICAL SCIENCE, HEALTH SCIENCE<br>Miura, katsuyuki; Shiga University of Medical Science, Health Science<br>Obayashi, Keiichi; Omron Kabushiki Kaisha<br>Ohkubo, Takayoshi<br>Nakajima, Hiroshi; Omron Kabushiki Kaisha<br>Shiga, Toshikazu; OMRON HEALTHCARE Co., Ltd.,<br>Ueshima, Hirotsugu; Shiga University of Medical Science, Health Science
**Primary Subject Heading**:	Public health
Secondary Subject Heading:	Public health, Cardiovascular medicine
Keywords:	PUBLIC HEALTH, Hypertension < CARDIOLOGY, EPIDEMIOLOGY

SCHOLARONE™ Manuscripts

For peer review only

Title:

Seasonal variation in home blood pressure: findings from nationwide web-based monitoring in Japan

Authors:

Toshiyuki Iwahori, PhD [1,2*], Katsuyuki Miura, MD [1,3], Keiichi Obayashi, MSE [4], Takayoshi Ohkubo, MD [5], Hiroshi Nakajima, PhD [4], Toshikazu Shiga, PhD [2], Hirotsugu Ueshima, MD [1,3]

Affiliations:

[1] Department of Public Health, Shiga University of Medical Science, Otsu, Japan,

[2] OMRON Healthcare Co., Ltd., Muko, Japan,

[3] Center for Epidemiologic Research in Asia, Shiga University of Medical Science, Otsu, Japan,

[4] OMRON Corporation, Kizugawa, Japan,

[5] Department of Hygiene and Public Health, Teikyo University School of Medicine, Tokyo, Japan

For peer review only

Full address:

[1] Seta Tsukinowa-cho, Otsu, Shiga, 520-2192, Japan,

[2] 53, Kunotsubo, Terado-cho, Muko, Kyoto, 617-0002, Japan,

[3] Seta Tsukinowa-cho, Otsu, Shiga, 520-2192, Japan,

[4] 9-1 Kizugawadai, Kizugawa, Kyoto, 619-0283, Japan,

[5] 2-11-1 Kaga, Itabashi-ku, Tokyo, 173-8605, Japan

Corresponding author: Toshiyuki Iwahori, PhD

Department of Public Health

Shiga University of Medical Science

Seta Tsukinowa-cho, Otsu, Shiga 520 2192, Japan

Tel.: +81 77 548 2191

Fax: +81 77 543 9732

E-mail address: iwahori@belle.shiga-med.ac.jp

For peer review only

Abstract

Objectives: Our aim was to assess seasonal variation in home blood pressure (BP) among free-living nationwide users using home BP values accumulated from web-based health care platform established in Japan.

Settings: An observational study. OMRON Healthcare has been developing web-based personal health care record systems in Japan since November 2010; over two million voluntary participants had joined this platform in September 2015. Nationwide home BP measured by oscillometric-type electronic sphygmomanometers from over 110 thousand voluntary participants have been collected to the system from devices.

Participants: Seasonal variation in home BP was evaluated among 64,536 (51,335 men, 13,201 women; mean age, 52.9 years) free-living nationwide users whom automatically and simultaneously transmitted data to the system from devices.

Primary outcome measures: Mean monthly and weekly home BP

Results: Changes in BP were associated with seasonal changes in temperature. Highest and lowest BP was observed in December and July, respectively. Substantial seasonal differences in the mean values of morning and evening home systolic BP between summer and winter were 6.2 mmHg and 5.5 mmHg in men, and 7.3 mmHg and 6.5 mmHg in women. Seasonal variation was a little larger in older (7.3 mmHg in men, 8.7 mmHg in women) than in younger individuals (5.8 mmHg in men, 6.5 mmHg in

For peer review only

women). BP from February to July showed approximately 1.5 mmHg lower than the value from August to December.

Conclusions: Web-based healthcare platform has enabled easier monitoring of population-wide BP. Tighter BP control is necessary in winter than in summer, and especially in colder climate toward winter than toward summer. New technologies using web-based self-monitoring systems for health-related indexes are expected to initiate a new phase of cardiovascular disease prevention, and public health promotion.

(281/300 words)

For peer review only

Strengths and limitations of this study

- Frequent nationwide home BP monitoring by the web-based platform enabled to see seasonal variation of home BP in monthly and weekly changes in large sample (>60,000) from non-medical settings.
- Nationwide home BP data automatically collected and electronically transmitted simultaneously to the web-based home BP monitoring platform minimized the selection bias and reflected the casual BP values in the real world.

- The majority of the participants might have been health conscious individuals.
- Timing and frequency of the measurement, and the room temperature during home BP measurements remain unknown.

For peer review only

Introduction

The management of blood pressure (BP) is one of the most important strategies for the prevention, and management of cardiovascular diseases (CVD) around the world.[1–6] Although BP management has been generally based on BP values measured in the clinical setting by medical professionals, home BP monitoring by individuals is becoming increasingly important in diagnosis, and control of arterial hypertension.[4–9]

Electronic devices for home BP measurement were developed in the 1990s. Companies that manufacture devices for home BP measurement tend to concentrate in the East Asian region (Japan, South Korea, and Taiwan). Japan is unique for its high coverage of home BP measurement; nearly 20 million devices were estimated to exist out of approximately 50 million households by the sales record of BP monitors sold by OMRON Healthcare (Kyoto, Japan) in 2012.

Under these conditions, OMRON Healthcare, a health care device manufacturer, developed a web-based health care platform in 2010 that consumers can join voluntarily after purchasing one of its products. OMRON Healthcare's recently developed devices for home BP measurement send all measured values to a web-based database electronically via personal computers, or mobile phones. From 2010 to 2015, more than two million people in Japan have voluntarily participated in this web-based health care platform. Nowadays, these newly established technologies and platforms enable to

For peer review only

collect real world data simultaneously and show the possibilities of lowering the hurdles to detect useful population-wide information and provide new approach for public health authorities.

Multiple observational studies have examined the seasonal variation of BP in clinical settings;[10] however, there is no report on seasonal variation using nationwide large sample of home BP monitoring data from non-medical and uncontrolled settings. Thus, our aims were to assess seasonal variation in home blood pressure among free-living nationwide users by real world data collection by this web-based health care platform, and to see the applications for public health policy making.

Materials and Methods

Web-based personal healthcare record systems

OMRON Healthcare has been developing web-based personal health care record systems. One of its application services is named as WellnessLINK, which was deployed on November 1st, 2010. The participants can register and leave the web based healthcare platform by their freewill in anytime. WellnessLINK utilizes many kinds of health care devices connected to the internet via personal computers, and mobile phones to collect various types of healthcare data into a database.

For peer review only

The WellnessLINK system records BP values measured at home using oscillometric-type electronic sphygmomanometers, including an upper arm BP monitor (HEM-7510C, HEM-7500F, HEM-7324C, HEM-7320F, HEM-7280C, HEM-7270C, HEM-7250-IT; OMRON Healthcare), an upper arm BP monitor with an automatic cuff wrapping system (HEM-1025; OMRON Healthcare), and a wrist BP monitor (HEM-6321T, HEM-6320T, HEM-6310F, HEM-6300F; OMRON Healthcare). These devices can be share used with family members by differentiating the user identification numbers set at each device among family members. All devices have been validated and approved by the Ministry of Health, Labour and Welfare, Japan [11], and certified as having been adjusted to the ISO/IEC 81060-2 standards [12]. The users receive an instruction manual for BP measurement based on the Japanese Society of Hypertension Guidelines for Self-Monitoring of Blood Pressure at Home.[7] Users then access the website via their personal computer, or mobile phone, and register after approving a privacy policy agreement. For personal computer users, BP data are transmitted to the system via internet by connecting a sphygmomanometer using either by Universal Serial Bus (USB), Near Field Communication (NFC), Bluetooth, or WiFi. For mobile phone users, BP data are transmitted to the system via the internet through either NFC, or Bluetooth. In most of the devices, an active data update is required to transmit the data automatically from the devices to the system by the individuals. However, some

For peer review only

recent devices with WiFi connections transfer data both simultaneously, and automatically to the system. Self-reported data, manually inputted by a user, is also accepted. All BP data from users who voluntarily register with this service are sent to the computer server at OMRON Healthcare.

The WellnessLINK system provides various kinds of health support programs, including antihypertensive health care, metabolic syndrome prevention, dietary improvement, and physical activity. In the antihypertensive health care program, WellnessLINK provides personalized daily, weekly, monthly, and yearly graphs of BP, which are useful for individual BP management. In addition, feedback is provided to the users after accumulated data is analyzed.

Blood pressure data analysis

A total of 2,164,321 individuals in Japan registered in the WellnessLINK system by the end of September 2015. Among these users, 110,454 sent BP data to the system automatically from BP measurement devices, or manually. Self-reported data that were manually inputted by users were excluded from the data analysis in this study. In this report, we analyzed 41.7 million BP measurement data from 64,536 registered users (51,335 men, 13,201 women; mean age, 52.9 years) who sent their all measured BP data

For peer review only

automatically. Among these users, 24% were treated with anti-hypertensive medication.

BP values measured at home between 4:00 AM, and 9:59 AM were defined as "morning BP," while those measured at home between 7:00 PM, and 1:59 AM were defined as "evening BP." Individual mean morning BP values were calculated using the first measured BP value in the morning, and measured within ten minutes later. Individuals mean evening BP values were calculated using the last measured BP value in the evening, and values measured within ten minutes previously. The monthly means of home BP was calculated by averaging individuals' home BP of overall participants.

Descriptive statistics values such as mean monthly systolic and diastolic BPs (and standard errors) were calculated by sex, and age groups for two years, from September 2013 to September 2015. Monthly mean BP changes for two years were shown in figures along with mean outdoor temperatures. Intermonth differences in BP were assessed analysis of variance. The t test for unpaired observations was used to determine the significance of the difference, after Bonferroni correction for multiple comparisons. All P values were two sided, and P values < 0.05 was taken as the level of statistical significance. Mean outdoor temperatures were calculated using outdoor temperatures at the time of each measurement in the capital city of the prefecture in which each participant lived, referenced from the database of the Japan Meteorological Agency.

The participants of this study had voluntarily filled out the online consent form,

For peer review only

agreed to send their data to the data server managed by OMRON Healthcare to use the WellnessLINK system service, and agreed with the condition that these data obtained by the WellnessLINK system would be analyzed for medical research use after data anonymization. The ethics committee of the Omron Healthcare Company approved the study protocol.

For peer review only

Results

Mean monthly morning and evening home systolic blood pressure

A total of 64,536 Japanese registered users sent their all measured BP data automatically from September 2013 to September 2015 from nationwide (Supplementary Table 1). Approximately 83% of the study participants were men, mean age was 54 years old, and the treatment rate among hypertensive people in the present study was approximately 39%; however these status remained relatively stable throughout the study period. Mean monthly morning and evening home systolic BP (SBP), as well as mean monthly temperatures for a two-year period, are shown in Figure 1. Seasonal variation was apparent in both morning, and evening home SBP in both sexes; values were higher in the winter, and lower in the summer (Figure 1, Supplemental Table 2-3, Supplemental Movie). The maximum winter-summer

difference was a little larger for morning SBP (6.2 mmHg in men, 7.3 mmHg in women) compared with evening SBP (5.5 mmHg in men, 6.5 mmHg in women). Similar seasonal variation was observed in morning SBP for younger, and older individuals (Figure 2, Supplemental Table 4-5), with the winter-summer difference being a little larger in older (7.3 mmHg in men, 8.7 mmHg in women) than in younger individuals (5.8 mmHg in men, 6.5 mmHg in women).

The amplitude of winter-summer difference was smaller in diastolic BP compared to SBP; however, similar trend of seasonal variation was observed in diastolic BP (Supplemental Table 2-5).

Monthly morning home systolic blood pressure and mean outdoor temperatures

The association between mean monthly morning home SBP and mean outdoor temperatures for a 1-year period in men was skewed and showed hysteresis loop characteristic (Figure 3 and Supplemental Table 2). Outside temperature was lowest in February (3.5°C) but similar from December (4.4°C) to February; however, morning home SBP lowered toward February (134.8 mmHg to 133.8 mmHg) from the highest recorded in December. Furthermore, outside temperature was highest in August (25.1°C) but similar from July (24.3°C) to August; however, morning home SBP was

For peer review only

higher in August (129.2 mmHg) than the lowest recorded in July (128.6 mmHg). Morning home SBP from February to July showed approximately 1.5mmHg lower than the value from August to December (e.g., morning home SBP was 1.5 mmHg higher in November [11.2°C] than in April [12.7°C]). There were statistical significances observed in BP between summer and winter months; however, there were a few intermonth combinations which did not reach statistical significance (March versus November, and May versus September). Similar findings were observed in evening home SBP and DBP, regardless of gender and age subgroups (Supplemental Table 2-5).

Discussion

Monitoring population-wide BP is important for public health, especially for CVD prevention.[1-6] Previous studies have shown approximately 2–10 mmHg, 4–8 mmHg, and 3 mmHg of seasonal variability in clinic BP, home BP, and 24-hour ambulatory BP (ABP), respectively.[13-22] Most clinics are temperature controlled; thus, likely do not represent daily climate conditions.[15,23] Seasonal variation in CVD incidence has been reported,[24–29] and seasonal variation in BP has been suggested to be associated with seasonal variation in the incidence of CVD.[23] Stamler et al. estimated the reduction in population BP for 2-5 mmHg deliver reduction in mortality of stroke,

mortality of coronary heart disease and total mortality for approximately 6-14%, 4-9% and 3-7%, respectively [30]. However, there was no system available for public health authorities to detect population-wide BP to plan out countermeasures, and announce alert for CVD prevention. Thus, acquiring nationwide "real world" home BP data is desirable.

The rapid diffusion of home BP monitoring has been aided by technological progress, the wider availability of measurement devices, increased awareness of the importance of regular BP monitoring, and recognition of the usefulness of home BP measurement by international hypertension management guidelines.[4–9] The National Health and Nutrition Survey of Japan showed that in 2010, 46% of adults, and 72% of hypertensive patients had measured their BP at home within the previous year.[31] Therefore, devices for home BP measurement have been spread nationwide, have been used more frequently in Japan than in any other country in the world. However, conventional data collection methods using self-recorded home BP may result in a bias, mainly underreported BP values.[7,32] The uniqueness of the system in the present study is that nationwide home BP data are automatically collected, and electronically transmitted simultaneously to the web-based home BP monitoring platform. This system enables the elimination of manual processes performed with substantial effort by clinical staff, and minimizes the possibility of a selection bias in the data collection. Thus, we

For peer review only

demonstrated the practical use of web-based healthcare platform which may reliably reflect the trend of population-wide BP as a “real world” data here in weekly and monthly analyses.

Findings from other previous studies suggest that outdoor temperature, and home central heating affect the seasonal variability of BP, especially during the winter.[16,22-23] In the present study, a parallel association was observed between seasonal variation in home BP (about 7 mmHg) and changes in outdoor temperature in this ”real world” data. This finding was consistent with previous report with smaller sample sized, lower frequent and manually collected data.[21] One interesting finding was that home BP tended higher for about 1.5 mmHg during autumn (summer to winter) than in spring (winter to summer). To the best of our knowledge, we are the first to report this hysteresis loop characteristic. Tighter BP control may be required during autumn than in spring.

Ideally, public health authorities would be able to notice population-wide changes in BP through monitoring population-wide “real world” home BP data by using this platform, and this would allow them to forecast CVD risk at appropriate times. Furthermore, public health authorities may also make announcements for clinicians, and practitioners to be cautious of over- and under-dose of anti-hypertensive medication by referencing the population-wide “real world” BP which was easily screened for

For peer review only

appropriate BP control. Such new approaches may initiate new phases for CVD prevention and public health promotion. In addition to devices for home BP measurement, adding varieties of lifestyle monitoring devices related to BP management (e.g., monitors for body weight, physical activity [33], sleep [34], body temperature, and urinary sodium-to-potassium ratio [35]) to this platform may support medical and public health experts to identify new realities, assess the concurrent population-wide CVD risk and plan out appropriate countermeasures. Therefore, by using this platform, much appropriate feedbacks from public health authorities could be expected to support individuals to take right action toward healthy lifestyle.

To the best of our knowledge, this is the first study to report seasonal variation from a large sample of "real world" home BP data from non-medical settings. Our findings demonstrated that the seasonal variation of "real world" home BP was independent of age. In this study, the participants were whom registered to use this web based healthcare platform; the participants might have higher socioeconomic status since they may have higher computer literacy and much health conscious people compared to others in general populations. Therefore, the seasonal variation of the "real world" home BP might have been underestimated due to the higher socioeconomic status of the study participants. Moreover, the participants in this web based healthcare platform can register and leave by their freewill in anytime, this may aggravates the

For peer review only

selection bias. However, the large sample size (>60,000), voluntary participation in the healthcare platform, measurement by a diverse range of individuals, various measurement times, including weekends, and non-clinical measurement settings are thought to minimize the possibility of selection bias in this study, and reflect casual BP values in the real world.

This study has some limitation. As the data obtained during the study was "real world" data, the room temperature during home BP measurements was unknown, some of the BP measurement devices might have been used by multiple users without differentiating the user identification numbers and the frequency and timing of BP measurements may have differed for each individual.

Based on the findings in this study, combining current public health policies with emerging technologies could be expected to help authorities monitor health-related indexes. To reduce CVD incidents, and for CVD prevention, we need to piece out the situation of individuals of entire population. Therefore, establishing web-based healthcare platform by public health authorities might be an alternative for obtaining eyes for active control of public health outcome. Initiation of a new phase for both CVD prevention and public health promotion may contribute to reduce nationwide medical expenditures.

For peer review only

(2525/3000words)

For peer review only

References

1. Lewington S, Clarke R, Qizilbash N, Peto R, Collins R; Prospective Studies Collaboration. Age-specific relevance of usual blood pressure to vascular mortality: a meta-analysis of individual data for one million adults in 61 prospective studies. *Lancet*. 2002 Dec 14;**360**(9349):1903-13.

2. Xie X, Atkins E, Lv J, et al. Effects of intensive blood pressure lowering on cardiovascular and renal outcomes: updated systematic review and meta-analysis. *Lancet*. 2016 Jan 30;**387**(10017):435-43.

3. Jaffe MG, Young JD. The Kaiser Permanente Northern California Story: Improving Hypertension Control From 44% to 90% in 13 Years (2000 to 2013). *J Clin Hypertens (Greenwich)*. 2016 Apr;18(4):260-1.

4. Mancia G, Fagard R, Narkiewicz K, et al. 2013 ESH/ESC guidelines for the management of arterial hypertension: the Task Force for the Management of Arterial Hypertension of the European Society of Hypertension (ESH) and of the European Society of Cardiology (ESC). *Blood Press* 2013; **22**: 193–278.

5. Armstrong C. JNC8 guidelines for the management of hypertension in adults. *Am Fam Physician*. 2014 Oct 1;**90**(7):503-4.

6. Shimamoto K, Ando K, Fujita T, et al. The Japanese Society of Hypertension Guidelines for the Management of Hypertension (JSH 2014). *Hypertens Res.* 2014 Apr;**37**(4):253-390. doi: 10.1038/hr.2014.20.

7. Imai Y, Kario K, Shimada K, et al. The Japanese Society of Hypertension Guidelines for Self-monitoring of Blood Pressure at Home (Second Edition). *Hypertens Res*. 2012 Aug;**35**(8):777-95. doi: 10.1038/hr.2012.56.

8. Parati G, Stergiou GS, Asmar R, et al. European Society of Hypertension guidelines for blood pressure monitoring at home: a summary report of the Second International Consensus Conference on Home Blood Pressure Monitoring. *J Hypertens.* 2008 Aug;**26**(8):1505-26.

9. Stergiou GS, Asayama K, Thijs L, et al. Prognosis of white-coat and masked

For peer review only

hypertension: International Database of HOme blood pressure in relation to Cardiovascular Outcome. *Hypertension*. 2014 Apr;63(4):675-82.

10. Shirasaki O, Terada H, Niwano K, Nakanishi T, Kanai M, Miyawaki Y, Souma T, Tanaka T, Kusunoki T. The Japan Home-health Apparatus Industrial Association: investigation of home-use electronic sphygmomanometers. *Blood Press Monit*. 2001 Dec;6(6):303-7.

11. INTERNATIONAL STANDARD ISO/IEC 81060-2: Non-invasive sphygmomanometers —Part 2: Clinical investigation of automated measurement type. ISO Copyright office, Geneva, Switzerland, 2013

12. Hanazawa T, Asayama K, Watabe D, Hosaka M, Satoh M, Yasui D, Obara T, Inoue R, Metoki H, Kikuya M, Imai Y, Ohkubo T. Seasonal variation in self-measured home blood pressure among patients on antihypertensive medications: HOMED-BP study. *Hypertens Res*. 2016 Sep 29. doi: 10.1038/hr.2016.133.

13. Rose G. Seasonal variation in blood pressure in man. *Nature.* 1961 Jan 21;**189**:235.

14. Brennan PJ, Greenberg G, Miall WE, Thompson SG. Seasonal variation in arterial blood pressure. *Br Med J (Clin Res Ed).* 1982 Oct 2;**285**(6346):919-23.

15. Madsen C, Nafstad P. Associations between environmental exposure and blood pressure among participants in the Oslo Health Study (HUBRO). *Eur J Epidemiol*. 2006;**21**(7):485-91.

16. Lewington S, Li L, Sherliker P, et al. Seasonal variation in blood pressure and its relationship with outdoor temperature in 10 diverse regions of China: the China Kadoorie Biobank. *J Hypertens.* 2012 Jul;**30**(7):1383-91.

17. Modesti PA, Morabito M, Bertolozzi I, et al. Weather-related changes in 24-hour blood pressure profile: effects of age and implications for hypertension management. *Hypertension*. 2006 Feb;**47**(2):155-61.

18. Barnett AG, Sans S, Salomaa V, Kuulasmaa K, Dobson AJ; WHO MONICA Project. The effect of temperature on systolic blood pressure. *Blood Press Monit.* 2007 Jun;**12**(3):195-203.

For peer review only

19. Sega R, Cesana G, Bombelli M, et al. Seasonal variations in home and ambulatory blood pressure in the PAMELA population. Pressione Arteriose Monitorate E Loro Associazioni. *J Hypertens.* 1998 Nov;**16**(11):1585-92.

20. Alpérovitch A, Lacombe JM, Hanon O, et al. Relationship between blood pressure and outdoor temperature in a large sample of elderly individuals: the Three-City study. *Arch Intern Med.* 2009 Jan 12;**169**(1):75-80.

21. Imai Y, Munakata M, Tsuji I, et al. Seasonal variation in blood pressure in normotensive women studied by home measurements. *Clin Sci (Lond).* 1996 Jan;**90**(1):55-60.

22. Murakami S, Otsuka K, Kono T, et al. Impact of outdoor temperature on prewaking morning surge and nocturnal decline in blood pressure in a Japanese population. *Hypertens Res.* 2011 Jan;**34**(1):70-3.

23. Cuspidi C, Ochoa JE, Parati G. Seasonal variations in blood pressure: a complex phenomenon. *J Hypertens.* 2012 Jul;**30**(7):1315-20.

24. Analitis A, Katsouyanni K, Biggeri A, et al. Effects of cold weather on mortality: results from 15 European cities within the PHEWE project. *Am J Epidemiol.* 2008 Dec 15;**168**(12):1397-408.

25. Danet S, Richard F, Montaye M, et al. Unhealthy effects of atmospheric temperature and pressure on the occurrence of myocardial infarction and coronary deaths. A 10-year survey: the Lille-World Health Organization MONICA project (Monitoring trends and determinants in cardiovascular disease). *Circulation.* 1999 Jul 6;**100**(1):E1-7.

26. van Rossum CT, Shipley MJ, Hemingway H, et al. Seasonal variation in cause-specific mortality: are there high-risk groups? 25-year follow-up of civil servants from the first Whitehall study. *Int J Epidemiol.* 2001 Oct;**30**(5):1109-16.

27. Shinkawa A, Ueda K, Hasuo Y, Kiyohara Y, Fujishima M. Seasonal variation in stroke incidence in Hisayama, Japan. *Stroke.* 1990 Sep;**21**(9):1262-7.

For peer review only

28. Sheth T, Nair C, Muller J, Yusuf S. Increased winter mortality from acute myocardial infarction and stroke: the effect of age. *J Am Coll Cardiol.* 1999 Jun;**33**(7):1916-9.

29. Turin TC, Kita Y, Rumana N, et al. Stroke case fatality shows seasonal variation regardless of risk factor status in a Japanese population: 15-year results from the Takashima Stroke Registry. *Neuroepidemiology*. 2009;**32**(1):53-60.

30. Stamler R. Implications of the INTERSALT study. *Hypertension*. 1991 Jan;17(1 Suppl):I16-20.

31. National Institute of Health and Nutrition. The National Health and Nutrition Survey Japan, 2010. Dai-ichi Shuppan Co. Ltd.: Tokyo, Japan, 2013 (in Japanese).

32. Mengden T, Hernandez Medina RM, Beltran B, et al. Reliability of reporting self-measured blood pressure values by hypertensive patients. *Am J Hypertens.* 1998 Dec;**11**(12):1413-7.

33. Tanaka C, Reilly JJ, Tanaka M, Tanaka S. Seasonal changes in objectively measured sedentary behavior and physical activity in Japanese primary school children. *BMC Public Health*. 2016 Sep 13;16:969.

34. Hashizaki M, Nakajima H, Kume K. Monitoring of Weekly Sleep Pattern Variations at Home with a Contactless Biomotion Sensor. *Sensors (Basel)*. 2015 Aug 3;15(8):18950-64.

35. Iwahori T, Ueshima H, Ohgami N, et al. Effectiveness of a self-monitoring device for urinary sodium/potassium ratio on dietary improvement in free-living adults: a randomized controlled trial. *J Epidemiol*. 2017 (in press).

For peer review only

Supplementary Information

Five supplementary tables and one supplementary movie. Supplementary movie shows the nationwide distribution of weekly mean home morning systolic blood pressure from September 2013 to September 2015.

Acknowledgements

This study was conducted by OMRON Healthcare Co., Ltd., and OMRON Corporation in cooperation with the Department of Public Health, Shiga University of Medical Science.

Funding

The running cost of this observational study was funded by OMRON Healthcare Co. Ltd.

Author contributions

KM, TO, HN, TS, HU contributed to the design of the study; KO, HN participated in data collection and data analysis; KM, KO, TI, TO and HU contributed to manuscript drafting; all authors participated in critical revisions of the manuscript; all authors approved the final version of the manuscript for submission.

For peer review only

Declaration of interests

TI and TS are employee of OMRON Healthcare Co., Ltd. KO and HN are employee of OMRON Corporation. Dr. Hirotsugu Ueshima served as a consultant for this project. Dr. Katsuyuki Miura received a research grant from OMRON Healthcare Co., Ltd.

For peer review only

Figure legends

Figure 1. Monthly means (and standard errors) of (A) home morning and (B) home evening systolic blood pressure (SBP) from September 2013 to September 2015. The maximum winter-summer difference was larger for morning SBP (6.2 mmHg in men, 7.3 mmHg in women) than for evening SBP (5.5 mmHg in men, 6.5 mmHg in women).

Figure 2. Monthly means (and standard errors) of home morning SBP by age groups from September 2013 to September 2015; (A) Participants aged less than 60 years; (B) participants aged 60 years or older. The winter-summer difference was larger in older participants (7.3 mmHg in men, 8.7 mmHg in women) than in younger participants (5.8 mmHg in men, 6.5 mmHg in women).

Figure 3. Association between monthly mean home morning SBP (and standard errors) and mean temperatures in each month from October 2014 to September 2015 in men. Home morning SBP was highest in winter and lowest in summer. Home morning SBP was higher in autumn than in spring, although outdoor temperatures were similar.

For peer review only

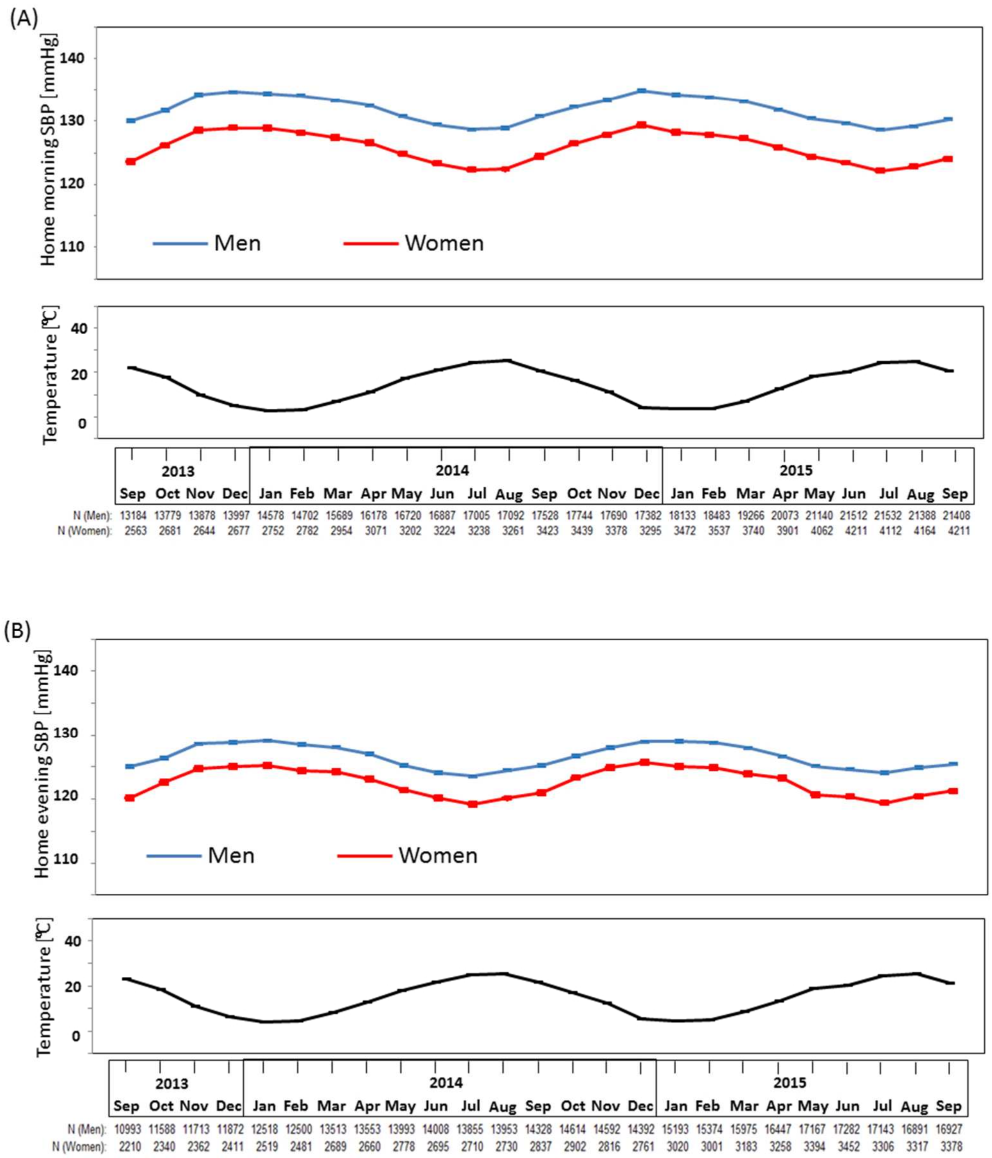

Figure 1

254x296mm (96 x 96 DPI)

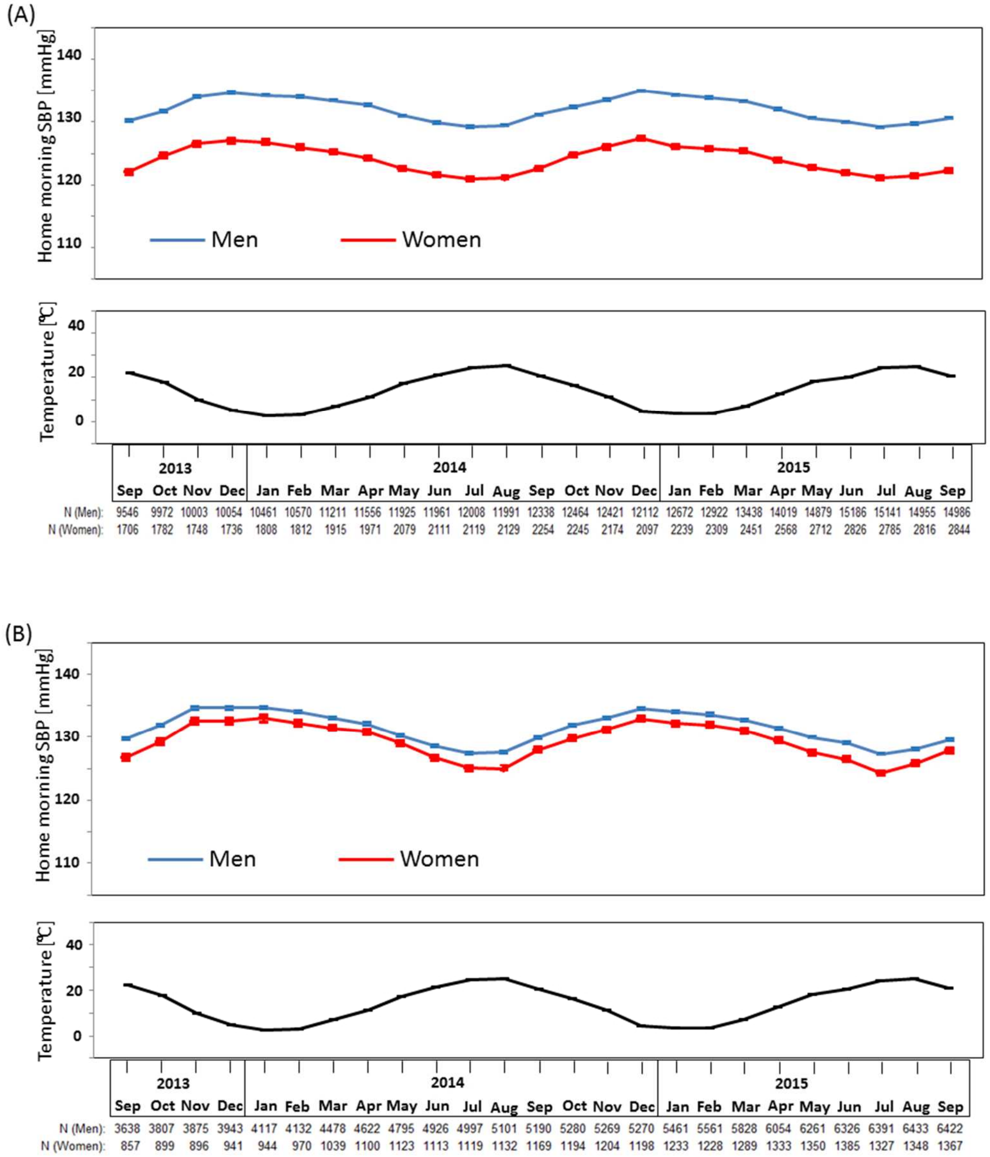

Figure 2

254x297mm (96 x 96 DPI)

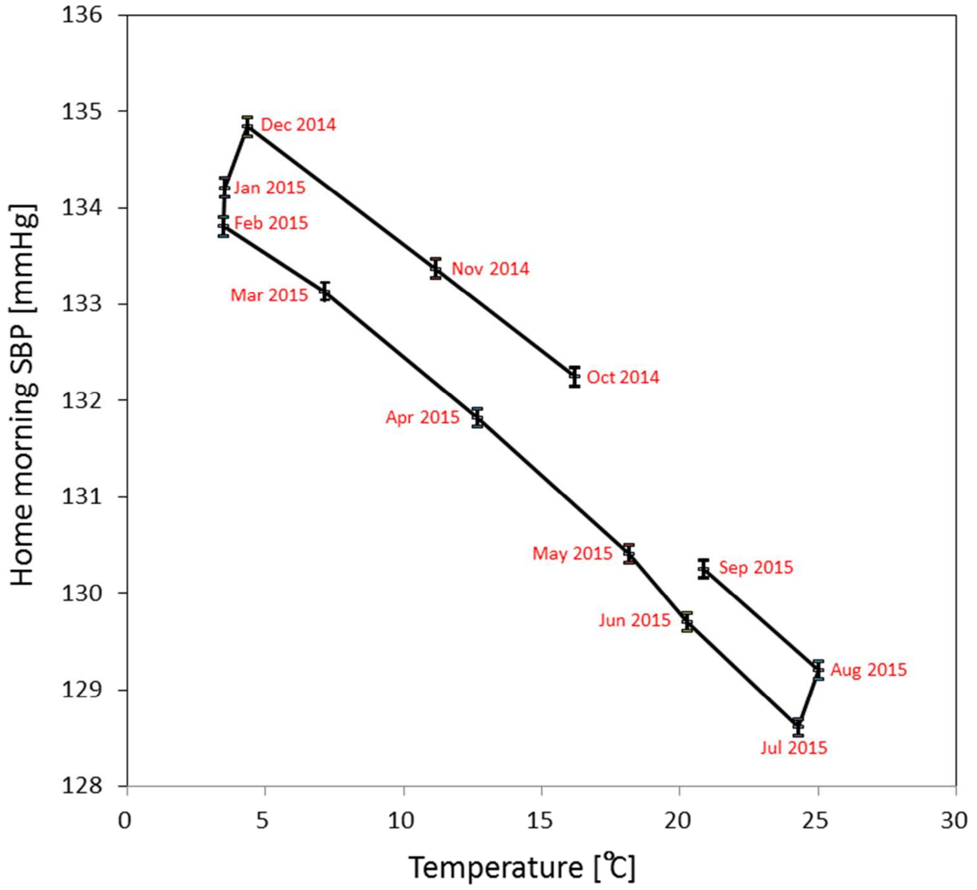

Figure 3

209x190mm (96 x 96 DPI)

only

Supplementary Table 1. Characteristics of study participants, free living Japanese volunteers, from September, 2013 to September 2015.

Locations (Prefectures)		Men N	Men Mean age	Women N	Women Mean age
Nationwide		51335	51.4	13201	51.0
Northern Japan	Hokkaido	1471	50.6	430	50.4
	Aomori	311	48.2	74	50.8
	Iwate	392	51.9	61	53.3
	Miyagi	846	50.3	266	46.4
	Akita	246	50.7	43	54.5
	Yamagata	286	50.2	56	54.8
	Fukushima	566	52.2	182	56.8
Eastern Japan	Ibaraki	1161	50.9	277	50.2
	Tochigi	753	49.5	156	49.7
	Gunma	613	50.9	143	50.9
	Saitama	3422	51.3	852	49.9
	Chiba	3189	51.2	817	50.7
	Tokyo	9673	50.7	2526	50.2

For peer review only

	Kanagawa	6275	51.5	1430	51.0
Central Japan	Niigata	599	50.5	128	50.3
	Toyama	302	51.2	72	50.2
	Ishikawa	351	55.6	70	49.9
	Fukui	179	51.5	44	51.8
	Yamanashi	243	53.0	50	56.1
	Nagano	701	51.9	131	51.7
	Gifu	591	51.8	139	51.9
	Shizuoka	1177	51.3	274	51.5
	Aichi	2866	51.5	705	51.2
	Mie	577	52.0	160	49.1
Western Japan	Shiga	561	51.4	118	50.1
	Kyoto	1274	51.1	346	51.2
	Osaka	3194	51.8	944	50.8
	Hyogo	2066	51.9	581	51.3
	Nara	448	53.4	115	53.6
	Wakayama	217	52.1	68	54.1
	Tottori	138	51.6	39	49.6

For peer review only

3

Shimane	155	51.8	47	51.2
Okayama	576	51.7	162	52.4
Hiroshima	993	52.4	210	51.2
Yamaguchi	357	52.8	110	51.0
Tokushima	187	50.4	62	48.5
Kagawa	312	50.9	87	51.5
Ehime	367	50.1	111	50.2
Kouchi	182	54.5	60	54.5
Fukuoka	1479	51.7	418	50.9
Saga	175	52.4	44	46.5
Nagasaki	309	51.2	87	52.7
Kumamoto	397	50.5	111	49.2
Oita	260	52.6	68	51.1
Miyazaki	204	51.6	60	47.1
Kagoshima	331	49.8	100	49.7
Okinawa	363	47.0	167	45.7

For peer review only

Supplementary Table 2. Monthly means (and standard errors) of home morning blood pressure from September 2013 to September 2015.

		Men					Women					Temp
		N	Systolic BP [mmHg]		Diastolic BP [mmHg]		N	Systolic BP [mmHg]		Diastolic BP [mmHg]		[C]
			Mean	SE	Mean	SE		Mean	SE	Mean	SE	
2013	Sep	13184	130.1	0.12	82.9	0.09	2563	123.6	0.31	76.4	0.22	22.1
	Oct	13779	131.7	0.12	83.9	0.09	2681	126.2	0.31	78.2	0.22	17.7
	Nov	13878	134.2	0.12	85.3	0.09	2644	128.6	0.31	79.6	0.22	10.0
	Dec	13997	134.6	0.12	85.5	0.09	2677	129.0	0.30	79.8	0.21	5.1
2014	Jan	14578	134.4	0.11	85.2	0.09	2752	128.9	0.31	79.7	0.21	2.7

For peer review only

Feb	14702	134.1	0.11	85.0	0.09	2782	128.2	0.30	79.3	0.21	3.1
Mar	15689	133.3	0.11	84.6	0.09	2954	127.4	0.28	78.7	0.20	7.0
Apr	16178	132.5	0.11	84.2	0.08	3071	126.6	0.28	78.2	0.20	11.4
May	16720	130.8	0.10	83.3	0.08	3202	124.8	0.27	77.2	0.19	17.2
Jun	16887	129.5	0.10	82.4	0.08	3224	123.3	0.26	76.0	0.19	21.2
Jul	17005	128.7	0.10	82.0	0.08	3238	122.3	0.26	75.4	0.20	24.4
Aug	17092	128.9	0.10	82.1	0.08	3261	122.4	0.26	75.5	0.20	25.3
Sep	17528	130.8	0.10	83.5	0.08	3423	124.4	0.26	77.0	0.19	20.7
Oct	17744	132.2	0.10	84.4	0.08	3439	126.5	0.26	78.4	0.19	16.2
Nov	17690	133.4	0.10	85.0	0.08	3378	127.9	0.27	79.3	0.19	11.2
Dec	17382	134.8	0.10	85.7	0.08	3295	129.4	0.28	80.0	0.19	4.4

For peer review only

2015	Jan	18133	134.2	0.10	85.3	0.08	3472	128.2	0.27	79.3	0.18	3.6
	Feb	18483	133.8	0.10	85.1	0.08	3537	127.9	0.26	79.3	0.18	3.5
	Mar	19266	133.1	0.10	84.8	0.08	3740	127.3	0.26	79.2	0.18	7.2
	Apr	20073	131.8	0.09	84.2	0.07	3901	125.9	0.25	78.4	0.17	12.7
	May	21140	130.4	0.09	83.4	0.07	4062	124.4	0.24	77.5	0.17	18.2
	Jun	21512	129.7	0.09	83.1	0.07	4211	123.5	0.23	77.1	0.17	20.3
	Jul	21532	128.6	0.09	82.5	0.07	4112	122.1	0.23	76.2	0.18	24.3
	Aug	21388	129.2	0.09	82.8	0.07	4164	122.8	0.24	76.6	0.17	25.1
	Sep	21408	130.3	0.09	83.7	0.07	4211	124.1	0.24	77.7	0.17	20.9

BP: blood pressure, C: degree Celsius, SE: Standard error, Temp: temperature, Jan: January, Feb: February, Mar: March, Apr: April, Jun: June, Jul: July, Aug: August, Sep: September, Oct: October, Nov: November, Dec: December

For peer review only

Supplementary Table 3. Monthly means (and standard errors) of home evening blood pressure from September 2013 to September 2015.

		Men					Women					Temp
		N	Systolic BP [mmHg]		Diastolic BP [mmHg]		N	Systolic BP [mmHg]		Diastolic BP [mmHg]		[C]
			Mean	SE	Mean	SE		Mean	SE	Mean	SE	
2013	Sep	10993	125.1	0.14	77.2	0.11	2210	120.2	0.34	72.6	0.24	22.8
	Oct	11588	126.4	0.13	78.2	0.11	2340	122.6	0.34	74.5	0.24	18.3
	Nov	11713	128.6	0.14	79.6	0.11	2362	124.7	0.33	76.0	0.24	11.1
	Dec	11872	128.8	0.14	79.6	0.11	2411	125.0	0.33	75.9	0.23	6.3
2014	Jan	12518	129.1	0.13	79.8	0.10	2519	125.2	0.32	76.0	0.23	4.1

For peer review only

Feb	12500	128.5	0.13	79.3	0.10	2481	124.5	0.32	75.6	0.23	4.6
Mar	13513	128.1	0.12	79.1	0.10	2689	124.3	0.31	75.3	0.22	8.4
Apr	13553	127.0	0.12	78.5	0.10	2660	123.1	0.31	74.5	0.22	12.8
May	13993	125.2	0.12	77.3	0.10	2778	121.4	0.30	73.2	0.22	17.7
Jun	14008	124.1	0.12	76.5	0.10	2695	120.2	0.30	72.1	0.21	21.5
Jul	13855	123.6	0.12	76.1	0.10	2710	119.3	0.30	71.7	0.22	24.9
Aug	13953	124.5	0.12	76.7	0.10	2730	120.1	0.30	72.1	0.22	25.4
Sep	14328	125.3	0.12	77.5	0.10	2837	121.0	0.30	73.2	0.22	21.6
Oct	14614	126.7	0.12	78.5	0.09	2902	123.4	0.30	75.0	0.21	17.2
Nov	14592	128.0	0.12	79.4	0.10	2816	125.0	0.32	76.0	0.22	12.4
Dec	14392	128.9	0.12	79.7	0.10	2761	125.8	0.32	76.4	0.23	5.2

For peer review only

2015	Jan	15193	129.0	0.12	79.7	0.09	3020	125.1	0.30	75.9	0.21	4.5
	Feb	15374	128.7	0.12	79.7	0.09	3001	124.9	0.30	76.0	0.21	4.8
	Mar	15975	128.0	0.11	79.3	0.09	3183	123.9	0.29	75.6	0.21	8.6
	Apr	16447	126.7	0.11	78.5	0.09	3258	123.3	0.29	75.2	0.20	13.4
	May	17167	125.1	0.11	77.5	0.09	3394	120.7	0.27	73.5	0.19	18.7
	Jun	17282	124.7	0.11	77.4	0.09	3452	120.4	0.27	73.4	0.20	20.5
	Jul	17143	124.1	0.11	76.9	0.09	3306	119.4	0.27	72.8	0.21	24.6
	Aug	16891	124.9	0.11	77.5	0.09	3317	120.4	0.27	73.3	0.20	25.4
	Sep	16927	125.4	0.11	78.1	0.09	3378	121.3	0.27	74.1	0.20	21.3

BP: blood pressure, C: degree Celsius, SE: Standard error, Temp: temperature, Jan: January, Feb: February, Mar: March, Apr: April, Jun: June, Jul: July, Aug: August, Sep: September, Oct: October, Nov: November, Dec: December

For peer review only

Supplementary Table 4. Monthly means (and standard errors) of home morning blood pressure by age groups (ages less than 60 years) from September 2013 to September 2015.

		Men					Women					Temp
		N	Systolic BP [mmHg]		Diastolic BP [mmHg]		N	Systolic BP [mmHg]		Diastolic BP [mmHg]		[C]
			Mean	SE	Mean	SE		Mean	SE	Mean	SE	
2013	Sep	9546	130.2	0.14	84.7	0.11	1706	122.1	0.39	77.5	0.29	22.1
	Oct	9972	131.7	0.14	85.7	0.11	1782	124.7	0.39	79.4	0.28	17.7
	Nov	10003	134.0	0.14	87.1	0.11	1748	126.5	0.38	80.6	0.28	10.0
	Dec	10054	134.6	0.14	87.3	0.11	1736	127.0	0.38	81.0	0.28	5.1
2014	Jan	10461	134.3	0.14	87.0	0.10	1808	126.8	0.37	80.9	0.28	2.7

For peer review only - http://bmjopen.bmj.com/site/about/guidelines.xhtml

11

Feb	10570	134.1	0.14	86.8	0.10	1812	126.0	0.37	80.4	0.28	3.1
Mar	11211	133.4	0.13	86.5	0.10	1915	125.2	0.35	79.7	0.26	7.0
Apr	11556	132.7	0.13	86.1	0.10	1971	124.2	0.34	79.3	0.26	11.3
May	11925	131.0	0.12	85.2	0.10	2079	122.6	0.33	78.2	0.25	17.2
Jun	11961	129.8	0.13	84.5	0.10	2111	121.6	0.33	77.3	0.25	21.2
Jul	12008	129.2	0.12	84.1	0.10	2119	120.9	0.34	76.7	0.26	24.4
Aug	11991	129.5	0.12	84.3	0.10	2129	121.0	0.34	76.9	0.26	25.3
Sep	12338	131.1	0.13	85.6	0.10	2254	122.6	0.33	78.2	0.25	20.7
Oct	12464	132.4	0.12	86.4	0.09	2245	124.8	0.33	79.7	0.25	16.2
Nov	12421	133.5	0.12	87.0	0.09	2174	126.0	0.34	80.6	0.26	11.2
Dec	12112	135.0	0.13	87.8	0.10	2097	127.4	0.35	81.3	0.26	4.4

For peer review only

2015	Jan	12672	134.3	0.12	87.2	0.09	2239	126.1	0.34	80.4	0.24	3.6
	Feb	12922	133.9	0.12	87.0	0.09	2309	125.7	0.33	80.2	0.24	3.5
	Mar	13438	133.3	0.12	86.8	0.09	2451	125.4	0.32	80.3	0.23	7.2
	Apr	14019	132.0	0.11	86.1	0.09	2568	124.0	0.32	79.5	0.23	12.7
	May	14879	130.6	0.11	85.3	0.08	2712	122.8	0.30	78.8	0.22	18.2
	Jun	15186	130.0	0.11	85.0	0.08	2826	122.0	0.29	78.5	0.22	20.3
	Jul	15141	129.2	0.11	84.5	0.08	2785	121.1	0.30	77.8	0.23	24.3
	Aug	14955	129.7	0.11	84.9	0.08	2816	121.4	0.30	77.9	0.22	25.1
	Sep	14986	130.6	0.11	85.8	0.08	2844	122.3	0.30	78.9	0.21	20.9

BP: blood pressure, C: degree Celsius, SE: Standard error, Temp: temperature, Jan: January, Feb: February, Mar: March, Apr: April, Jun: June, Jul: July, Aug: August, Sep: September, Oct: October, Nov: November, Dec: December

For peer review only

Supplementary Table 5. Monthly means (and standard errors) of home morning blood pressure by age groups (ages 60 years or older) from September 2013 to September 2015.

		Men					Women					Temp
		N	Systolic BP [mmHg]		Diastolic BP [mmHg]		N	Systolic BP [mmHg]		Diastolic BP [mmHg]		[C]
			Mean	SE	Mean	SE		Mean	SE	Mean	SE	
2013	Sep	3638	129.7	0.22	78.0	0.15	857	126.7	0.49	74.2	0.31	22.1
	Oct	3807	131.9	0.22	79.3	0.15	899	129.3	0.48	75.9	0.31	17.7
	Nov	3875	134.6	0.22	80.7	0.15	896	132.6	0.50	77.6	0.32	10.0
	Dec	3943	134.6	0.22	80.8	0.15	941	132.5	0.47	77.6	0.30	5.0
2014	Jan	4117	134.7	0.21	80.8	0.15	944	133.0	0.50	77.5	0.31	2.8

For peer review only

Feb	4132	134.1	0.21	80.4	0.15	970	132.3	0.49	77.4	0.31	3.1
Mar	4478	133.1	0.20	80.0	0.14	1039	131.4	0.43	77.0	0.29	7.1
Apr	4622	132.1	0.19	79.5	0.14	1100	130.8	0.45	76.5	0.28	11.4
May	4795	130.2	0.18	78.5	0.13	1123	129.0	0.43	75.2	0.27	17.1
Jun	4926	128.5	0.18	77.5	0.13	1113	126.7	0.41	73.7	0.26	21.2
Jul	4997	127.4	0.18	76.8	0.13	1119	125.1	0.40	72.9	0.27	24.5
Aug	5101	127.6	0.18	77.0	0.13	1132	125.0	0.41	72.9	0.27	25.3
Sep	5190	129.9	0.18	78.5	0.13	1169	127.9	0.43	74.6	0.27	20.7
Oct	5280	131.9	0.18	79.7	0.13	1194	129.8	0.42	76.0	0.26	16.3
Nov	5269	133.0	0.18	80.3	0.13	1204	131.2	0.42	77.0	0.27	11.2
Dec	5270	134.6	0.18	81.0	0.13	1198	132.9	0.44	77.8	0.27	4.3

For peer review only

2015	Jan	5461	134.1	0.18	80.8	0.12	1233	132.2	0.42	77.3	0.26	3.5
	Feb	5561	133.6	0.17	80.7	0.12	1228	131.9	0.43	77.4	0.27	3.5
	Mar	5828	132.7	0.17	80.2	0.12	1289	131.0	0.41	77.1	0.26	7.2
	Apr	6054	131.4	0.16	79.5	0.12	1333	129.4	0.39	76.2	0.25	12.7
	May	6261	130.0	0.16	78.8	0.12	1350	127.5	0.37	74.9	0.24	18.1
	Jun	6326	129.0	0.16	78.4	0.11	1385	126.5	0.36	74.3	0.24	20.3
	Jul	6391	127.3	0.15	77.5	0.11	1327	124.3	0.37	73.0	0.25	24.3
	Aug	6433	128.0	0.15	77.8	0.11	1348	125.8	0.38	73.8	0.25	25.0
	Sep	6422	129.5	0.15	79.0	0.11	1367	127.9	0.38	75.3	0.25	20.9

BP: blood pressure, C: degree Celsius, SE: Standard error, Temp: temperature, Jan: January, Feb: February, Mar: March, Apr: April, Jun: June, Jul: July, Aug: August, Sep: September, Oct: October, Nov: November, Dec: December

For peer review only

STROBE Statement—checklist of items that should be included in reports of observational studies

	Item No	Recommendation	Page/Line Number
**Title and abstract**	1	(*a*) Indicate the study's design with a commonly used term in the title or the abstract	Page 3 / lines 9-18
		(*b*) Provide in the abstract an informative and balanced summary of what was done and what was found	Page 3 / line 2 – Page 4 / line 12
**Introduction**			
Background/rationale	2	Explain the scientific background and rationale for the investigation being reported	Page 6 / line 2 – Page7 / line 2
Objectives	3	State specific objectives, including any prespecified hypotheses	Page 7 / lines 3 –8
**Methods**			
Study design	4	Present key elements of study design early in the paper	Page 7 / line 9 – Page 10 / line 4
Setting	5	Describe the setting, locations, and relevant dates, including periods of recruitment, exposure, follow-up, and data collection	Page 7 / line 9 – Page 9 / line 13
Participants	6	(*a*) *Cohort study*—Give the eligibility criteria, and the sources and methods of selection of participants. Describe methods of follow-up<br>*Case-control study*—Give the eligibility criteria, and the sources and methods of case ascertainment and control selection. Give the rationale for the choice of cases and controls<br>*Cross-sectional study*—Give the eligibility criteria, and the sources and methods of selection of participants	Page 9 / line 12 – Page 10 / line 1
		(*b*) *Cohort study*—For matched studies, give matching criteria and number of exposed and unexposed<br>*Case-control study*—For matched studies, give matching criteria and the number of controls per case	NA
Variables	7	Clearly define all outcomes, exposures, predictors, potential confounders, and effect modifiers. Give diagnostic criteria, if applicable	Page 10 / lines 2 – 14
Data sources/ measurement	8*	For each variable of interest, give sources of data and details of methods of assessment (measurement). Describe comparability of assessment methods if there is more than one group	Page 7 / line 9 – Page 10 / line 7
Bias	9	Describe any efforts to address potential sources of bias	Page 8 / lines 9 – 13, Page 10 / line 15 – Page 11 / line 1
Study size	10	Explain how the study size was arrived at	Page 9 / line 12 – Page 10 / line 1
Quantitative variables	11	Explain how quantitative variables were handled in the analyses. If applicable, describe which groupings were chosen and why	NA
Statistical methods	12	(*a*) Describe all statistical methods, including those used to control for confounding	Page 10 / lines 8 – 14
		(*b*) Describe any methods used to examine subgroups and interactions	Page 10 / lines 8 – 10
		(*c*) Explain how missing data were addressed	Page 9 / line 12 – Page 10 / line 1
		(*d*) *Cohort study*—If applicable, explain how loss to follow-up was addressed<br>*Case-control study*—If applicable, explain how matching of cases and controls was addressed<br>*Cross-sectional study*—If applicable, describe analytical methods taking account of sampling strategy	Page 9 / line 12 – Page 10 / line 14

For peer review only

		(*e*) Describe any sensitivity analyses	Page 10 / lines 8-10
**Results**			
Participants	13*	(a) Report numbers of individuals at each stage of study—eg numbers potentially eligible, examined for eligibility, confirmed eligible, included in the study, completing follow-up, and analysed	Page 11 / lines 4 – 6
		(b) Give reasons for non-participation at each stage	NA
		(c) Consider use of a flow diagram	NA
Descriptive data	14*	(a) Give characteristics of study participants (eg demographic, clinical, social) and information on exposures and potential confounders	Page 11 / lines 4-6, Supplemental Table 1
		(b) Indicate number of participants with missing data for each variable of interest	NA
		(c) *Cohort study*—Summarise follow-up time (eg, average and total amount)	NA
Outcome data	15*	*Cohort study*—Report numbers of outcome events or summary measures over time	NA
		Case-control study—Report numbers in each exposure category, or summary measures of exposure	NA
		Cross-sectional study—Report numbers of outcome events or summary measures	Supplemental Table 2-5
Main results	16	(*a*) Give unadjusted estimates and, if applicable, confounder-adjusted estimates and their precision (eg, 95% confidence interval). Make clear which confounders were adjusted for and why they were included	Page 11 / lines 8-16, Supplemental Table 1-5
		(*b*) Report category boundaries when continuous variables were categorized	NA
		(*c*) If relevant, consider translating estimates of relative risk into absolute risk for a meaningful time period	NA
Other analyses	17	Report other analyses done—eg analyses of subgroups and interactions, and sensitivity analyses	NA
**Discussion**			
Key results	18	Summarise key results with reference to study objectives	Page 14 / lines 12-18
Limitations	19	Discuss limitations of the study, taking into account sources of potential bias or imprecision. Discuss both direction and magnitude of any potential bias	Page 15 / line 18– Page 16 / line 6
Interpretation	20	Give a cautious overall interpretation of results considering objectives, limitations, multiplicity of analyses, results from similar studies, and other relevant evidence	Page 14 / line 19 – Page 16 / line 14
Generalisability	21	Discuss the generalisability (external validity) of the study results	Page 16 / lines 7–14
**Other information**			
Funding	22	Give the source of funding and the role of the funders for the present study and, if applicable, for the original study on which the present article is based	Page 21 / lines 5-16

*Give information separately for cases and controls in case-control studies and, if applicable, for exposed and unexposed groups in cohort and cross-sectional studies.

Note: An Explanation and Elaboration article discusses each checklist item and gives methodological background and published examples of transparent reporting. The STROBE checklist is best used in conjunction with this article (freely available on the Web sites of PLoS Medicine at http://www.plosmedicine.org/, Annals of Internal Medicine at http://www.annals.org/, and Epidemiology at http://www.epidem.com/). Information on the STROBE Initiative is available at www.strobe-statement.org.

For peer review only

Seasonal variation in home blood pressure: findings from nationwide web-based monitoring in Japan

Journal:	*BMJ Open*
Manuscript ID	bmjopen-2017-017351.R2
Article Type:	Research
Date Submitted by the Author:	06-Oct-2017
Complete List of Authors:	Iwahori, Toshiyuki; OMRON HEALTHCARE Co., Ltd, ; SHIGA UNIVERSITY of MEDICAL SCIENCE, HEALTH SCIENCE<br>Miura, katsuyuki; Shiga University of Medical Science, Health Science<br>Obayashi, Keiichi; Omron Kabushiki Kaisha<br>Ohkubo, Takayoshi<br>Nakajima, Hiroshi; Omron Kabushiki Kaisha<br>Shiga, Toshikazu; OMRON HEALTHCARE Co., Ltd.,<br>Ueshima, Hirotsugu; Shiga University of Medical Science, Health Science
**Primary Subject Heading**:	Public health
Secondary Subject Heading:	Public health, Cardiovascular medicine
Keywords:	PUBLIC HEALTH, Hypertension < CARDIOLOGY, EPIDEMIOLOGY

SCHOLARONE™
Manuscripts

For peer review only

Title:

Seasonal variation in home blood pressure: findings from nationwide web-based monitoring in Japan

Authors:

Toshiyuki Iwahori, PhD [1,2*], Katsuyuki Miura, MD [1,3], Keiichi Obayashi, MSE [4], Takayoshi Ohkubo, MD [5], Hiroshi Nakajima, PhD [4], Toshikazu Shiga, PhD [2], Hirotsugu Ueshima, MD [1,3]

Affiliations:

[1] Department of Public Health, Shiga University of Medical Science, Otsu, Japan,

[2] OMRON Healthcare Co., Ltd., Muko, Japan,

[3] Center for Epidemiologic Research in Asia, Shiga University of Medical Science, Otsu, Japan,

[4] OMRON Corporation, Kizugawa, Japan,

[5] Department of Hygiene and Public Health, Teikyo University School of Medicine, Tokyo, Japan

For peer review only

Full address:

[1] Seta Tsukinowa-cho, Otsu, Shiga, 520-2192, Japan,

[2] 53, Kunotsubo, Terado-cho, Muko, Kyoto, 617-0002, Japan,

[3] Seta Tsukinowa-cho, Otsu, Shiga, 520-2192, Japan,

[4] 9-1 Kizugawadai, Kizugawa, Kyoto, 619-0283, Japan,

[5] 2-11-1 Kaga, Itabashi-ku, Tokyo, 173-8605, Japan

Corresponding author: Toshiyuki Iwahori, PhD

Department of Public Health

Shiga University of Medical Science

Seta Tsukinowa-cho, Otsu, Shiga 520 2192, Japan

Tel.: +81 77 548 2191

Fax: +81 77 543 9732

E-mail address: iwahori@belle.shiga-med.ac.jp

For peer review only

Abstract

Objectives: Our aim was to assess seasonal variation in home blood pressure (BP) among free-living nationwide users using home BP values accumulated from web-based health care platform established in Japan.

Settings: An observational study. OMRON Healthcare has been developing web-based personal health care record systems in Japan since November 2010; over two million voluntary participants had joined this platform in September 2015. Nationwide home BP measured by oscillometric-type electronic sphygmomanometers from over 110 thousand voluntary participants have been collected to the system from devices.

Participants: Seasonal variation in home BP was evaluated among 64,536 (51,335 men, 13,201 women; mean age, 52.9 years) free-living nationwide users whom automatically and simultaneously transmitted data to the system from devices.

Primary outcome measures: Mean monthly and weekly home BP

Results: In multiple regression analysis, the relationship between BP and temperature was a significant inverse association, independent of age, gender and geological locations. Highest and lowest BP was observed in December and July, respectively. Substantial seasonal differences in the mean values of morning and evening home systolic BP between summer and winter were 6.2 mmHg and 5.5 mmHg in men, and 7.3 mmHg and 6.5 mmHg in women. Seasonal variation was a little larger in older (7.3

For peer review only

mmHg in men, 8.7 mmHg in women) than in younger individuals (5.8 mmHg in men, 6.5 mmHg in women). BP from February to July showed approximately 1.5 mmHg lower than the value from August to December.

Conclusions: Web-based healthcare platform has enabled easier monitoring of population-wide BP. Tighter BP control is necessary in winter than in summer, and especially in colder climate toward winter than toward summer. New technologies using web-based self-monitoring systems for health-related indexes are expected to initiate a new phase of cardiovascular disease prevention, and public health promotion.

(293/300 words)

For peer review only

Strengths and limitations of this study

- Frequent nationwide home BP monitoring by the web-based platform enabled to see seasonal variation of home BP in monthly and weekly changes in large sample (>60,000) from non-medical settings.
- Nationwide home BP data automatically collected and electronically transmitted simultaneously to the web-based home BP monitoring platform minimized the selection bias and reflected the casual BP values in the real world.

- The majority of the participants might have been health conscious individuals.
- Timing and frequency of the measurement, and the room temperature during home BP measurements remain unknown.

For peer review only

Introduction

The management of blood pressure (BP) is one of the most important strategies for the prevention, and management of cardiovascular diseases (CVD) around the world.[1–6] Although BP management has been generally based on BP values measured in the clinical setting by medical professionals, home BP monitoring by individuals is becoming increasingly important in diagnosis, and control of arterial hypertension.[4–9]

Electronic devices for home BP measurement were developed in the 1990s. Companies that manufacture devices for home BP measurement tend to concentrate in the East Asian region (Japan, South Korea, and Taiwan). Japan is unique for its high coverage of home BP measurement; nearly 20 million devices were estimated to exist out of approximately 50 million households by the sales record of BP monitors sold by OMRON Healthcare (Kyoto, Japan) in 2012.

Under these conditions, OMRON Healthcare, a health care device manufacturer, developed a web-based health care platform in 2010 that consumers can join voluntarily after purchasing one of its products. OMRON Healthcare's recently developed devices for home BP measurement send all measured values to a web-based database electronically via personal computers, or mobile phones. From 2010 to 2015, more than two million people in Japan have voluntarily participated in this web-based health care platform. Nowadays, these newly established technologies and platforms enable to

For peer review only

collect real world data simultaneously and show the possibilities of lowering the hurdles to detect useful population-wide information and provide new approach for public health authorities.

Multiple observational studies have examined the seasonal variation of BP in clinical settings; [10] however, there is no report on seasonal variation using nationwide large sample of home BP monitoring data from non-medical and uncontrolled settings. Thus, our aims were to assess seasonal variation in home blood pressure among free-living nationwide users by real world data collection by this web-based health care platform, and to see the applications for public health policy making.

Materials and Methods

Web-based personal healthcare record systems

OMRON Healthcare has been developing web-based personal health care record systems. One of its application services is named as WellnessLINK, which was deployed on November 1st, 2010. The participants can register and leave the web based healthcare platform by their freewill in anytime. WellnessLINK utilizes many kinds of health care devices connected to the internet via personal computers, and mobile phones to collect various types of healthcare data into a database.

For peer review only

The WellnessLINK system records BP values measured at home using oscillometric-type electronic sphygmomanometers, including an upper arm BP monitor (HEM-7510C, HEM-7500F, HEM-7324C, HEM-7320F, HEM-7280C, HEM-7270C, HEM-7250-IT; OMRON Healthcare), an upper arm BP monitor with an automatic cuff wrapping system (HEM-1025; OMRON Healthcare), and a wrist BP monitor (HEM-6321T, HEM-6320T, HEM-6310F, HEM-6300F; OMRON Healthcare). These devices can be share used with family members by differentiating the user identification numbers set at each device among family members. All devices have been validated and approved by the Ministry of Health, Labour and Welfare, Japan [11], and certified as having been adjusted to the ISO/IEC 81060-2 standards [12]. The users receive an instruction manual for BP measurement based on the Japanese Society of Hypertension Guidelines for Self-Monitoring of Blood Pressure at Home.[7] Users then access the website via their personal computer, or mobile phone, and register after approving a privacy policy agreement. For personal computer users, BP data are transmitted to the system via internet by connecting a sphygmomanometer using either by Universal Serial Bus (USB), Near Field Communication (NFC), Bluetooth, or WiFi. For mobile phone users, BP data are transmitted to the system via the internet through either NFC, or Bluetooth. In most of the devices, an active data update is required to transmit the data automatically from the devices to the system by the individuals. However, some

For peer review only

recent devices with WiFi connections transfer data both simultaneously, and automatically to the system. Self-reported data, manually inputted by a user, is also accepted. All BP data from users who voluntarily register with this service are sent to the computer server at OMRON Healthcare.

The WellnessLINK system provides various kinds of health support programs, including antihypertensive health care, metabolic syndrome prevention, dietary improvement, and physical activity. In the antihypertensive health care program, WellnessLINK provides personalized daily, weekly, monthly, and yearly graphs of BP, which are useful for individual BP management. In addition, feedback is provided to the users after accumulated data is analyzed.

Blood pressure data analysis

A total of 2,164,321 individuals in Japan registered in the WellnessLINK system by the end of September 2015. Among these users, 110,454 sent BP data to the system automatically from BP measurement devices, or manually. Self-reported data that were manually inputted by users were excluded from the data analysis in this study. In this report, we analyzed 41.7 million BP measurement data from 64,536 registered users (51,335 men, 13,201 women; mean age, 52.9 years) who sent their all measured BP data

For peer review only

automatically. Among these users, 24% were treated with anti-hypertensive medication.

BP values measured at home between 4:00 AM, and 9:59 AM were defined as "morning BP," while those measured at home between 7:00 PM, and 1:59 AM were defined as "evening BP." Individual mean morning BP values were calculated using the first measured BP value in the morning, and measured within ten minutes later. Individuals mean evening BP values were calculated using the last measured BP value in the evening, and values measured within ten minutes previously. The monthly means of home BP was calculated by averaging individuals' home BP of overall participants.

Descriptive statistics values such as mean monthly systolic and diastolic BPs (and standard errors) were calculated by sex, and age groups for two years, from September 2013 to September 2015. Monthly mean BP changes for two years were shown in figures along with mean outdoor temperatures. Intermonth differences in BP were assessed analysis of variance. The t test for unpaired observations was used to determine the significance of the difference, after Bonferroni correction for multiple comparisons. Multiple regression analysis was preformed to assess whether the association between temperature and BP is independent of age (continuous variable), gender (dichotomous: men:1, women: 0) and geological locations (Northern Japan / Eastern Japan / Central Japan / Kansai region / Western Japan). Four dummy variables for five geological locations were assigned and Eastern Japan was defined as the reference area. All P

For peer review only

values were two sided, and P values < 0.05 was taken as the level of statistical significance. Mean outdoor temperatures were calculated using outdoor temperatures at the time of each measurement in the capital city of the prefecture in which each participant lived, referenced from the database of the Japan Meteorological Agency.

The participants of this study had voluntarily filled out the online consent form, agreed to send their data to the data server managed by OMRON Healthcare to use the WellnessLINK system service, and agreed with the condition that these data obtained by the WellnessLINK system would be analyzed for medical research use after data anonymization. The ethics committee of the Omron Healthcare Company approved the study protocol.

Results

Mean monthly morning and evening home systolic blood pressure

A total of 64,536 Japanese registered users sent their all measured BP data automatically from September 2013 to September 2015 from nationwide (Supplementary Table 1). Approximately 83% of the study participants were men, mean age was 54 years old, and the treatment rate among hypertensive people in the present study was approximately 39%; however these status remained relatively stable throughout the study period. Mean monthly morning and evening home systolic BP

For peer review only

(SBP), as well as mean monthly temperatures for a two-year period, are shown in Figure 1. Seasonal variation was apparent in both morning, and evening home SBP in both sexes; values were higher in the winter, and lower in the summer (Figure 1, Supplemental Table 2-3, Supplemental Movie). The relationship between home SBP and temperature showed a significant inverse association which was independent of age, gender and geological locations (Table 1). Age, gender and geographic locations had no significant interactions on the relationship between BP and temperature (all P>0.05). The maximum winter-summer difference was a little larger for morning SBP (6.2 mmHg in men, 7.3 mmHg in women) compared with evening SBP (5.5 mmHg in men, 6.5 mmHg in women). Similar seasonal variation was observed in morning SBP for younger, and older individuals (Figure 2, Supplemental Table 4-5), with the winter-summer difference being a little larger in older (7.3 mmHg in men, 8.7 mmHg in women) than in younger individuals (5.8 mmHg in men, 6.5 mmHg in women).

The amplitude of winter-summer difference was smaller in diastolic BP compared to SBP; however, similar trend of seasonal variation was observed in diastolic BP (Supplemental Table 2-5).

Monthly morning home systolic blood pressure and mean outdoor temperatures

For peer review only

The association between mean monthly morning home SBP and mean outdoor temperatures for a 1-year period in men was skewed and showed hysteresis loop characteristic (Figure 3 and Supplemental Table 2). Outside temperature was lowest in February (3.5°C) but similar from December (4.4°C) to February; however, morning home SBP lowered toward February (134.8 mmHg to 133.8 mmHg) from the highest recorded in December. Furthermore, outside temperature was highest in August (25.1°C) but similar from July (24.3°C) to August; however, morning home SBP was higher in August (129.2 mmHg) than the lowest recorded in July (128.6 mmHg). Morning home SBP from February to July showed approximately 1.5mmHg lower than the value from August to December (e.g., morning home SBP was 1.5 mmHg higher in November [11.2°C] than in April [12.7°C]). There were statistical significances observed in BP between summer and winter months; however, there were a few intermonth combinations which did not reach statistical significance (March versus November, and May versus September). Similar findings were observed in evening home SBP and DBP, regardless of gender and age subgroups (Supplemental Table 2-5).

Discussion

The association between BP and temperature was inverse (winter-summer

For peer review only

difference: approximately 7 mmHg) and independent of other factors such as age, gender and geological locations. In multiple regression analysis, SBP fell by 0.27 mmHg for each 1°C increase.

Monitoring population-wide BP is important for public health, especially for CVD prevention.[1-6] Previous studies have shown approximately 2–10 mmHg, 4–8 mmHg, and 3 mmHg of seasonal variability in clinic BP, home BP, and 24-hour ambulatory BP (ABP), respectively.[13-22] Most clinics are temperature controlled; thus, likely do not represent daily climate conditions.[15,23] Seasonal variation in CVD incidence has been reported,[24–29] and seasonal variation in BP has been suggested to be associated with seasonal variation in the incidence of CVD.[23] Stamler et al. estimated the reduction in population BP for 2-5 mmHg deliver reduction in mortality of stroke, mortality of coronary heart disease and total mortality for approximately 6-14%, 4-9% and 3-7%, respectively [30]. However, there was no system available for public health authorities to detect population-wide BP to plan out countermeasures, and announce alert for CVD prevention. Thus, acquiring nationwide "real world" home BP data is desirable.

The rapid diffusion of home BP monitoring has been aided by technological progress, the wider availability of measurement devices, increased awareness of the importance of regular BP monitoring, and recognition of the usefulness of home BP

For peer review only

measurement by international hypertension management guidelines.[4–9] The National Health and Nutrition Survey of Japan showed that in 2010, 46% of adults, and 72% of hypertensive patients had measured their BP at home within the previous year.[31] Therefore, devices for home BP measurement have been spread nationwide, have been used more frequently in Japan than in any other country in the world. However, conventional data collection methods using self-recorded home BP may result in a bias, mainly underreported BP values.[7,32] The uniqueness of the system in the present study is that nationwide home BP data are automatically collected, and electronically transmitted simultaneously to the web-based home BP monitoring platform. This system enables the elimination of manual processes performed with substantial effort by clinical staff, and minimizes the possibility of a selection bias in the data collection. Thus, we demonstrated the practical use of web-based healthcare platform which may reliably reflect the trend of population-wide BP as a "real world" data here in weekly and monthly analyses.

Findings from other previous studies suggest that outdoor temperature, and home central heating affect the seasonal variability of BP, especially during the winter.[16,22-23] In the present study, a parallel association was observed between seasonal variation in home BP (about 7 mmHg) and changes in outdoor temperature in this "real world" data. This finding was consistent with previous report with smaller sample sized, lower

For peer review only

frequent and manually collected data.[21] One interesting finding was that home BP tended higher for about 1.5 mmHg during autumn (summer to winter) than in spring (winter to summer). To the best of our knowledge, we are the first to report this hysteresis loop characteristic. Tighter BP control may be required during autumn than in spring.

Ideally, public health authorities would be able to notice population-wide changes in BP through monitoring population-wide "real world" home BP data by using this platform, and this would allow them to forecast CVD risk at appropriate times. Furthermore, public health authorities may also make announcements for clinicians, and practitioners to be cautious of over- and under-dose of anti-hypertensive medication by referencing the population-wide "real world" BP which was easily screened for appropriate BP control. Such new approaches may initiate new phases for CVD prevention and public health promotion. In addition to devices for home BP measurement, adding varieties of lifestyle monitoring devices related to BP management (e.g., monitors for body weight, physical activity [33], sleep [34], body temperature, and urinary sodium-to-potassium ratio [35]) to this platform may support medical and public health experts to identify new realities, assess the concurrent population-wide CVD risk and plan out appropriate countermeasures. Therefore, by using this platform, much appropriate feedbacks from public health authorities could be

For peer review only

expected to support individuals to take right action toward healthy lifestyle.

To the best of our knowledge, this is the first study to report seasonal variation from a large sample of “real world” home BP data from non-medical settings. In this study, the participants were whom registered to use this web based healthcare platform; the participants might have higher socioeconomic status since they may have higher computer literacy and much health conscious people compared to others in general populations. Therefore, the seasonal variation of the “real world” home BP might have been underestimated due to the higher socioeconomic status of the study participants. Moreover, the participants in this web based healthcare platform can register and leave by their freewill in anytime, this may aggravates the selection bias. However, the large sample size (>60,000), voluntary participation in the healthcare platform, measurement by a diverse range of individuals, various measurement times, including weekends, and non-clinical measurement settings are thought to minimize the possibility of selection bias in this study, and reflect casual BP values in the real world.

This study has some limitation. As the data obtained during the study was “real world” data, the room temperature during home BP measurements was unknown, some of the BP measurement devices might have been used by multiple users without differentiating the user identification numbers and the frequency and timing of BP measurements may have differed for each individual.

For peer review only

Based on the findings in this study, combining current public health policies with emerging technologies could be expected to help authorities monitor health-related indexes. To reduce CVD incidents, and for CVD prevention, we need to piece out the situation of individuals of entire population. Therefore, establishing web-based healthcare platform by public health authorities might be an alternative for obtaining eyes for active control of public health outcome. Initiation of a new phase for both CVD prevention and public health promotion may contribute to reduce nationwide medical expenditures.

(2625/3000words)

For peer review only

References

1. Lewington S, Clarke R, Qizilbash N, Peto R, Collins R; Prospective Studies Collaboration. Age-specific relevance of usual blood pressure to vascular mortality: a meta-analysis of individual data for one million adults in 61 prospective studies. *Lancet*. 2002 Dec 14;**360**(9349):1903-13.

2. Xie X, Atkins E, Lv J, et al. Effects of intensive blood pressure lowering on cardiovascular and renal outcomes: updated systematic review and meta-analysis. *Lancet*. 2016 Jan 30;**387**(10017):435-43.

3. Jaffe MG, Young JD. The Kaiser Permanente Northern California Story: Improving Hypertension Control From 44% to 90% in 13 Years (2000 to 2013). *J Clin Hypertens (Greenwich)*. 2016 Apr;18(4):260-1.

4. Mancia G, Fagard R, Narkiewicz K, et al. 2013 ESH/ESC guidelines for the management of arterial hypertension: the Task Force for the Management of Arterial Hypertension of the European Society of Hypertension (ESH) and of the European Society of Cardiology (ESC). *Blood Press* 2013; **22**: 193–278.

5. Armstrong C. JNC8 guidelines for the management of hypertension in adults. *Am Fam Physician*. 2014 Oct 1;**90**(7):503-4.

6. Shimamoto K, Ando K, Fujita T, et al. The Japanese Society of Hypertension Guidelines for the Management of Hypertension (JSH 2014). *Hypertens Res.* 2014 Apr;**37**(4):253-390. doi: 10.1038/hr.2014.20.

7. Imai Y, Kario K, Shimada K, et al. The Japanese Society of Hypertension Guidelines for Self-monitoring of Blood Pressure at Home (Second Edition). *Hypertens Res*. 2012 Aug;**35**(8):777-95. doi: 10.1038/hr.2012.56.

8. Parati G, Stergiou GS, Asmar R, et al. European Society of Hypertension guidelines for blood pressure monitoring at home: a summary report of the Second International Consensus Conference on Home Blood Pressure Monitoring. *J Hypertens.* 2008 Aug;**26**(8):1505-26.

9. Stergiou GS, Asayama K, Thijs L, et al. Prognosis of white-coat and masked

For peer review only

hypertension: International Database of HOme blood pressure in relation to Cardiovascular Outcome. *Hypertension*. 2014 Apr;63(4):675-82.

10. Shirasaki O, Terada H, Niwano K, Nakanishi T, Kanai M, Miyawaki Y, Souma T, Tanaka T, Kusunoki T. The Japan Home-health Apparatus Industrial Association: investigation of home-use electronic sphygmomanometers. *Blood Press Monit*. 2001 Dec;6(6):303-7.

11. INTERNATIONAL STANDARD ISO/IEC 81060-2: Non-invasive sphygmomanometers –Part 2: Clinical investigation of automated measurement type. ISO Copyright office, Geneva, Switzerland, 2013

12. Hanazawa T, Asayama K, Watabe D, Hosaka M, Satoh M, Yasui D, Obara T, Inoue R, Metoki H, Kikuya M, Imai Y, Ohkubo T. Seasonal variation in self-measured home blood pressure among patients on antihypertensive medications: HOMED-BP study. *Hypertens Res*. 2016 Sep 29. doi: 10.1038/hr.2016.133.

13. Rose G. Seasonal variation in blood pressure in man. *Nature.* 1961 Jan 21;**189**:235.

14. Brennan PJ, Greenberg G, Miall WE, Thompson SG. Seasonal variation in arterial blood pressure. *Br Med J (Clin Res Ed).* 1982 Oct 2;**285**(6346):919-23.

15. Madsen C, Nafstad P. Associations between environmental exposure and blood pressure among participants in the Oslo Health Study (HUBRO). *Eur J Epidemiol*. 2006;**21**(7):485-91.

16. Lewington S, Li L, Sherliker P, et al. Seasonal variation in blood pressure and its relationship with outdoor temperature in 10 diverse regions of China: the China Kadoorie Biobank. *J Hypertens.* 2012 Jul;**30**(7):1383-91.

17. Modesti PA, Morabito M, Bertolozzi I, et al. Weather-related changes in 24-hour blood pressure profile: effects of age and implications for hypertension management. *Hypertension*. 2006 Feb;**47**(2):155-61.

18. Barnett AG, Sans S, Salomaa V, Kuulasmaa K, Dobson AJ; WHO MONICA Project. The effect of temperature on systolic blood pressure. *Blood Press Monit.* 2007 Jun;**12**(3):195-203.

For peer review only

19. Sega R, Cesana G, Bombelli M, et al. Seasonal variations in home and ambulatory blood pressure in the PAMELA population. Pressione Arteriose Monitorate E Loro Associazioni. *J Hypertens.* 1998 Nov;**16**(11):1585-92.

20. Alpérovitch A, Lacombe JM, Hanon O, et al. Relationship between blood pressure and outdoor temperature in a large sample of elderly individuals: the Three-City study. *Arch Intern Med.* 2009 Jan 12;**169**(1):75-80.

21. Imai Y, Munakata M, Tsuji I, et al. Seasonal variation in blood pressure in normotensive women studied by home measurements. *Clin Sci (Lond).* 1996 Jan;**90**(1):55-60.

22. Murakami S, Otsuka K, Kono T, et al. Impact of outdoor temperature on prewaking morning surge and nocturnal decline in blood pressure in a Japanese population. *Hypertens Res.* 2011 Jan;**34**(1):70-3.

23. Cuspidi C, Ochoa JE, Parati G. Seasonal variations in blood pressure: a complex phenomenon. *J Hypertens.* 2012 Jul;**30**(7):1315-20.

24. Analitis A, Katsouyanni K, Biggeri A, et al. Effects of cold weather on mortality: results from 15 European cities within the PHEWE project. *Am J Epidemiol.* 2008 Dec 15;**168**(12):1397-408.

25. Danet S, Richard F, Montaye M, et al. Unhealthy effects of atmospheric temperature and pressure on the occurrence of myocardial infarction and coronary deaths. A 10-year survey: the Lille-World Health Organization MONICA project (Monitoring trends and determinants in cardiovascular disease). *Circulation.* 1999 Jul 6;**100**(1):E1-7.

26. van Rossum CT, Shipley MJ, Hemingway H, et al. Seasonal variation in cause-specific mortality: are there high-risk groups? 25-year follow-up of civil servants from the first Whitehall study. *Int J Epidemiol.* 2001 Oct;**30**(5):1109-16.

27. Shinkawa A, Ueda K, Hasuo Y, Kiyohara Y, Fujishima M. Seasonal variation in stroke incidence in Hisayama, Japan. *Stroke.* 1990 Sep;**21**(9):1262-7.

For peer review only

28. Sheth T, Nair C, Muller J, Yusuf S. Increased winter mortality from acute myocardial infarction and stroke: the effect of age. *J Am Coll Cardiol.* 1999 Jun;**33**(7):1916-9.

29. Turin TC, Kita Y, Rumana N, et al. Stroke case fatality shows seasonal variation regardless of risk factor status in a Japanese population: 15-year results from the Takashima Stroke Registry. *Neuroepidemiology*. 2009;**32**(1):53-60.

30. Stamler R. Implications of the INTERSALT study. *Hypertension*. 1991 Jan;17(1 Suppl):I16-20.

31. National Institute of Health and Nutrition. The National Health and Nutrition Survey Japan, 2010. Dai-ichi Shuppan Co. Ltd.: Tokyo, Japan, 2013 (in Japanese).

32. Mengden T, Hernandez Medina RM, Beltran B, et al. Reliability of reporting self-measured blood pressure values by hypertensive patients. *Am J Hypertens.* 1998 Dec;**11**(12):1413-7.

33. Tanaka C, Reilly JJ, Tanaka M, Tanaka S. Seasonal changes in objectively measured sedentary behavior and physical activity in Japanese primary school children. *BMC Public Health*. 2016 Sep 13;16:969.

34. Hashizaki M, Nakajima H, Kume K. Monitoring of Weekly Sleep Pattern Variations at Home with a Contactless Biomotion Sensor. *Sensors (Basel)*. 2015 Aug 3;15(8):18950-64.

35. Iwahori T, Ueshima H, Ohgami N, et al. Effectiveness of a self-monitoring device for urinary sodium/potassium ratio on dietary improvement in free-living adults: a randomized controlled trial. *J Epidemiol*. 2017 (in press).

For peer review only

Supplementary Information

Five supplementary tables and one supplementary movie. Supplementary movie shows the nationwide distribution of weekly mean home morning systolic blood pressure from September 2013 to September 2015.

Acknowledgements

This study was conducted by OMRON Healthcare Co., Ltd., and OMRON Corporation in cooperation with the Department of Public Health, Shiga University of Medical Science.

Funding

The running cost of this observational study was funded by OMRON Healthcare Co. Ltd.

Author contributions

KM, TO, HN, TS, HU contributed to the design of the study; KO, HN participated in data collection and data analysis; KM, KO, TI, TO and HU contributed to manuscript drafting; all authors participated in critical revisions of the manuscript; all authors approved the final version of the manuscript for submission.

For peer review only

Declaration of interests

TI and TS are employee of OMRON Healthcare Co., Ltd. KO and HN are employee of OMRON Corporation. Dr. Hirotsugu Ueshima served as a consultant for this project. Dr. Katsuyuki Miura received a research grant from OMRON Healthcare Co., Ltd.

Data sharing statement

No additional data are available.

For peer review only

Figure legends

Figure 1. Monthly means (and standard errors) of (A) home morning and (B) home evening systolic blood pressure (SBP) from September 2013 to September 2015. The maximum winter-summer difference was larger for morning SBP (6.2 mmHg in men, 7.3 mmHg in women) than for evening SBP (5.5 mmHg in men, 6.5 mmHg in women).

Figure 2. Monthly means (and standard errors) of home morning SBP by age groups from September 2013 to September 2015; (A) Participants aged less than 60 years; (B) participants aged 60 years or older. The winter-summer difference was larger in older participants (7.3 mmHg in men, 8.7 mmHg in women) than in younger participants (5.8 mmHg in men, 6.5 mmHg in women).

Figure 3. Association between monthly mean home morning SBP (and standard errors) and mean temperatures in each month from October 2014 to September 2015 in men. Home morning SBP was highest in winter and lowest in summer. Home morning SBP was higher in autumn than in spring, although outdoor temperatures were similar.

For peer review only

Table 1. Regression coefficients for the relationship of home morning systolic blood pressure with age, gender, temperature and geographic locations by multiple regression analysis weighted by numbers of participants in each month.

		Regression coefficient	P value
Age [years]		0.274	0.02
Gender (men versus women)		6.05	<0.001
Temperature [°C]		-0.253	<0.001
Geological locations †			
	Northern Japan †	-0.395	0.054
	Central Japan †	0.186	0.37
	Kansai region †	-0.382	0.08
	Western Japan †	-5.68	<0.001
Adjusted R-squared: 0.9309			

† versus Eastern Japan (Kanto region)

For peer review only

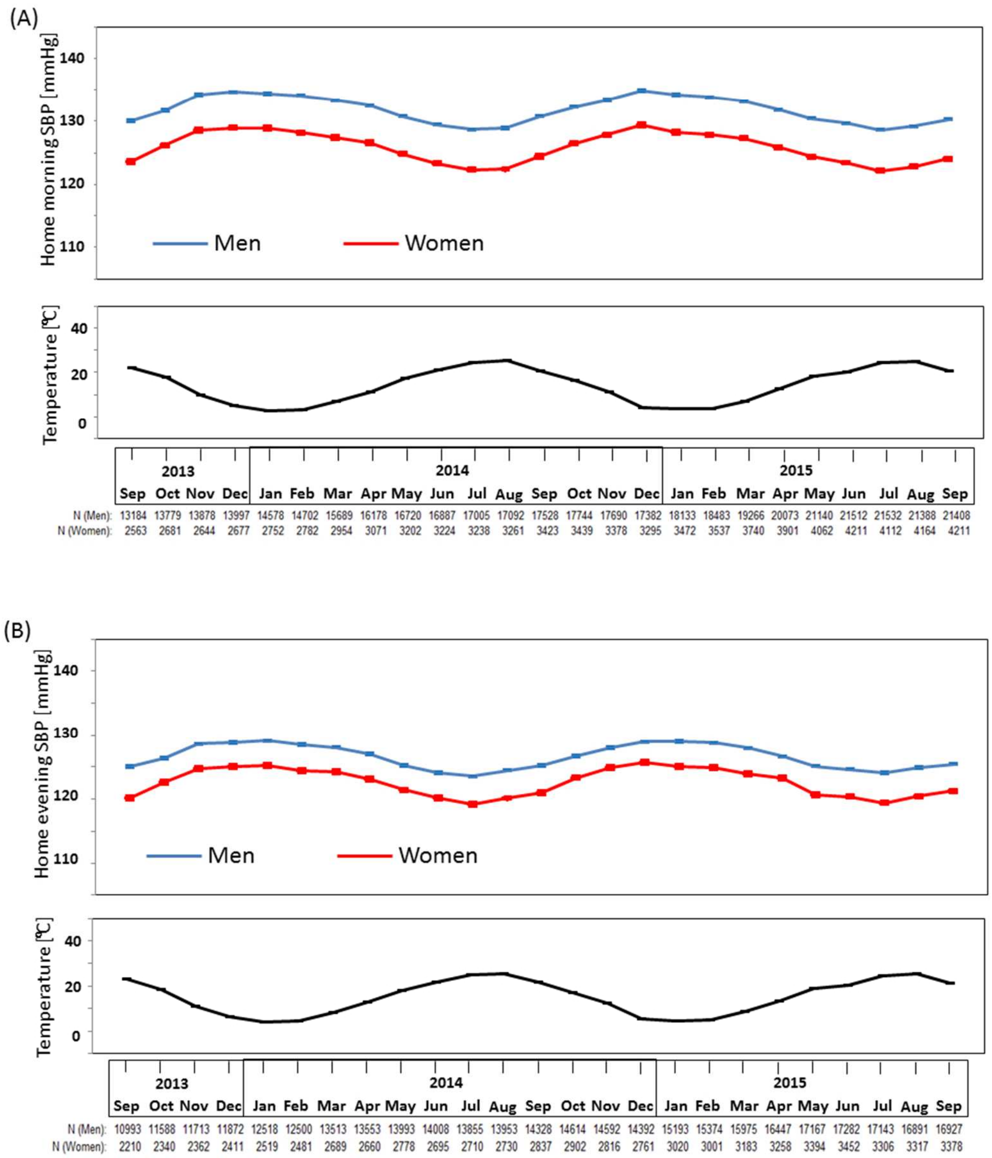

Figure 1

254x296mm (96 x 96 DPI)

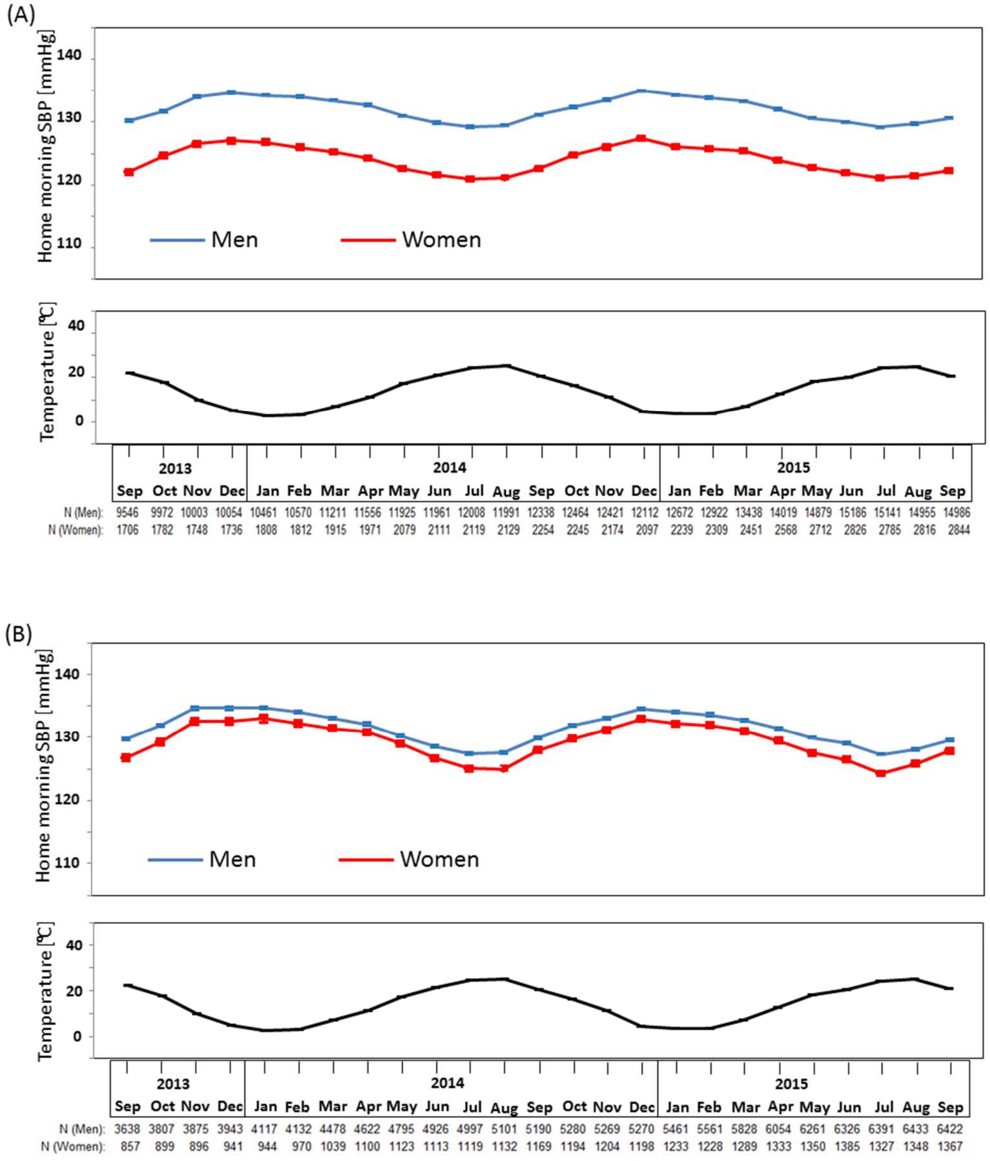

Figure 2

254x297mm (96 x 96 DPI)

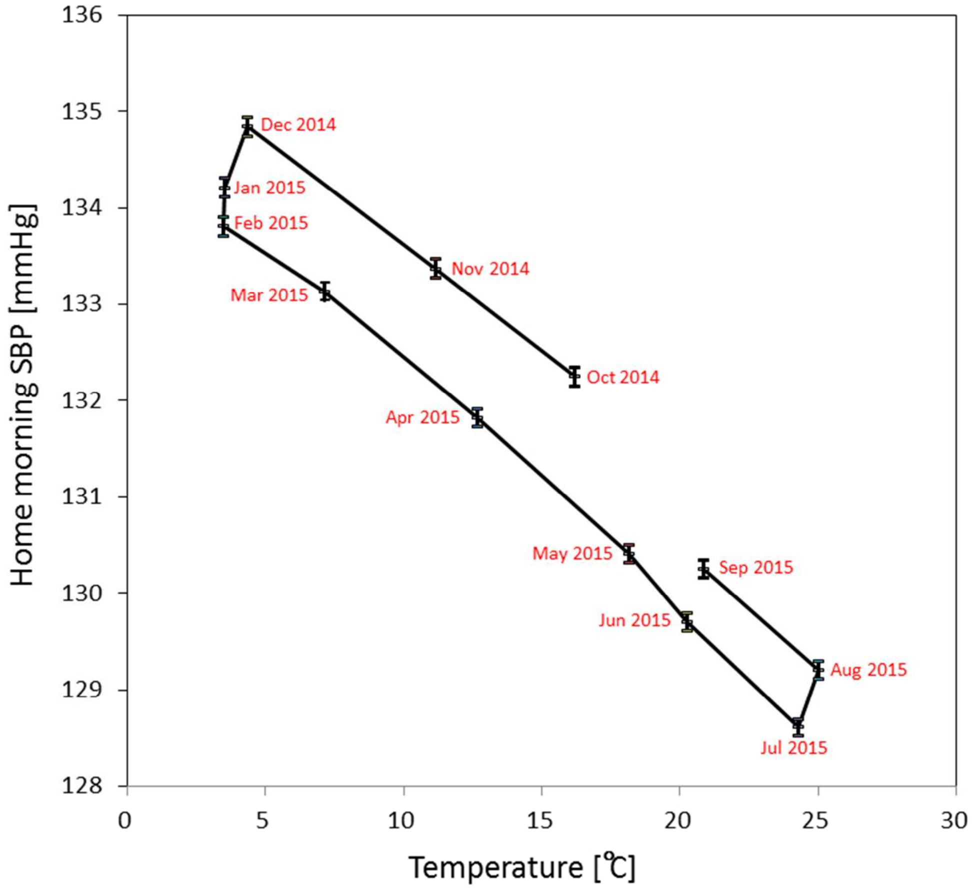

Figure 3

209x190mm (96 x 96 DPI)

Supplementary Table 1. Characteristics of study participants, free living Japanese volunteers, from September, 2013 to September 2015.

Locations (Prefectures)		Men		Women	
		N	Mean age	N	Mean age
Nationwide		51335	51.4	13201	51.0
Northern Japan	Hokkaido	1471	50.6	430	50.4
	Aomori	311	48.2	74	50.8
	Iwate	392	51.9	61	53.3
	Miyagi	846	50.3	266	46.4
	Akita	246	50.7	43	54.5
	Yamagata	286	50.2	56	54.8
	Fukushima	566	52.2	182	56.8
Eastern Japan	Ibaraki	1161	50.9	277	50.2
	Tochigi	753	49.5	156	49.7
	Gunma	613	50.9	143	50.9
	Saitama	3422	51.3	852	49.9
	Chiba	3189	51.2	817	50.7
	Tokyo	9673	50.7	2526	50.2

For peer review only

2

	Kanagawa	6275	51.5	1430	51.0
Central Japan	Niigata	599	50.5	128	50.3
	Toyama	302	51.2	72	50.2
	Ishikawa	351	55.6	70	49.9
	Fukui	179	51.5	44	51.8
	Yamanashi	243	53.0	50	56.1
	Nagano	701	51.9	131	51.7
	Gifu	591	51.8	139	51.9
	Shizuoka	1177	51.3	274	51.5
	Aichi	2866	51.5	705	51.2
	Mie	577	52.0	160	49.1
Western Japan	Shiga	561	51.4	118	50.1
	Kyoto	1274	51.1	346	51.2
	Osaka	3194	51.8	944	50.8
	Hyogo	2066	51.9	581	51.3
	Nara	448	53.4	115	53.6
	Wakayama	217	52.1	68	54.1
	Tottori	138	51.6	39	49.6

For peer review only

3

Shimane	155	51.8	47	51.2
Okayama	576	51.7	162	52.4
Hiroshima	993	52.4	210	51.2
Yamaguchi	357	52.8	110	51.0
Tokushima	187	50.4	62	48.5
Kagawa	312	50.9	87	51.5
Ehime	367	50.1	111	50.2
Kouchi	182	54.5	60	54.5
Fukuoka	1479	51.7	418	50.9
Saga	175	52.4	44	46.5
Nagasaki	309	51.2	87	52.7
Kumamoto	397	50.5	111	49.2
Oita	260	52.6	68	51.1
Miyazaki	204	51.6	60	47.1
Kagoshima	331	49.8	100	49.7
Okinawa	363	47.0	167	45.7

For peer review only

Supplementary Table 2. Monthly means (and standard errors) of home morning blood pressure from September 2013 to September 2015.

		Men					Women					Temp
		N	Systolic BP [mmHg]		Diastolic BP [mmHg]		N	Systolic BP [mmHg]		Diastolic BP [mmHg]		[C]
			Mean	SE	Mean	SE		Mean	SE	Mean	SE	
2013	Sep	13184	130.1	0.12	82.9	0.09	2563	123.6	0.31	76.4	0.22	22.1
	Oct	13779	131.7	0.12	83.9	0.09	2681	126.2	0.31	78.2	0.22	17.7
	Nov	13878	134.2	0.12	85.3	0.09	2644	128.6	0.31	79.6	0.22	10.0
	Dec	13997	134.6	0.12	85.5	0.09	2677	129.0	0.30	79.8	0.21	5.1
2014	Jan	14578	134.4	0.11	85.2	0.09	2752	128.9	0.31	79.7	0.21	2.7

For peer review only

Feb	14702	134.1	0.11	85.0	0.09	2782	128.2	0.30	79.3	0.21	3.1
Mar	15689	133.3	0.11	84.6	0.09	2954	127.4	0.28	78.7	0.20	7.0
Apr	16178	132.5	0.11	84.2	0.08	3071	126.6	0.28	78.2	0.20	11.4
May	16720	130.8	0.10	83.3	0.08	3202	124.8	0.27	77.2	0.19	17.2
Jun	16887	129.5	0.10	82.4	0.08	3224	123.3	0.26	76.0	0.19	21.2
Jul	17005	128.7	0.10	82.0	0.08	3238	122.3	0.26	75.4	0.20	24.4
Aug	17092	128.9	0.10	82.1	0.08	3261	122.4	0.26	75.5	0.20	25.3
Sep	17528	130.8	0.10	83.5	0.08	3423	124.4	0.26	77.0	0.19	20.7
Oct	17744	132.2	0.10	84.4	0.08	3439	126.5	0.26	78.4	0.19	16.2
Nov	17690	133.4	0.10	85.0	0.08	3378	127.9	0.27	79.3	0.19	11.2
Dec	17382	134.8	0.10	85.7	0.08	3295	129.4	0.28	80.0	0.19	4.4

For peer review only

2015	Jan	18133	134.2	0.10	85.3	0.08	3472	128.2	0.27	79.3	0.18	3.6
	Feb	18483	133.8	0.10	85.1	0.08	3537	127.9	0.26	79.3	0.18	3.5
	Mar	19266	133.1	0.10	84.8	0.08	3740	127.3	0.26	79.2	0.18	7.2
	Apr	20073	131.8	0.09	84.2	0.07	3901	125.9	0.25	78.4	0.17	12.7
	May	21140	130.4	0.09	83.4	0.07	4062	124.4	0.24	77.5	0.17	18.2
	Jun	21512	129.7	0.09	83.1	0.07	4211	123.5	0.23	77.1	0.17	20.3
	Jul	21532	128.6	0.09	82.5	0.07	4112	122.1	0.23	76.2	0.18	24.3
	Aug	21388	129.2	0.09	82.8	0.07	4164	122.8	0.24	76.6	0.17	25.1
	Sep	21408	130.3	0.09	83.7	0.07	4211	124.1	0.24	77.7	0.17	20.9

BP: blood pressure, C: degree Celsius, SE: Standard error, Temp: temperature, Jan: January, Feb: February, Mar: March, Apr: April, Jun: June, Jul: July, Aug: August, Sep: September, Oct: October, Nov: November, Dec: December

For peer review only

Supplementary Table 3. Monthly means (and standard errors) of home evening blood pressure from September 2013 to September 2015.

		Men					Women					Temp
		N	Systolic BP [mmHg]		Diastolic BP [mmHg]		N	Systolic BP [mmHg]		Diastolic BP [mmHg]		[C]
			Mean	SE	Mean	SE		Mean	SE	Mean	SE	
2013	Sep	10993	125.1	0.14	77.2	0.11	2210	120.2	0.34	72.6	0.24	22.8
	Oct	11588	126.4	0.13	78.2	0.11	2340	122.6	0.34	74.5	0.24	18.3
	Nov	11713	128.6	0.14	79.6	0.11	2362	124.7	0.33	76.0	0.24	11.1
	Dec	11872	128.8	0.14	79.6	0.11	2411	125.0	0.33	75.9	0.23	6.3
2014	Jan	12518	129.1	0.13	79.8	0.10	2519	125.2	0.32	76.0	0.23	4.1

For peer review only

8

Feb	12500	128.5	0.13	79.3	0.10	2481	124.5	0.32	75.6	0.23	4.6
Mar	13513	128.1	0.12	79.1	0.10	2689	124.3	0.31	75.3	0.22	8.4
Apr	13553	127.0	0.12	78.5	0.10	2660	123.1	0.31	74.5	0.22	12.8
May	13993	125.2	0.12	77.3	0.10	2778	121.4	0.30	73.2	0.22	17.7
Jun	14008	124.1	0.12	76.5	0.10	2695	120.2	0.30	72.1	0.21	21.5
Jul	13855	123.6	0.12	76.1	0.10	2710	119.3	0.30	71.7	0.22	24.9
Aug	13953	124.5	0.12	76.7	0.10	2730	120.1	0.30	72.1	0.22	25.4
Sep	14328	125.3	0.12	77.5	0.10	2837	121.0	0.30	73.2	0.22	21.6
Oct	14614	126.7	0.12	78.5	0.09	2902	123.4	0.30	75.0	0.21	17.2
Nov	14592	128.0	0.12	79.4	0.10	2816	125.0	0.32	76.0	0.22	12.4
Dec	14392	128.9	0.12	79.7	0.10	2761	125.8	0.32	76.4	0.23	5.2

For peer review only

2015	Jan	15193	129.0	0.12	79.7	0.09	3020	125.1	0.30	75.9	0.21	4.5
	Feb	15374	128.7	0.12	79.7	0.09	3001	124.9	0.30	76.0	0.21	4.8
	Mar	15975	128.0	0.11	79.3	0.09	3183	123.9	0.29	75.6	0.21	8.6
	Apr	16447	126.7	0.11	78.5	0.09	3258	123.3	0.29	75.2	0.20	13.4
	May	17167	125.1	0.11	77.5	0.09	3394	120.7	0.27	73.5	0.19	18.7
	Jun	17282	124.7	0.11	77.4	0.09	3452	120.4	0.27	73.4	0.20	20.5
	Jul	17143	124.1	0.11	76.9	0.09	3306	119.4	0.27	72.8	0.21	24.6
	Aug	16891	124.9	0.11	77.5	0.09	3317	120.4	0.27	73.3	0.20	25.4
	Sep	16927	125.4	0.11	78.1	0.09	3378	121.3	0.27	74.1	0.20	21.3

BP: blood pressure, C: degree Celsius, SE: Standard error, Temp: temperature, Jan: January, Feb: February, Mar: March, Apr: April, Jun: June, Jul: July, Aug: August, Sep: September, Oct: October, Nov: November, Dec: December

For peer review only

Supplementary Table 4. Monthly means (and standard errors) of home morning blood pressure by age groups (ages less than 60 years) from September 2013 to September 2015.

		Men					Women					Temp
		N	Systolic BP [mmHg]		Diastolic BP [mmHg]		N	Systolic BP [mmHg]		Diastolic BP [mmHg]		[C]
			Mean	SE	Mean	SE		Mean	SE	Mean	SE	
2013	Sep	9546	130.2	0.14	84.7	0.11	1706	122.1	0.39	77.5	0.29	22.1
	Oct	9972	131.7	0.14	85.7	0.11	1782	124.7	0.39	79.4	0.28	17.7
	Nov	10003	134.0	0.14	87.1	0.11	1748	126.5	0.38	80.6	0.28	10.0
	Dec	10054	134.6	0.14	87.3	0.11	1736	127.0	0.38	81.0	0.28	5.1
2014	Jan	10461	134.3	0.14	87.0	0.10	1808	126.8	0.37	80.9	0.28	2.7

For peer review only

Feb	10570	134.1	0.14	86.8	0.10	1812	126.0	0.37	80.4	0.28	3.1
Mar	11211	133.4	0.13	86.5	0.10	1915	125.2	0.35	79.7	0.26	7.0
Apr	11556	132.7	0.13	86.1	0.10	1971	124.2	0.34	79.3	0.26	11.3
May	11925	131.0	0.12	85.2	0.10	2079	122.6	0.33	78.2	0.25	17.2
Jun	11961	129.8	0.13	84.5	0.10	2111	121.6	0.33	77.3	0.25	21.2
Jul	12008	129.2	0.12	84.1	0.10	2119	120.9	0.34	76.7	0.26	24.4
Aug	11991	129.5	0.12	84.3	0.10	2129	121.0	0.34	76.9	0.26	25.3
Sep	12338	131.1	0.13	85.6	0.10	2254	122.6	0.33	78.2	0.25	20.7
Oct	12464	132.4	0.12	86.4	0.09	2245	124.8	0.33	79.7	0.25	16.2
Nov	12421	133.5	0.12	87.0	0.09	2174	126.0	0.34	80.6	0.26	11.2
Dec	12112	135.0	0.13	87.8	0.10	2097	127.4	0.35	81.3	0.26	4.4

For peer review only

2015	Jan	12672	134.3	0.12	87.2	0.09	2239	126.1	0.34	80.4	0.24	3.6
	Feb	12922	133.9	0.12	87.0	0.09	2309	125.7	0.33	80.2	0.24	3.5
	Mar	13438	133.3	0.12	86.8	0.09	2451	125.4	0.32	80.3	0.23	7.2
	Apr	14019	132.0	0.11	86.1	0.09	2568	124.0	0.32	79.5	0.23	12.7
	May	14879	130.6	0.11	85.3	0.08	2712	122.8	0.30	78.8	0.22	18.2
	Jun	15186	130.0	0.11	85.0	0.08	2826	122.0	0.29	78.5	0.22	20.3
	Jul	15141	129.2	0.11	84.5	0.08	2785	121.1	0.30	77.8	0.23	24.3
	Aug	14955	129.7	0.11	84.9	0.08	2816	121.4	0.30	77.9	0.22	25.1
	Sep	14986	130.6	0.11	85.8	0.08	2844	122.3	0.30	78.9	0.21	20.9

BP: blood pressure, C: degree Celsius, SE: Standard error, Temp: temperature, Jan: January, Feb: February, Mar: March, Apr: April, Jun: June, Jul: July, Aug: August, Sep: September, Oct: October, Nov: November, Dec: December

For peer review only

Supplementary Table 5. Monthly means (and standard errors) of home morning blood pressure by age groups (ages 60 years or older) from September 2013 to September 2015.

		Men					Women					Temp
		N	Systolic BP [mmHg]		Diastolic BP [mmHg]		N	Systolic BP [mmHg]		Diastolic BP [mmHg]		[C]
			Mean	SE	Mean	SE		Mean	SE	Mean	SE	
2013	Sep	3638	129.7	0.22	78.0	0.15	857	126.7	0.49	74.2	0.31	22.1
	Oct	3807	131.9	0.22	79.3	0.15	899	129.3	0.48	75.9	0.31	17.7
	Nov	3875	134.6	0.22	80.7	0.15	896	132.6	0.50	77.6	0.32	10.0
	Dec	3943	134.6	0.22	80.8	0.15	941	132.5	0.47	77.6	0.30	5.0
2014	Jan	4117	134.7	0.21	80.8	0.15	944	133.0	0.50	77.5	0.31	2.8

For peer review only

Feb	4132	134.1	0.21	80.4	0.15	970	132.3	0.49	77.4	0.31	3.1
Mar	4478	133.1	0.20	80.0	0.14	1039	131.4	0.43	77.0	0.29	7.1
Apr	4622	132.1	0.19	79.5	0.14	1100	130.8	0.45	76.5	0.28	11.4
May	4795	130.2	0.18	78.5	0.13	1123	129.0	0.43	75.2	0.27	17.1
Jun	4926	128.5	0.18	77.5	0.13	1113	126.7	0.41	73.7	0.26	21.2
Jul	4997	127.4	0.18	76.8	0.13	1119	125.1	0.40	72.9	0.27	24.5
Aug	5101	127.6	0.18	77.0	0.13	1132	125.0	0.41	72.9	0.27	25.3
Sep	5190	129.9	0.18	78.5	0.13	1169	127.9	0.43	74.6	0.27	20.7
Oct	5280	131.9	0.18	79.7	0.13	1194	129.8	0.42	76.0	0.26	16.3
Nov	5269	133.0	0.18	80.3	0.13	1204	131.2	0.42	77.0	0.27	11.2
Dec	5270	134.6	0.18	81.0	0.13	1198	132.9	0.44	77.8	0.27	4.3

For peer review only

2015	Jan	5461	134.1	0.18	80.8	0.12	1233	132.2	0.42	77.3	0.26	3.5
	Feb	5561	133.6	0.17	80.7	0.12	1228	131.9	0.43	77.4	0.27	3.5
	Mar	5828	132.7	0.17	80.2	0.12	1289	131.0	0.41	77.1	0.26	7.2
	Apr	6054	131.4	0.16	79.5	0.12	1333	129.4	0.39	76.2	0.25	12.7
	May	6261	130.0	0.16	78.8	0.12	1350	127.5	0.37	74.9	0.24	18.1
	Jun	6326	129.0	0.16	78.4	0.11	1385	126.5	0.36	74.3	0.24	20.3
	Jul	6391	127.3	0.15	77.5	0.11	1327	124.3	0.37	73.0	0.25	24.3
	Aug	6433	128.0	0.15	77.8	0.11	1348	125.8	0.38	73.8	0.25	25.0
	Sep	6422	129.5	0.15	79.0	0.11	1367	127.9	0.38	75.3	0.25	20.9

BP: blood pressure, C: degree Celsius, SE: Standard error, Temp: temperature, Jan: January, Feb: February, Mar: March, Apr: April, Jun: June, Jul: July, Aug: August, Sep: September, Oct: October, Nov: November, Dec: December

For peer review only

STROBE Statement—checklist of items that should be included in reports of observational studies

	Item No	Recommendation	Page/Line Number
**Title and abstract**	1	(*a*) Indicate the study's design with a commonly used term in the title or the abstract	Page 3 / lines 9-18
		(*b*) Provide in the abstract an informative and balanced summary of what was done and what was found	Page 3 / line 2 – Page 4 / line 12
**Introduction**			
Background/rationale	2	Explain the scientific background and rationale for the investigation being reported	Page 6 / line 2 – Page7 / line 2
Objectives	3	State specific objectives, including any prespecified hypotheses	Page 7 / lines 3 –8
**Methods**			
Study design	4	Present key elements of study design early in the paper	Page 7 / line 9 – Page 10 / line 4
Setting	5	Describe the setting, locations, and relevant dates, including periods of recruitment, exposure, follow-up, and data collection	Page 7 / line 9 – Page 9 / line 13
Participants	6	(*a*) *Cohort study*—Give the eligibility criteria, and the sources and methods of selection of participants. Describe methods of follow-up<br>*Case-control study*—Give the eligibility criteria, and the sources and methods of case ascertainment and control selection. Give the rationale for the choice of cases and controls<br>*Cross-sectional study*—Give the eligibility criteria, and the sources and methods of selection of participants	Page 9 / line 12 – Page 10 / line 1
		(*b*) *Cohort study*—For matched studies, give matching criteria and number of exposed and unexposed<br>*Case-control study*—For matched studies, give matching criteria and the number of controls per case	NA
Variables	7	Clearly define all outcomes, exposures, predictors, potential confounders, and effect modifiers. Give diagnostic criteria, if applicable	Page 10 / lines 2 – 14
Data sources/ measurement	8*	For each variable of interest, give sources of data and details of methods of assessment (measurement). Describe comparability of assessment methods if there is more than one group	Page 7 / line 9 – Page 10 / line 7
Bias	9	Describe any efforts to address potential sources of bias	Page 8 / lines 9 – 13, Page 10 / line 15 – Page 11 / line 1
Study size	10	Explain how the study size was arrived at	Page 9 / line 12 – Page 10 / line 1
Quantitative variables	11	Explain how quantitative variables were handled in the analyses. If applicable, describe which groupings were chosen and why	NA
Statistical methods	12	(*a*) Describe all statistical methods, including those used to control for confounding	Page 10 / lines 8 – 14
		(*b*) Describe any methods used to examine subgroups and interactions	Page 10 / lines 8 – 10
		(*c*) Explain how missing data were addressed	Page 9 / line 12 – Page 10 / line 1
		(*d*) *Cohort study*—If applicable, explain how loss to follow-up was addressed<br>*Case-control study*—If applicable, explain how matching of cases and controls was addressed<br>*Cross-sectional study*—If applicable, describe analytical methods taking account of sampling strategy	Page 9 / line 12 – Page 10 / line 14

For peer review only

		(*e*) Describe any sensitivity analyses	Page 10 / lines 8-10
**Results**			
Participants	13*	(a) Report numbers of individuals at each stage of study—eg numbers potentially eligible, examined for eligibility, confirmed eligible, included in the study, completing follow-up, and analysed	Page 11 / lines 4 – 6
		(b) Give reasons for non-participation at each stage	NA
		(c) Consider use of a flow diagram	NA
Descriptive data	14*	(a) Give characteristics of study participants (eg demographic, clinical, social) and information on exposures and potential confounders	Page 11 / lines 4-6, Supplemental Table 1
		(b) Indicate number of participants with missing data for each variable of interest	NA
		(c) *Cohort study*—Summarise follow-up time (eg, average and total amount)	NA
Outcome data	15*	*Cohort study*—Report numbers of outcome events or summary measures over time	NA
		Case-control study—Report numbers in each exposure category, or summary measures of exposure	NA
		Cross-sectional study—Report numbers of outcome events or summary measures	Supplemental Table 2-5
Main results	16	(*a*) Give unadjusted estimates and, if applicable, confounder-adjusted estimates and their precision (eg, 95% confidence interval). Make clear which confounders were adjusted for and why they were included	Page 11 / lines 8-16, Supplemental Table 1-5
		(*b*) Report category boundaries when continuous variables were categorized	NA
		(*c*) If relevant, consider translating estimates of relative risk into absolute risk for a meaningful time period	NA
Other analyses	17	Report other analyses done—eg analyses of subgroups and interactions, and sensitivity analyses	NA
**Discussion**			
Key results	18	Summarise key results with reference to study objectives	Page 14 / lines 12-18
Limitations	19	Discuss limitations of the study, taking into account sources of potential bias or imprecision. Discuss both direction and magnitude of any potential bias	Page 15 / line 18– Page 16 / line 6
Interpretation	20	Give a cautious overall interpretation of results considering objectives, limitations, multiplicity of analyses, results from similar studies, and other relevant evidence	Page 14 / line 19 – Page 16 / line 14
Generalisability	21	Discuss the generalisability (external validity) of the study results	Page 16 / lines 7–14
**Other information**			
Funding	22	Give the source of funding and the role of the funders for the present study and, if applicable, for the original study on which the present article is based	Page 21 / lines 5-16

*Give information separately for cases and controls in case-control studies and, if applicable, for exposed and unexposed groups in cohort and cross-sectional studies.

Note: An Explanation and Elaboration article discusses each checklist item and gives methodological background and published examples of transparent reporting. The STROBE checklist is best used in conjunction with this article (freely available on the Web sites of PLoS Medicine at http://www.plosmedicine.org/, Annals of Internal Medicine at http://www.annals.org/, and Epidemiology at http://www.epidem.com/). Information on the STROBE Initiative is available at www.strobe-statement.org.

For peer review only
