# Supplementary material for: Seasonal variation in home blood pressure: findings from nationwide web-based monitoring in Japan
Source: BMJ Open. 2018 Jan 5;8(1):e017351. doi: 10.1136/bmjopen-2017-017351 (PMC5780696; doi:10.1136/bmjopen-2017-017351)
Supplement: Reviewer comments [file bmjopen-2017-017351.reviewer_comments.pdf]

PEER REVIEW HISTORY

BMJ Open publishes all reviews undertaken for accepted manuscripts. Reviewers are asked to complete a checklist review form (**http://bmjopen.bmj.com/site/about/resources/checklist.pdf**) and are provided with free text boxes to elaborate on their assessment. These free text comments are reproduced below.

ARTICLE DETAILS

**TITLE (PROVISIONAL)**	Seasonal variation in home blood pressure: findings from nationwide web-based monitoring in Japan
**AUTHORS**	Iwahori, Toshiyuki; Miura, katsuyuki; Obayashi, Keiichi; Ohkubo, Takayoshi; Nakajima, Hiroshi; Shiga, Toshikazu; Ueshima, Hirotsugu

VERSION 1 – REVIEW

**REVIEWER**	George Stergiou<br>Hypertension Center STRIDE-7<br>University of Athens, Greece<br>CONFIDENTIAL FOR THE EDITORS<br>(found no box to place)<br>Numerous studies have shown the seasonal variation in blood pressure in hypertensive patients and in the general population. Key features of this study are the use of web-based system to collect blood pressure readings from electronic home monitors and the large sample size. However, there are several methodological issues and the blood pressure monitoring protocol was not standardized (devices, monitoring schedule, conditions, readings per subject and per season, etc). Plus, this article does not provide any novel information.<br>Received lecturer fees by Omron.<br>Performed validation studies for several blood pressure monitor manufacturers.<br>Received consultation fees by several blood pressure monitor manufacturers.
**REVIEW RETURNED**	17-May-2017

**GENERAL COMMENTS**	The study provides data on the seasonal variation in home blood pressure in 64,536 subjects through a nationwide web-based monitoring system in Japan, which obtained blood pressure readings automatically from electronic home monitors. The data showed the typical seasonal variation of blood pressure and temperature with higher blood pressure values in winter.<br><br>COMMENTS<br><br>1. The authors should provide more data in regard to the standardization of home blood pressure monitoring (monitoring schedule, following the manual instructions, devices, validation, arm versus wrist, the conditions of measurement, frequency of measurements per participants (minimum and range) and per season.

	2. It would be a much cleaner dataset if subjects who contributed with a minimum number of readings per season have been included.<br>3. How can the authors exclude that some devices have been used by different people at different periods? This is a potential limitation.<br>4. There should be a selection bias in this study that might have affected the results, e.g. those who registered versus those who did not, gender selection with more men, etc. The analysis of average blood pressure of different schedules from different subjects at different seasons further aggravates the selection bias.<br>5. Appropriate statistical testing should be performed for all comparisons in the Results.<br>6. Please, provide more details about the age distribution in your sample.<br>7. Please provide clearer description on how individual morning and evening average blood pressures were calculated.<br>8. The authors should cite important relevant studies, especially the prospective ones.<br>9. What is the 'spot arm' blood pressure monitor?

**REVIEWER**	Eamon Dolan<br>Connolly hospital,<br>Dublin 15,<br>Ireland
**REVIEW RETURNED**	22-May-2017

**GENERAL COMMENTS**	This work is of interest given the sample size and novel technique used to capture the home BP data.<br>I have some methodological points to make.<br>Could the authors list what data beyond age, gender and BP meds they have to hand.<br>If that could be described in a table for the reader it would help understanding.<br>What is the average number of readings entered by an individual. ie over what period. Was there a cut-off. i.e. if an individual only entered 1 set of readings etc.<br>Obviously the data has more interest if it was the same cohort of subjestx who had their BP measured across the year.<br>Does BP medications blunt the effect?<br>I would like to see the cohort divided into 3 age groups to evaluate trends.<br><br>The methods section needs to be toned down to stop it reading like an advertising brochure. - "The devices currently employed (but still expanding)" etc<br><br>Analysis on visit to vist variability would be of interest to the reader.

REVIEWER	Peter Avery<br>School of Mathematics & Statistics,<br>Newcastle University,<br>Newcastle upon Tyne,UK
REVIEW RETURNED	05-Jul-2017

GENERAL COMMENTS	This is a very interesting dataset but essentially no statistical analysis has been done on it. They have merely presented means and standard errors. It is clear that temperature is a good predictor of mean BP but it is not clear if the temperature in current month or the previous month is the best predictor. Age is handled by dichotomising which throws away a lot of information. Gender is important but again we cannot see if it significantly interacts with any temperature and age effect. Means are presented by area but we cannot see if there is any area effect or whether that is merely explained by temperature differences. I would recommend analysing means using weighted regression. This could be done by averaging over ages and for individual age/gender combinations. The authors should clearly seek statistical advice.

VERSION 1 – AUTHOR RESPONSE

Review Report (Reviewer 1)

>1. The authors should provide more data in regard to the standardization of home blood pressure monitoring (monitoring schedule, following the manual instructions, devices, validation, arm versus wrist, the conditions of measurement, frequency of measurements per participants (minimum and range) and per season.

Response: We agree with the reviewer that the blood pressure measured in clinical settings and also home blood pressure measurements should be standardized to identify individual’s values for treatment. However, the scope of this study is not on evaluating the seasonal variation of blood pressure in clean, controlled and clinical data setting. The aim of the study was to confirm whether the data collected by this web-based healthcare platform have the potential to support the public health authorities to make appropriate policies at the right time to prevent non-communicable diseases. Thus, we would like to emphasize the uniqueness of this study.

The “real world” data obtained in the general population contain biases and noises, and these are mostly unstandardized, uncontrolled, and obtained under non-clinical environment. For instance, our data contain diverse noises related to the monitoring schedule, by whom measured, whether the participants followed the manual instructions or not, devices used, validation level for each devices, arm versus wrist, conditions of measurements at home, and the frequency of measurements per participants, etc. as this reviewer had pointed out. However, the main result of the present study which was even obtained under “real world” data shows consistencies with the previous studies which were done under standardized conditions in clinical settings.

Nowadays, clinicians are in charge of individual’s treatment, and public health authorities are in charge of disease prevention and control. However, it is not easy for public health authorities to recognize the real time population blood pressure for disease prevention and control. Therefore, our study here to highlight how we can use “real world” data for disease prevention and control.

It is reasonable to infer that the main result presented here shows the strong evidence related to the seasonal variation of blood pressure observed at home and office since the seasonal variation of the blood pressure obtained in the present study showed similar pattern compared to those obtained in the previous studies.

Our findings would provide an important message for population blood pressure control. The "real world" data (the data collected by this web-based healthcare platform) may support the public health authorities to make appropriate policies at the right time to prevent and control non-communicable diseases.

As mentioned on Page 8, lines 10-12, the participants received the manual for BP measurement for standardization; however, we could not monitor whether they followed the manual appropriately. This would be the limitation for standardization in the present study.

We added statements to emphasize this point.

"Nowadays, these newly established technologies and platforms enable to collect real world data simultaneously and show the possibilities of lowering the hurdles to detect useful population-wide information and provide new approach for public health authorities.
Multiple observational studies have examined the seasonal variation of BP in clinical settings; 10 however, there is no report on seasonal variation using nationwide large sample of home BP monitoring data from non-medical and uncontrolled settings. Thus, our aims were to assess seasonal variation in home blood pressure among free-living nationwide users by real world data collection by this web-based health care platform, and to see the applications for public health policy making." (Page 6, line 19 – Page 7, line 9)

"Thus, acquiring nationwide "real world" home BP data is desirable." (Page 14, lines 5 – 6)

"Thus, we demonstrated the practical use of web-based healthcare platform which may reliably reflect the trend of population-wide BP as a "real world" data here in weekly and monthly analyses." (Page 15, lines 1 – 4)

"In the present study, a parallel association was observed between seasonal variation in home BP (about 7 mmHg) and changes in outdoor temperature in this "real world" data." (Page 15, lines 7 –8)

"Ideally, public health authorities would be able to notice population-wide changes in BP through monitoring population-wide "real world" home BP data by using this platform, and this would allow them to forecast CVD risk at appropriate times. Furthermore, public health authorities may also make announcements for clinicians, and practitioners to be cautious of over- and under-dose of anti-hypertensive medication by referencing the population-wide "real world" BP which was easily screened for appropriate BP control." (Page 15, line 15 – Page 16, line 2)

"To the best of our knowledge, this is the first study to report seasonal variation from a large sample of "real world" home BP data from non-medical settings. Our findings demonstrated that the seasonal variation of "real world" home BP was independent of age. In this study, the participants were whom registered to use this web based healthcare platform; the participants might have higher socioeconomic status since they may have higher computer literacy and much health conscious people compared to others in general populations. Therefore, the seasonal variation of the "real world" home BP might have been underestimated due to the higher socioeconomic status of the study participants. Moreover, the participants in this web based healthcare platform can register and leave by their freewill in anytime, this may aggravates the selection bias."(Page 16, line 11 – Page 17, line 2)

“As the data obtained during the study was “real world” data, the room temperature during home BP measurements was unknown, some of the BP measurement devices might have been used by multiple users without differentiating the user identification numbers and the frequency and timing of BP measurements may have differed for each individual.” (Page 17, lines 7 – 11)

We also changed the title to cover this point.

“Seasonal variation in home blood pressure: findings from nationwide web-based monitoring in Japan” (Page 1, lines 2 – 3)

>2. It would be a much cleaner dataset if subjects who contributed with a minimum number of readings per season have been included.

Response: The participants in this web based healthcare platform were able to register and leave by their freewill in anytime. Thus, the lengths of the period of active blood pressure measurement by each participant were diverse. However, the histogram of the number of readings in daily and monthly BP measurements in home morning systolic BP (MSBP) and evening systolic BP (ESBP) in Sept. 2014 are shown in Supplemental Figures A1-A2 of "Response to the reviewers" document. Large and small peaks appeared around close to one, 30, 60 and 90 in monthly data. Thus, most of the participants measured BP intermittently, but some of the participants measured BP routinely once, twice or even 3 times per day for MSBP or ESBP measurements. Most of the average numbers of readings entered by an individual were once per a day for MSBP and ESBP throughout the period. During the other month, the pattern was similar. Therefore, we did not see any difference among four seasons.

>3. How can the authors exclude that some devices have been used by different people at different periods? This is a potential limitation.

Response: We agree with this reviewer’s comment. The “real world” data obtained in the general population contain biases and noises, and these are mostly unstandardized, uncontrolled, and obtained under non-clinical environment. We added related statement as below.

“These devices can be share used with family members by differentiating the user identification numbers set at each device among family members.” (Page 8, lines 6-8)

“As the data obtained during the study was “real world” data, the room temperature during home BP measurements was unknown, some of the BP measurement devices might have been used by multiple users without differentiating the user identification numbers and the frequency and timing of BP measurements may have differed for each individual.” (Page 17, lines 7-11)

However, the uniqueness of this study stands at the point that the seasonal variation in “real world” blood pressure was consistent with the previous findings related to seasonal variation of blood pressure observed at home and office which was done under tight standardization of blood pressure measurement. We added statements to emphasize this point to cover this point in the answers for previous question #1.

>4. There should be a selection bias in this study that might have affected the results, e.g. those who registered versus those who did not, gender selection with more men, etc. The analysis of average blood pressure of different schedules from different subjects at different seasons further aggravates the selection bias.

Response: In this study, the participants were whom registered to use this web based healthcare platform. Thus, the participants might have higher computer literacy than others in general populations.

The mean age of the study participants was 54 years old. According to PMID: 23902998 (Circ J. 2013;77(9):2226-31.), the treatment rate among hypertensive people in Japan was 31-69% in individuals, ages 50-79. In the present study, approximately 39% of the participant reported taking anti-hypertensive medication and remained stable throughout the study period (see Supplemental Figure A3 of "Response to the reviewers" document). Thus, the study population tends to be health conscious people rather than individuals under treatment.

The mean age of the study participants were approximately 54 years old and remained stable throughout the study period (see Supplemental Figure A4 of "Response to the reviewers" document).

Furthermore, the ratio of the men in the participants were approximately 83% of the overall participants and the ratio also remained stable throughout the study period (see Supplemental Figure A5 of "Response to the reviewers" document).

Since the participants in this web based healthcare platform can register and leave by their freewill in anytime, this may aggravates the selection bias as the reviewer had pointed out. However, the study was large enough to allow measurements which make a chance finding less likely.

We added statements to emphasize this point to cover this reviewer’s request.

“Approximately 83% of the study participants were men, mean age was 54 years old, and the treatment rate among hypertensive people in the present study was approximately 39%; however these status remained relatively stable throughout the study period.” (Page 11, lines 10 – 13)

“Our findings demonstrated that the seasonal variation of “real world” home BP was independent of age. In this study, the participants were whom registered to use this web based healthcare platform; the participants might have higher socioeconomic status since they may have higher computer literacy and much health conscious people compared to others in general populations. Therefore, the seasonal variation of the “real world” home BP might have been underestimated due to the higher socioeconomic status of the study participants. Moreover, the participants in this web based healthcare platform can register and leave by their freewill in anytime, this may aggravates the selection bias.” (Page 16, line 12 – Page 17, line 2)

>5. Appropriate statistical testing should be performed for all comparisons in the Results.

Response: As mentioned in the Figure legends, we have already shown the very short bars indicating the mean value +/- standard error for Figures 1-3. It is apparent that the bars shown in Figures 1-2 will not overlap each other among two different subgroups even if we show the results in 95% confidence interval.

However, we newly carried out statistical testing to see whether there is a statistical differences observed in BP among each month in Figure 3.

We added statements as shown below.

“Intermonth differences in BP were assessed analysis of variance. The t test for unpaired observations was used to determine the significance of the difference, after Bonferroni correction for multiple comparisons. All P values were two sided, and P values < 0.05 was taken as the level of statistical significance.” (Page 10, lines 12-16)

“There were statistical significances observed in BP between summer and winter months; however, there were a few intermonth combinations which did not reach statistical significance (March versus November, and May versus September).” (Page 13, lines 4-7)

>6. Please, provide more details about the age distribution in your sample.

Response: The histogram of the age distribution is as shown in Supplemental Figure A6 of "Response to the reviewers" document. We added statement related to age as shown below in the text.

” Approximately 83% of the study participants were men, mean age was 54 years old, and the treatment rate among hypertensive people in the present study was approximately 39%; however these status remained relatively stable throughout the study period.” (Page 11, lines 10-13)

>7. Please provide clearer description on how individual morning and evening average blood pressures were calculated.

Response: We have already mentioned on Page 10, lines 2-7 in the text; however, we added related statements as below to clarify the point.

“The monthly means of home BP was calculated by averaging individuals’ home BP of overall participants.” (Page 10, lines 7-8)

>8. The authors should cite important relevant studies, especially the prospective ones.

Response: We already have reference 10 (original reference 9) as the prospective ones. As this reviewer requests, we also newly added reference 9 (Hypertension. 2014 Apr;63(4):675-82.) to cite the important relevant study.

9. Stergiou GS, Asayama K, Thijs L, et al. Prognosis of white-coat and masked hypertension: International Database of HOme blood pressure in relation to Cardiovascular Outcome. Hypertension. 2014 Apr;63(4):675-82.

>9. What is the 'spot arm' blood pressure monitor?

This “spot arm blood pressure monitor” is an automatic blood pressure monitor which has a unique automatic cuff wrapping system for the upper arm. By simply inserting the arm into the cuff and supporting it with the arm rest, individuals can be assured of correct placement for each and every measurement.

Response: We revised the statement as below.

“The WellnessLINK system records BP values measured at home using oscillometric-type electronic sphygmomanometers, including an upper arm BP monitor (HEM-7510C, HEM-7500F, HEM-7324C, HEM-7320F, HEM-7280C, HEM-7270C, HEM-7250-IT; OMRON Healthcare), an upper arm BP monitor with an automatic cuff wrapping system (HEM-1025; OMRON Healthcare), and a wrist BP monitor (HEM-6321T, HEM-6320T, HEM-6310F, HEM-6300F; OMRON Healthcare).” (on Page 8, lines 1-6)

>**Review Report (Reviewer 2)**

>This work is of interest given the sample size and novel technique used to capture the home BP data. I have some methodological points to make. Could the authors list what data beyond age, gender and BP meds they have to hand. If that could be described in a table for the reader it would help understanding.

Response: The aim of the study was to confirm whether the “real world” data collected by this web-based healthcare platform have the potential to support the public health authorities to make appropriate policies at the right time to prevent non-communicable diseases.

The uniqueness of this study is that the seasonal variation in home blood pressure data was observed even in real world data setting under unstandardized, uncontrolled and non-clinical environment. For instance, our data contain diverse noises related to the monitoring schedule, by whom measured, whether the participants followed the manual instructions or not, devices used, validation level for each devices, arm versus wrist, the conditions of measurements at home, the frequency of measurements per participants, etc. as this reviewer had pointed out.

However, we can show the data of ratio of gender (men/overall) and people under BP medication throughout the study period as shown in Supplemental Figures B1-B3 of "Response to the reviewers" document. We added statements to cover these points in the text.

“Approximately 83% of the study participants were men, mean age was 54 years old, and the treatment rate among hypertensive people in the present study was approximately 39%; however these status remained relatively stable throughout the study period.” (Page 11, lines 10 – 13)

“Our findings demonstrated that the seasonal variation of “real world” home BP was independent of age. In this study, the participants were whom registered to use this web based healthcare platform; the participants might have higher socioeconomic status since they may have higher computer literacy and much health conscious people compared to others in general populations. Therefore, the seasonal variation of the “real world” home BP might have been underestimated due to the higher socioeconomic status of the study participants. Moreover, the participants in this web based healthcare platform can register and leave by their freewill in anytime, this may aggravates the selection bias.” (Page 16, line 12 – Page 17, line 2)

>What is the average number of readings entered by an individual. ie over what period. Was there a cut-off. i.e. if an individual only entered 1 set of readings etc.

Response: The participants in this web based healthcare platform were able to register and leave by their freewill in anytime. Thus, the lengths of the period of active blood pressure measurement by each participant were diverse. However, the histogram of the number of readings in daily and monthly BP measurements in home morning systolic BP (MSBP) and evening systolic BP (ESBP) in Sept. 2014 are shown in Supplemental Figures B4-B5 of "Response to the reviewers" document. Large and small peaks appeared around close to zero, 30, 60 and 90 in monthly data. Thus, most of the participants measured BP intermittently, but some of the participants measured BP routinely once, twice or even 3 times per day for MSBP and ESBP. Most of the average numbers of readings entered by an individual were once per a day for MSBP and ESBP throughout the period.

>Obviously the data has more interest if it was the same cohort of subjestx who had their BP measured across the year.

Response: The uniqueness of this study stands at the point that the seasonal variation in home blood pressure data was observed even in the “real world” data setting under unstandardized, uncontrolled and non-clinical environment. The participants in this web based healthcare platform were able to register and leave by their freewill in anytime. As we presented the histogram of the number of readings in daily and monthly BP measurements in the previous question from this reviewer, most of the average numbers of readings entered by an individual were once per a day for morning systolic BP (MSBP) and evening systolic BP (ESBP) throughout the period. However, the lengths of the period of active blood pressure measurement by each participant were diverse. Therefore, we could not further analysis on the reviewer’s request with appropriate protocol and sample size. However, we would like to focus on this topic in future when we have sufficient sample size.

>Does BP medications blunt the effect?

Response: The seasonal variation of the participants with BP medication (mean ages 54) and without BP medication subgroup (mean ages 54) showed similar pattern in morning systolic BP (MSBP) and evening systolic BP (ESBP) compared to overall participants; however the summer-winter difference was smaller in participants with BP medication. The detailed results are shown in "Response to the reviewers" document.

>I would like to see the cohort divided into 3 age groups to evaluate trends.

Response: We further analyzed our data. The results are shown in the "Response to the reviewers" document. The seasonal variation of the participants with age subgroup showed similar pattern even in 3 age groups (ages under 50, ages 50 to 60, ages over 60). Younger participants showed larger BP difference among gender subgroup. We would like to show the result only for this reviewer since we do not think this result will further add scientific value on this manuscript.

>The methods section needs to be toned down to stop it reading like an advertising brochure. - "The devices currently employed (but still expanding)" etc

Response: We excluded related statement from the text.

The devices currently employed (but still expanding) by the services are as follows: a home BP monitor; a body weight, and body composition monitor; a physical activity monitor (pedometer, and calorie meter); a sleep monitor; and a body temperature monitor. The services provided are for various health care items including diet, physical activity, sleep, antihypertensive health care, metabolic syndrome prevention, and women’s health care.

>Analysis on visit to vist variability would be of interest to the reader.

Response: Since the participants in this web based healthcare platform can register and leave by their freewill in anytime, we could not further analysis on “visit to visit variability” with appropriate protocol and sample size. We would like to focus on the registered participants using this system for longer period and further analysis on “visit to visit variability” in future.

>**Review Report (Reviewer 3)**

>This is a very interesting dataset but essentially no statistical analysis has been done on it. They have merely presented means and standard errors.

Response: As mentioned in the Figure legends, we have already shown the very short bars indicating the mean value +/- standard error for Figures 1-3. It is apparent that the bars shown in Figures 1-2 will not overlap each other among two different subgroups even if we show the results in 95% confidence interval. Therefore, we did not think valuable to further add statements explaining the statistical difference among these two subgroups.

However, we newly carried out statistical testing to see whether there is a statistical differences observed in BP among each month in Figure 3.

We added statements as shown below.

“Intermonth differences in BP were assessed analysis of variance. The t test for unpaired observations was used to determine the significance of the difference, after Bonferroni correction for multiple comparisons. All P values were two sided, and P values < 0.05 was taken as the level of statistical significance.” (Page 10, lines 12-16)

“There were statistical significances observed in BP between summer and winter months; however, there were a few intermonth combinations which did not reach statistical significance (March versus November, and May versus September).” (Page 13, lines 4-7)

>It is clear that temperature is a good predictor of mean BP but it is not clear if the temperature in current month or the previous month is the best predictor. Age is handled by dichotomising which throws away a lot of information. Gender is important but again we cannot see if it significantly interacts with any temperature and age effect. Means are presented by area but we cannot see if there is any area effect or whether that is merely explained by temperature differences. I would recommend analysing means using weighted regression. This could be done by averaging over ages and for individual age/gender combinations. The authors should clearly seek statistical advice.

Response: We newly carried out sub analysis on Kanto region (Tokyo metropolitan area: Tokyo, Kanagawa, Chiba, Saitama, Gunma, Tochigi and Ibaraki) to see whether there is any area effect on seasonal variation in home BP. The results are shown in Supplemental Figure C1 of "Response to the reviewers" document. Similar seasonal variation in home blood pressure (HBP) was observed even in Kanto region. Thus, we could not find area effect which affects the existence of seasonal variation of home BP in the present study. Therefore, we will not further do weighted regression analyses and add related statements in the text.

VERSION 2 – REVIEW

**REVIEWER**	George Stergiou<br>Hypertension Center STRIDE-7, Third University Department of Medicine, Sotiria Hospital, Athens, Greece
**REVIEW RETURNED**	15-Aug-2017

**GENERAL COMMENTS**	No further comments

**REVIEWER**	Dr Peter Avery<br>Newcastle University, U.K.
**REVIEW RETURNED**	11-Aug-2017

**GENERAL COMMENTS**	The authors have introduced t-tests to compare months. I am not sure how they have done this and it is not what I was wanting them to do. The results are unhelpful.<br>What I was wanting them to do was to analyse the means using weighted regression to see the effects of the various factors. For example if you take the data in supplemental table 2 on morning blood pressure. You can perform regression on the means presented, weighting on the sample sizes. You thus have 50 observations, combining the male and female data. You find there is a highly significant linear effect of temperature, a highly significant sex effect but no evidence that the decline with temperature is different between the two sexes. Thus two parallel lines best describes the data. Presenting a scatter plot of the means with the fitted lines would be very helpful. The R squared values when including a linear effect of temperature and a sex effect are 96.3% for systolic blood pressure and 97.1% for diastolic blood pressure, showing that virtually all the variability in means is explained by temperature and sex. Similar analyses could be done on evening measurements and when the data is divided by age rather than by sex.

VERSION 2 – AUTHOR RESPONSE

We had mainly focused on presenting the data of seasonal variation of the home blood pressure observationally. The comment from this reviewer was fruitful since he or she gave us an opportunity to add a new view point presenting the data of seasonal variation of the home blood pressure with statistical perspectives.

We performed multiple regression analyses and analyzed the model with age, gender, temperature and five geological locations to see the factors which may affect the association between blood pressure and temperature. The association between blood pressure and temperature showed significant inverse association independent of age, gender and geological locations (Table 1).

No interaction was observed between age and temperature (see Supplemental Table A1), gender and temperature (see Supplemental Table A2) affecting the association between blood pressure and temperature. Thus, we applied the model presented in Table 1 as a main result of multiple regression analyses to assess the association between blood pressure and temperature.

We added Table 1 and the related statements in the text to address the result of seasonal variation of the home blood pressure with statistical perspectives. However, the amounts of the tables have increased too much so we would like to show Supplemental Table A1-A2 only to the editor and this reviewer just for reference.

“Multiple regression analysis was preformed to assess whether the association between temperature and BP is independent with age (continuous variables), gender (dichotomous: men:1, women: 0) and geological locations (Northern Japan / Eastern Japan / Central Japan / Kansai region / Western Japan). Four dummy variables for five geological locations were assigned and Eastern Japan was defined as reference area.” (Page 10, lines 15-19, Method section)

“The association between home SBP and temperature showed significant inverse association which was independent of age, gender and geological locations (Table 1). Age, gender and geographic locations had no significant differences for the interaction between the BP and temperature (all P>0.05).” (Page 12, lines 4-7, Result section)

“The association between BP and temperature showed significant inverse association (winter-summer difference: approximately 7 mmHg), and was independent of other factors such as age, gender and geological locations. In addition, all these variables had no significant differences for the interaction between the BP and temperature (all P>0.05).” (Page 13, line 17- Page 14, line 4, Discussion section)

Furthermore, regression coefficients for home morning systolic blood pressure on age, gender and temperature by multiple regression analysis with weighted and unweighted by numbers of participants in each month showed similar results (Supplemental Table A3). However, the amounts of the tables have increased too much so we would like to show Supplemental Table A3 only to the editor and this reviewer just for reference.

As this reviewer had commented on the association between blood pressure and temperature whether the temperature in current month or the previous month would become as the best predictor of mean blood pressure, we further performed analyses. The regression coefficients for home morning systolic blood pressure on temperature in current month and the previous month were similar as shown in Supplemental Table A4. Since we do not think this result will further add scientific value on our original study question, we would like to present our additional findings only to the editor and this reviewer just for reference.

VERSION 3 – REVIEW

**REVIEWER**	Dr Peter Avery<br>Newcastle University, U.K.
**REVIEW RETURNED**	27-Oct-2017

**GENERAL COMMENTS**	I thank the authors for responding very well to my comments. The paper is now statistically sound and improved by the addition of the multiple regression analysis.<br>Minor comments:<br>1. Abstract - line 14, page 12 - line 5, Page 13, last line. Replace 'inverse' by 'negative'<br>2. Abstract - line 14, page 10 - lines 17 and 18, page 12 - line 6, page 14 - line 2. Replace 'geological' by 'geographical'.<br>3. Page 10- line 15. Replace 'preformed' by 'performed'.<br>4. Page 12 - line 6. Replace 'geographic' by 'geographical'.
